# The efficacy and safety of *Ginkgo biloba* L. leaves extract combined with ACEI/ARB on diabetic kidney disease: a systematic review and meta-analysis of 41 randomized controlled trials

**DOI:** 10.3389/fphar.2024.1408546

**Published:** 2025-01-03

**Authors:** Zehua Zhang, Shiyun Tang, Shiyu Liu, Yulin Leng, Xiaoxu Fu, Hongyan Xie, Hong Gao, Chunguang Xie

**Affiliations:** ^1^ Hospital of Chengdu University of Traditional Chinese Medicine, Chengdu, China; ^2^ TCM Regulating Metabolic Diseases Key Laboratory of Sichuan Province, Hospital of Chengdu University of Traditional Chinese Medicine, Chengdu, China; ^3^ Department of Endocrinology, Hospital of Chengdu University of Traditional Chinese Medicine, Chengdu, China

**Keywords:** Ginkgo biloba L. leaves extract, diabetic kidney disease, systematic review, randomized controlled trials, meta-analysis

## Abstract

**Background:**

Diabetic kidney disease (DKD) has become the leading cause of end-stage renal disease in the world. However, the current conventional approaches have not yet achieved satisfactory efficacy. As one of the most influential products in botanical medicine, *Ginkgo biloba* L. leaves extract (GBE) demonstrates various pharmacological effects on DKD and is gradually used as an adjunctive therapy for this disease. A comprehensive analysis is necessary to evaluate the efficacy and safety of GBE as an adjuvant treatment for DKD.

**Objective:**

This meta-analysis aimed to evaluate the efficacy and safety of GBE as a supplementary treatment to conventional renin-angiotensin-aldosterone system inhibitors for DKD patients, providing a reference for subsequent research and clinical practice.

**Methods:**

This study has been registered in PROSPERO as CRD42023455792. Ten databases were searched from their inception to 21 July 2023. Randomized controlled trials about GBE and DKD were included. Review Manager 5.4 and Stata 16.0 were employed to conduct the analysis. Heterogeneity was assessed through the χ^2^ test and the I^2^ test, and the effect model was chosen accordingly. Meta-regression and subgroup analysis were performed to investigate the sources of heterogeneity and the influence of different factor levels on efficacy. The publication bias was evaluated with the funnel plot and Egger’s test, and the evidence quality was evaluated by the Grading of Recommendations Assessment, Development and Evaluation (GRADE) method.

**Results:**

A total of 41 studies with 3,269 patients were finally enrolled in this study. None of the included studies reported whether renal or cardiovascular disease progression events occurred. Compared with angiotensin-converting enzyme inhibitor (ACEI)/angiotensin II receptor blocker (ARB) alone, the combination with GBE was more beneficial in improving urinary albumin excretion rate (UAER) [mean difference (MD) = -22.99 μg/min, 95% confidence interval (CI): −27.66 to −18.31, *p* < 0.01], serum creatinine (SCr) [MD = −8.30 μmol/L, 95% CI: −11.55 to −5.05, *p* < 0.01], blood urea nitrogen (BUN) [MD = −0.77 mmol/L, 95% CI: −1.04 to −0.49, *p* < 0.01], 24-hour urinary total protein (24hUTP) [MD = −0.28 g/d, 95% CI: −0.35 to −0.22, *p* < 0.01], cystatin C (Cys-C) [MD = −0.30 mg/L, 95% CI: −0.43 to −0.17, *p* < 0.01], total cholesterol (TC) [MD = −0.69 mmol/L, 95% CI: −1.01 to −0.38, *p* < 0.01], triglyceride (TG) [MD = −0.40 mmol/L, 95% CI: −0.56 to −0.23, *p* < 0.01], low-density lipoprotein cholesterol (LDL-C) [MD = −0.97 mmol/L, 95% CI: −1.28 to −0.65, *p* < 0.01], fasting blood glucose (FBG) [MD = −0.30 mmol/L, 95% CI: −0.54 to −0.05, *p* = 0.02], hematocrit [MD = −4.58%, 95% CI: −5.25 to −3.90, *p* < 0.01] and fibrinogen [MD = −0.80 g/L, 95% CI: −1.12 to −0.47, *p* < 0.01]. No significant improvement was found in 2-hour postprandial glucose (2hPG), glycated hemoglobin (HbA1c), diastolic blood pressure (DBP) and systolic blood pressure (SBP). No significant difference was detected in adverse events.

**Conclusion:**

Combining GBE with ACEI/ARB may improve UAER, SCr, BUN, 24hUTP, Cys-C, TC, TG, LDL-C, hematocrit and fibrinogen in DKD patients. It also seems beneficial for oxidative stress and inflammation but has minimal impact on glucose and blood pressure. Combined GBE therapy is generally tolerated, but safety monitoring remains essential during its use. More long-term high-quality clinical studies and in-depth molecular research are still necessary to provide stronger evidence regarding the benefits and safety of GBE in DKD.

**Systematic Review Registration:**

https://www.crd.york.ac.uk/PROSPERO/display_record.php?RecordID=455792, identifier CRD42023455792

## 1 Introduction

Diabetic kidney disease (DKD) is currently the leading cause of end-stage renal disease (ESRD) in the world and an important factor in increasing the risk of cardiovascular disease and all-cause mortality (Hussain et al., 2021; CDC, 2023). About 30%–40% of diabetic patients will develop kidney disease and approximately 50% of them will eventually progress to ESRD (Kato and Natarajan, 2019; Cheng et al., 2021). Once DKD reaches the end stage, renal function severely deteriorates, and treatment options are limited to kidney replacement therapies (Rao et al., 2019). It is reported that the annual expenditure for severe DKD patients is as high as $25,000, and even more for those combined with cardiovascular or cerebrovascular diseases (Wan et al., 2020). Moreover, DKD patients still have a threefold higher risk of all-cause mortality and a 16-year loss in life expectancy compared with the general population (Naaman and Bakris, 2023). DKD not only seriously threatens patients’ health, but also imposes a huge economic burden on society (Rodriguez et al., 2021; Vanholder et al., 2021). How to effectively control DKD has become an important public health issue.

The treatment measures for DKD mainly include lifestyle intervention, controlling risk factors and reducing urinary protein, which aims to delay the disease progression and decrease cardio-renal adverse events and mortality (Selby and Taal, 2020; Chinese Diabetes Society, 2021). Renin-angiotensin-aldosterone system (RAAS) inhibitors, such as angiotensin-converting enzyme inhibitors (ACEIs) or angiotensin II receptor blockers (ARBs), are recommended as the first-line drugs for early and mid-stage DKD due to their role in controlling blood pressure, lowering urinary protein, delaying renal function deterioration and reducing cardiovascular events risks (Chinese Diabetes Society, 2021; American Diabetes Association, 2024). In recent years, some novel hypoglycemic drugs, including sodium-glucose cotransporter-2 inhibitors and glucagon-like peptide-1 receptor agonists, have been found to improve cardiorenal outcomes, providing more options for the treatment of DKD (Heerspink et al., 2020; Wheeler et al., 2021; Herrington et al., 2023). Even though substantial efforts have been invested, the residual risk for disease progression persists (Yamazaki et al., 2021). The morbidity and mortality rates remain high, and the clinical prognosis is not optimistic (Thomas, 2019; Ricciardi and Gnudi, 2021). There is an urgent need to explore more suitable complementary therapies for treating DKD.

*Ginkgo biloba* L., one of the ancient living trees in the world, is native to China and has existed since the Carboniferous era for 345 million years (Shahrajabian et al., 2022). Recently, extracts derived from its dried leaves have gained much recognition and are commonly used in many countries as medicines or dietary supplements (Eisvand et al., 2020; Das et al., 2022). *Ginkgo biloba* L. leaves extract (GBE) contains more than one hundred chemical constituents such as flavonoids, terpene lactones, organic acids, amino acids and trace elements (Chinese Pharmacopoeia Commission, 2020; Liu et al., 2022). The standardized GBE is prepared according to the German EGb761 quality specification and contains 24% flavonoid glycosides and 6% terpene lactones (2.8%–3.4% ginkgolides A, B and C, and 2.6%–3.2% bilobalide), with ginkgolic acid content not exceeding 5 parts per million (Chinese Pharmacopoeia Commission, 2020; Kulić et al., 2022). GBE has demonstrated significant efficacy in treating cardiovascular, cerebrovascular, and neurological diseases by reducing oxidative stress, inhibiting inflammatory factors, regulating blood lipids, and antagonizing platelet activating factors (Singh et al., 2019; Tao et al., 2022).

The diverse components of GBE make it a multi-pathway and multi-targeted therapeutic feature, consistent with the treatment principles for DKD. Many clinical studies have commenced to assess the potential effects of GBE in treating DKD (Zhao et al., 2015; Sun, 2016; Li et al., 2020). However, there is still a lack of convincing evidence to support its use due to the inconsistent results among studies. This study collects the latest evidence and conducts a systematic review with a rigorous method, evaluating the efficacy and safety of GBE as a supplement to ACEI/ARB in treating DKD, and providing guidance for clinical application.

## 2 Materials and methods

### 2.1 Study registration

This study followed the guidelines outlined in the Preferred Reporting Items for Systematic Review and Meta-Analysis (PRISMA) guidelines (Page et al., 2021). The PRISMA checklist can be found in Supplementary Material S1. The protocol was registered in International Prospective Registry of Systematic Reviews (PROSPERO) as CRD42023455792.

### 2.2 Database searches

The following databases were searched: PubMed, EMBASE, Cochrane Library, Web of Science (WOS), China National Knowledge Infrastructure (CNKI), Wan Fang Database, China Science and Technology Journal Database (VIP) and the China Biomedical Medicine database (CBM), from their inception until 21 July 2023. To identify clinical studies relevant to GBE and DKD, a comprehensive search strategy combining subject terms and text words was employed, mainly involving “Diabetic Nephropathies” “Diabetic Kidney Disease” “*G. biloba* extract” and “*Ginkgo* leaf extract”. Supplementary Material S2 shows the overall search strategies. To identify ongoing studies, the ClinicalTrials.gov database and CHiCTR were also searched. Furthermore, the references from related reviews and meta-analyses were screened to detect any possible missed literature during the online searches. The selection of literature was carried out based on pre-defined criteria.

### 2.3 Inclusion criteria

#### 2.3.1 Type of studies

Randomized controlled trials (RCTs) from any country or in any publication language.

#### 2.3.2 Type of participants

Adults meeting any recognized diagnostic criteria for DKD were included, with both type 1 and type 2 diabetes being eligible.

#### 2.3.3 Type of interventions and comparisons

Studies comparing GBE preparations combined with ACEI/ARB *versus* ACEI/ARB were included. No restrictions were placed on dosage form, dosage or duration. Other basic treatments in both groups were identical, which included dietary intervention, glycemic control, blood pressure management, lipid-lowering, maintaining electrolyte balance and other measures recommended by the guidelines.

#### 2.3.4 Type of outcome measures

The primary outcomes included kidney disease progression (starting renal replacement therapy, kidney disease-related death) and major adverse cardiovascular events (heart failure, myocardial infarction, cardiovascular death).

The secondary outcomes included: (1) renal function markers: urinary albumin excretion rate (UAER), serum creatinine (SCr), blood urea nitrogen (BUN), 24-h urinary total protein (24hUTP) and cystatin C (Cys-C); (2) glucose metabolism: fasting blood glucose (FBG), 2-h postprandial glucose (2hPG) and glycated hemoglobin (HbA1c); (3) lipid metabolism: total cholesterol (TC), triglyceride (TG) and low-density lipoprotein cholesterol (LDL-C); (4) blood pressure: diastolic blood pressure (DBP) and systolic blood pressure (SBP); (5) oxidative stress metrics: malondialdehyde (MDA), superoxide dismutase (SOD) and advanced oxidation protein product (AOPP); (6) inflammatory factors: high-sensitivity C-reactive protein (hs-CRP), interleukin-6 (IL-6) and tumor necrosis factor-α (TNF-α); (7) hemorheology indicators: hematocrit and fibrinogen.

The safety outcomes included any negative occurrences throughout the study, such as hypoglycemia, dry cough, elevated transaminases and allergies.

### 2.4 Exclusion criteria

#### 2.4.1 Type of studies

The studies involving the following conditions were not included: (1) Non-RCTs; (2) Animal or cell experiments; (3) Meeting abstracts that did not provide relevant data; (4) Studies for which full text is not available; (5) For any duplicate studies, the earliest published one was selected.

#### 2.4.2 Type of participants

Patients with the following disease states were excluded: (1) Patients with other kidney diseases, severe cardiovascular and cerebrovascular diseases, or malignant tumors; (2) Patients on dialysis were excluded; (3) Individuals experiencing acute metabolic disorders or infections.

#### 2.4.3 Type of interventions and comparisons

The following conditions were not considered: (1) Interventions involving non-pharmaceutical treatments, such as enemas, rehabilitation, nursing care or acupuncture; (2) Studies that used other herbal prescriptions or Chinese patent medicines that may affect the efficacy; (3) Literature with incomplete reporting on intervention characteristics, such as not reporting the dosage form, dose, frequency, name or duration of GBE or RAAS inhibitors.

#### 2.4.4 Type of outcome measures

Studies with obvious errors, incomplete data, questionable authenticity, or lack of required indicators were excluded.

### 2.5 Study selection and data extraction

The EndNote X9 software was utilized to import the search results as a bibliography and create a database. After removing duplicate studies, two researchers (ZZH and TSY) independently screened the literature by reading titles and abstracts to exclude irrelevant literature. Next, the full texts of the remaining articles were reviewed to determine their inclusion. Any discrepancies were resolved through discussion with a third researcher (LSY). The pre-designed extraction table was used to extract data from included studies. If some required information was not provided, we contacted the author via email. The following key information was extracted for all studies: study ID, study period, sample size, gender, average age, Mogensen stage, treatment duration, interventions, outcomes, baseline levels, and country, and was cross-checked. Under the guidance of the Consensus statement on the Phytochemical Characterization of Medicinal Plant extracts (ConPhyMP) (Heinrich et al., 2022), we extracted and evaluated the information on GBE formulations to ensure study rigor and reliability. This evaluation included formulation name, source, botanical plant name, plant part used, harvest time, specifications, composition and concentrations, quality control, and chemical analysis. For the inconsistent unit expression of UAER in different studies, conversion was performed according to the following formula (Chavan et al., 2011):

$$\text{UAER }\left(µg/\min\right)=\frac{\text{UAER}\;\left(\text{mg}/24\;\text{hours}\right)\times 1000}{24\times 60\;}$$

### 2.6 Risk of bias assessment

The Cochrane risk of bias tool was employed to evaluate the methodological quality of RCTs across seven domains: random sequence generation, allocation concealment, blinding of participants and personnel, blinding of outcome assessment, incomplete outcome data, selective reporting and other bias. The results were rated as “high risk”, “low risk” and “unclear risk” based on coverage for each domain, and were presented graphically. Two researchers (ZZH and TSY) independently conducted and cross-checked the assessments. If there was any disagreement, the third researcher (LSY) jointly discussed and determined the evaluation results.

### 2.7 Meta-analysis

Meta-analysis was conducted by Review Manager 5.4 (https://training.cochrane.org/revman) and Stata 16.0 software (https://www.stata.com). The mean difference (MD), standardized mean difference (SMD), relative risk (RR) and 95% confidence interval (CI) were used to represent the effect sizes for binary variables and continuous variables respectively. The selection between MD and SMD depended on whether the metric was measured by the same method. Heterogeneity was evaluated using the χ^2^ test and the I^2^ test, and the appropriate effect model was selected based on the results. Specifically, when *p* > 0.10, I^2^<50%, a fixed effect model was employed to determine the combined effect size; otherwise, a random effect model was applied. According to the Cochrane Handbook, meta-regression is not recommended when fewer than ten studies are included (Cochrane Groups, 2023). Therefore, we conducted meta-regression and subgroup analysis for indicators with more than ten included studies to reduce the false-positive rate and ensure the reliability of results. These were done to investigate the reasons for heterogeneity and to identify factors influencing the efficacy. Sensitivity analysis was carried out to evaluate the robustness of the results by eliminating one study at a time. If the combined effect size did not change significantly, it indicated the results were relatively stable. To gauge publication bias, both funnel plot visualization and Egger’s test analysis were employed, ensuring a comprehensive examination of potential biases. The trim and fill method was employed to identify and correct potential publication bias. It was conducted by iteratively estimating the number of missing studies and recalculating the overall results. If the estimated value of the effect size did not change significantly, it indicated that the impact of publication bias was small and the results were relatively robust (Duval and Tweedie, 2000).

### 2.8 Subgroup analysis and meta-regression

The analysis was conducted based on the following factors: average age (≤60 years old or >60 years old), GBE dosage form (injection or capsule), control preparation (ACEI or ARB) and sample size (<80 cases or ≥80 cases).

### 2.9 Evidence quality assessment

The Grading of Recommendations Assessment, Development and Evaluation (GRADE) method is an internationally unified method for grading the evidence quality and recommendation strength. Two researchers (ZZH and TSY) independently assessed each outcome using GRADE, and disagreements were resolved through discussion with the third researcher (LSY). The evidence quality was graded as follows: high, moderate, low, and very low. Evidence based on RCTs was initially considered high quality and downgraded if there were risks in the following five domains: risk for bias, inconsistency, indirectness, imprecision and publication bias. GRADE Profiler software (https://www.gradepro.org) was utilized for this process.

## 3 Results

### 3.1 Database search results

**FIGURE 1:**
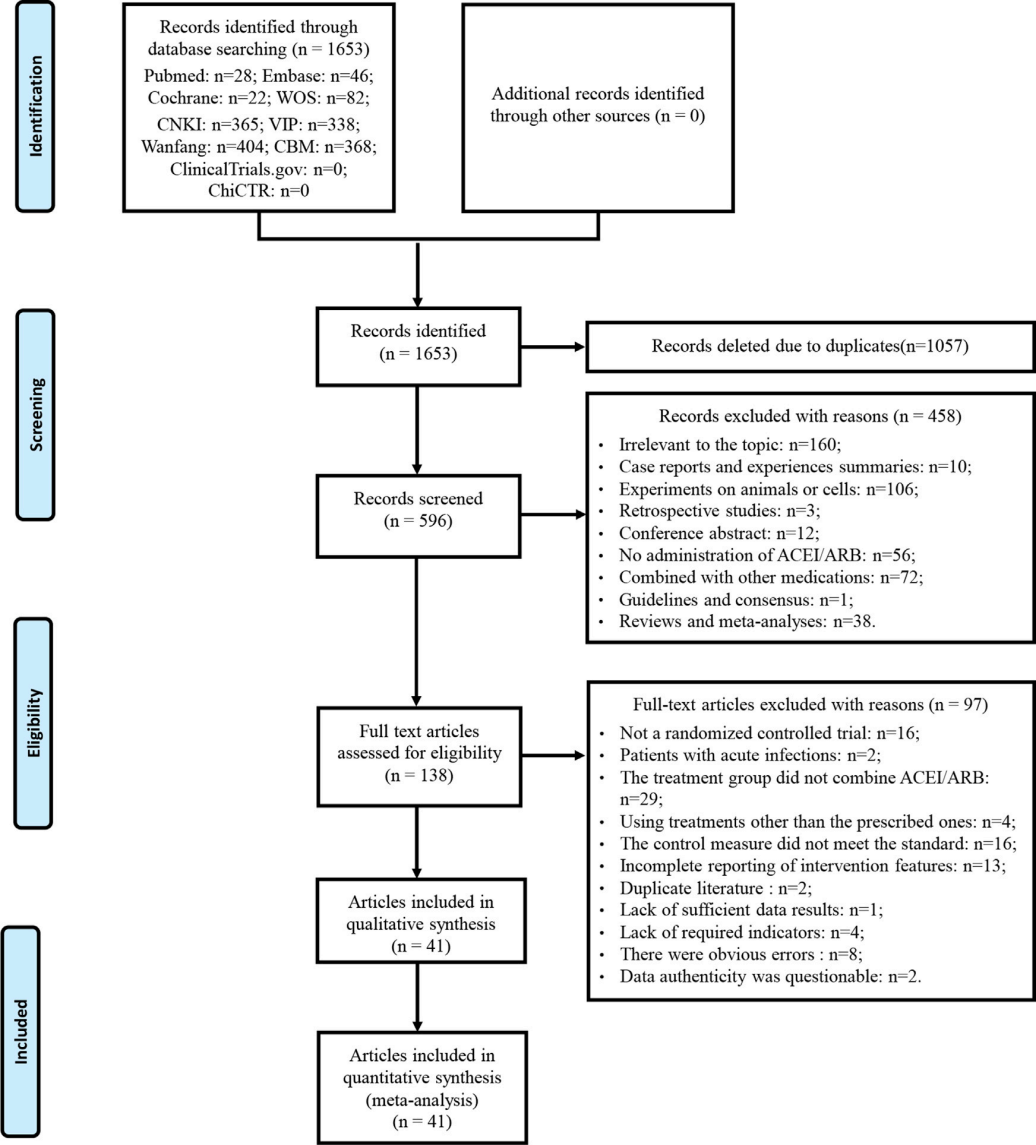
Flowchart of study selection and identification.

A total of 1,653 articles was obtained by database searches. After eliminating duplicates, 1,057 articles were excluded. Among the remaining 596 articles, 458 were deemed ineligible based on title and abstract screening. Following a thorough examination of the full text on the remaining 138 articles, 97 were excluded based on predefined criteria. No further studies meeting the criteria were discovered through the review of relevant reviews and meta-analyses. Eventually, 41 articles were enrolled. The literature that did not meet the criteria after reviewing the full texts, along with the reasons for exclusion, can be found in Supplementary Material S3. A detailed flowchart for the screening process is presented in Figure 1.

### 3.2 Characteristics of included studies

This study involved 41 RCTs, all conducted in China and published between 2006 and 2022 (Table 1). A total of 3,269 DKD patients were enrolled, with 1,658 in the treatment group and 1,611 in the control group, about 54% of whom were male. Each included study had sample sizes varying from 24 to 236 cases, with average age ranging from 41.77 to 66.70 years and treatment durations ranging from 2 to 24 weeks. GBE was administered intravenously in the form of injections in 39 studies (Fu and Jia, 2006; Yang et al., 2007; Han, 2008; Liang, 2008; Xiao, 2008; Li, 2009; Pan et al., 2009; Sun et al., 2009; Zhang H. S. et al., 2009; Zhang X. G. et al., 2009; Chen et al., 2010; Chu, 2010; Li, 2010; Shi, 2010; Wang et al., 2010; Wu et al., 2010; Xu et al., 2010; Xi, 2011; Zou and Zhang, 2011; Huang, 2012; Wen et al., 2012; Xu et al., 2012; Zhen and Feng, 2012; Li, 2013; Mao et al., 2013; Tang et al., 2013; Zhang et al., 2013; Jiang et al., 2014; Li, 2014; Zhang et al., 2014; Guo and Xu, 2015; Zhang, 2015; Hu et al., 2016; Huang et al., 2017; Shen, 2017; Wu et al., 2017; Cheng et al., 2018; Zhu et al., 2020; Xing, 2022) and orally in the form of capsules or tablets in two studies (Mao et al., 2010; Hu, 2019). For the control preparations, 16 studies used ACEI (Yang et al., 2007; Han, 2008; Liang, 2008; Li, 2009; Sun et al., 2009; Zhang H. S. et al., 2009; Shi, 2010; Wu et al., 2010; Xi, 2011; Tang et al., 2013; Jiang et al., 2014; Zhang et al., 2014; Hu et al., 2016; Shen, 2017; Wu et al., 2017; Hu, 2019) and 25 studies used ARB (Fu and Jia, 2006; Xiao, 2008; Pan et al., 2009; Zhang X. G. et al., 2009; Chen et al., 2010; Chu, 2010; Li, 2010; Mao et al., 2010; Wang et al., 2010; Xu et al., 2010; Zou and Zhang, 2011; Huang, 2012; Wen et al., 2012; Xu et al., 2012; Zhen and Feng, 2012; Li, 2013; Mao et al., 2013; Zhang et al., 2013; Li, 2014; Guo and Xu, 2015; Zhang, 2015; Huang et al., 2017; Cheng et al., 2018; Zhu et al., 2020; Xing, 2022). According to the ConPhyMP statement, the GBE formulations used in the included studies were all “type A″ extracts. These formulations were registered and approved by the National Medical Products Administration (NMPA) of China and manufactured by reputable, publicly listed pharmaceutical companies within the country. The preparations were produced in accordance with the quality control standards specified in the Chinese Pharmacopoeia and those promulgated by NMPA. Detailed evaluation information on the GBE formulations was provided in Supplementary Material S4.

**TABLE 1:** The characteristics of the included studies.

Study ID (year)	Study period	Sample size (randomized/analyzed) (T/C)	Gender (M/F) (T/C)	Average age (years) (T/C)	Mogensen stage	Treatment duration	Treatment group interventions	Control group interventions	Outcomes	Baseline difference	Country
Fu and Jia (2006)	2004.2–2005.2	32/32; 16/16	10/6; 9/7	61.47 ± 7.94	II	12 weeks	GBE 20 mL ivgtt qd + CG	Diet intervention + Hypoglycemic drugs + Losartan 50 mg po qd	②④⑥	NSD	China
Yang et al. (2007)	2003.10–2006.12	60/60; 35/25	19/16; 13/12	50–87; 46–89	III	4 weeks	GBE 20 mL ivgtt qd + CG	Diet intervention + Hypoglycemic drugs + Benazepril 10 mg po qd	②③④⑫⑬	NSD	China
Han (2008)	2006.2–2007.7	65/65; 35/30	35/30	50.6 ± 12.5	NR	6 weeks	GBE 20 mL ivgtt qd + CG	Diabetes health education + Diet intervention + Hypoglycemic drugs + Antihypertensive drugs + Benazepril 10–20 mg po qd	①②③⑨	NSD	China
Liang (2008)	2000.1–2007.1	60/60; 30/30	18/12; 17/13	50.8 ± 9.5; 49.6 ± 8.6	III	21 days	GBE 15 mL ivgtt qd + CG	Diabetes health education + Diet and exercise intervention + Hypoglycemic drugs + Perindopril 4 mg po qd	①②③④	NSD	China
Xiao (2008)	NR	40/40; 20/20	10/10; 12/8	NR	III	4 weeks	GBE 20 mL ivgtt qd + CG	Diet intervention + Hypoglycemic drugs + Valsartan 80 mg po qd	①②⑥⑳㉑	NSD	China
Zhang et al. (2009a)	2007.1–2009.1	65/65; 35/30	35/30	50.2 ± 8.5	III	14 days for a course of treatment, a total of 2 courses of treatment	GBE 20 mL ivgtt qd + CG	Diabetes health education + Diet and exercise intervention + Hypoglycemic drugs + Benazepril 10 mg po qd	①②③⑥	NSD	China
Pan et al. (2009)	2006.7–2008.10	48/48; 24/24	26/22	50 ± 8.95	III	4 weeks	GBE 10 mL ivgtt qd + CG	Diet and exercise intervention + Hypoglycemic drugs + Telmisartan 80 mg po qd	①②⑥⑳㉑	NSD	China
Li (2009)	2005.3–2008.3	160/160; 80/80	56/24; 46/34	52.6 ± 7.6; 51.8 ± 6.3	III	4 weeks	GBE 20 mL ivgtt qd + CG	Diet intervention + Hypoglycemic drugs + Antihypertensive drugs + Fosinopril 10 mg po qd	①②③⑨⑩㉑	NSD	China
Sun et al. (2009)	2007.10–2008.9	80/80; 40/40	22/18; 19/21	54.3 ± 9.1; 51.2 ± 9.2	NR	14 days for a course of treatment, a total of 2 courses of treatment	GBE 30 mL ivgtt qd + CG	Diet and exercise intervention + Hypoglycemic drugs + Antihypertensive drugs + Enalapril 10 mg po qd	①②③	NSD	China
Zhang et al. (2009b)	2007.1–2009.1	72/72; 36/36	38/34	62.3 ± 12.2	NR	4 weeks	GBE 21 mL ivgtt qd + CG	Diet and exercise intervention + Hypoglycemic drugs + Irbesartan 150 mg po qd	②③④	NSD	China
Shi (2010)	2006–2009	62/62; 31/31	18/13; 17/14	25–72/27–71	III	1 month	GBE 20 mL ivgtt qd + CG	Diet intervention + Hypoglycemic drugs + Hypolipidemic drugs + Antihypertensive drugs + Benazepril 10 mg po qd	①②③	NSD	China
Wu et al. (2010)	2008.1–2009.12	72/72; 36/36	39/33	36–72	NR	2 months	GBE 20 mL ivgtt qd + CG	Diet intervention + Hypoglycemic drugs + Enalapril 5 mg po bid	②③⑥⑧⑨⑩	NSD	China
Chen et al. (2010)	2007.1–2009.10	68/68; 34/34	15/19; 16/18	44 ± 6; 43 ± 8	III	3 weeks	GBE 20 mL ivgtt qd + CG	Diabetes health education + Hypoglycemic drugs + Antihypertensive drugs + Irbesartan 150 mg po qd	①②⑥	NSD	China
Mao et al. (2010)	2008.1–2008.12	39/36; 19/17	11/9; 8/11	50.5 ± 14.7; 51.3 ± 12.9	III	6 months	GBE 9.6 mg po tid + CG	Diabetes health education + Diet and exercise intervention + Hypoglycemic drugs + Irbesartan 150 mg po qd	①②③⑥⑧⑨⑩⑱⑲	NSD	China
Xu et al. (2010)	2004.6–2007.6	64/64; 32/32	20/12; 19/13	50 ± 7; 50 ± 6	NR	3 weeks	GBE 20 mL ivgtt qd + CG	Hypoglycemic drugs + Telmisartan 80 mg po qd	①②③⑥⑫⑬	NSD	China
Li (2010)	2006–2009	78/78; 39/39	22/17; 24/15	54.2 ± 14.3; 55.8 ± 14.7	III	30 days	GBE 20 mL ivgtt qd + CG	Diet and exercise intervention + Hypoglycemic drugs + Antihypertensive drugs + Irbesartan 0.15 g po qd	①②③⑥⑨⑩⑫⑬	NSD	China
Chu (2010)	2007.1–2008.12	112/112; 58/54	32/26; 28/26	56.1 ± 14.4; 56.2 ± 15.6	III	4 weeks for a course of treatment, a total of 2 courses of treatment	GBE 20 mL ivgtt qd + CG	Diabetes health education + Diet and exercise intervention + Hypoglycemic drugs + Hypolipidemic drugs + Antihypertensive drugs + Valsartan 80 mg po qd	①②③⑥⑨⑩⑫⑬	NSD	China
Wang et al. (2010)	2006.5–2009.4	90/90; 45/45	42/48	66.7 ± 13.8	II-III	4 weeks	GBE 20 mL ivgtt qd + CG	Diabetes health education + Diet and exercise intervention + Hypoglycemic drugs + Losartan 100 mg po qd	①②③⑥⑦⑨⑩	NSD	China
Xi (2011)	2004.7–2010.7	50/50; 25/25	31/19	45–70	III	6 weeks	GBE 20 mL ivgtt qd + CG	Diet intervention + Hypoglycemic drugs + Captopril 25 mg po tid	②④	NSD	China
Zou and Zhang (2011)	2007.6–2010.8	59/59; 30/29	17/13; 12/17	46.0 ± 13.6; 44.8 ± 10.1	III	14 days for a course of treatment, a total of 2 courses of treatment	GBE 20 mL ivgtt qd + CG	Diabetes health education + Diet and exercise intervention + Hypoglycemic drugs + Irbesartan 150 mg po qd	①②③⑥⑦⑨⑩⑫⑬	NSD	China
Wen et al. (2012)	2010.11–2011.7	46/46; 23/23	10/13; 12/11	43.5 ± 17.2; 45.4 ± 16.8	III	8 weeks	GBE 20 mL ivgtt qd + CG	Diabetes health education + Diet and exercise intervention + Hypoglycemic drugs + Irbesartan 150 mg po qd	①②③⑥⑩	NSD	China
Zhen and Feng (2012)	2009.6–2012.6	72/72; 36/36	21/15; 19/17	48–79; 49–78	NR	1 month	GBE 30 mL ivgtt qd + CG	Diet intervention + Glycemic control + Lipid lowering + Telmisartan 40 mg po bid	①②③④⑥⑧⑨⑩⑫⑬	NSD	China
Xu et al. (2012)	2007.6–2010.6	104/104; 52/52	33/19; 34/18	50.3 ± 6.7; 50.1 ± 5.5	NR	4 weeks	GBE 20 mL ivgtt qd + CG	Hypoglycemic drugs + Telmisartan 80–160 mg po qd	①②③⑥⑫⑬	NSD	China
Huang (2012)	2009.1–2011.12	196/196; 98/98	106/90	50–73	NR	4 weeks	GBE 20 mL ivgtt qd + CG	Diet intervention + Hypoglycemic drugs + Antihypertensive drugs + Losartan 100 mg po qd	①⑭⑮⑯	NSD	China
Li (2013)	2009.1–2009.12	97/97; 49/48	26/23; 25/23	46.13 ± 9.12; 45.73 ± 8.65	NR	2 weeks per course, with a 10-day break before starting the second course. A total of 2 courses were observed	GBE 20 mL ivgtt qd + CG	Glycemic control + Blood pressure management + Valsartan 80 mg po qd	①②③⑥⑫⑬	NSD	China
Zhang et al. (2013)	2011.1–2012.10	48/48; 24/24	13/11; 14/10	48.6 ± 11.8; 48.8 ± 12.4	I-III	4 weeks	GBE 20 mL ivgtt qd + CG	Diet and exercise intervention + Glycemic control + Blood pressure management + Lipid lowering + Erbesartan 150 mg po qd	①②③⑥⑧⑨⑩⑫⑬⑳㉑	NSD	China
Tang et al. (2013)	2011.6–2011.12	60/60; 31/29	16/15; 15/14	61.43 ± 2.06; 60.37 ± 1.97	NR	4 weeks	GBE 15 mL ivgtt qd + CG	Hypoglycemic drugs + Ramipril 2.5mg–5 mg po qd	⑥⑦	NSD	China
Mao et al. (2013)	2012.1–2012.12	40/40; 20/20	NR	NR	III	2 weeks	GBE 20 mL ivgtt qd + CG	Diet intervention + Hypoglycemic drugs + Valsartan 80 mg po qd	①②③⑥⑱	NSD	China
Zhang et al. (2014)	2010.1–2013.1	100/100; 50/50	53/47	45–79	III	1 month for a course of treatment, a total of 3 courses of treatment	GBE 20 mL ivgtt qd + CG	Diet intervention + Hypoglycemic drugs + Enalapril 10 mg po qd	②③⑥⑧⑨⑩	NSD	China
Li (2014)	2013.3–2013.12	24/24; 12/12	6/6; 7/5	55.2 ± 15.7; 56.3 ± 14.5	NR	3 weeks	GBE 25 mL ivgtt qd + CG	Health education + Diet and exercise intervention + Hypoglycemic drugs + Erbesartan 300 mg po qd	①②③⑨⑩	NSD	China
Jiang et al. (2014)	2009.1–2013.10	52/52; 26/26	27/25	60.3 ± 10.5	III	4 weeks	GBE 20 mL ivgtt qd + CG	Diet and exercise intervention + Hypoglycemic drugs + Enalapril 10 mg po bid	①②③④	NSD	China
Guo and Xu (2015)	2012.1–2013.6	60/60; 30/30	15/15; 17/13	41.77 ± 12.3; 43.4 ± 12.8	NR	2 weeks for a course of treatment, a total of 2 courses of treatment	GBE 20 mL ivgtt qd + CG	Diet and exercise intervention + Hypoglycemic drugs + Irbesartan 150 mg po qd	①③⑥⑩	NSD	China
Zhang (2015)	2011.5–2014.6	136/136; 68/68	35/33; 37/31	57.7 ± 4.2; 58.0 ± 4.0	NR	7 days for a course of treatment, a total of 4 courses of treatment	GBE 25 mL ivgtt qd + CG	Diet and exercise intervention + Blood pressure management + Irbesartan 150 mg po qd	①②④⑥⑦⑨⑩⑪	NSD	China
Hu et al. (2016)	2014.7–2015.3	50/50; 28/22	18/10; 14/8	37–64	III	2 weeks for a course of treatment, a total of 3 courses of treatment	GBE 20 mL ivgtt qd + CG	Diet and exercise intervention + Glycemic control + Blood pressure management + Lipid lowering + Imidapril 10 mg po qd	②③⑥⑨⑩⑪	NSD	China
Huang et al. (2017)	2015.5–2016.12	236/236; 118/118	63/55; 60/58	56.8 ± 3.4; 55.4 ± 3.7	NR	8 weeks	GBE 20 mL ivgtt qd + CG	Diet and exercise intervention + Hypoglycemic drugs + Hypolipidemic drugs + Antihypertensive drugs + Candesartan 12 mg po qd	②③④⑨⑳㉑	NSD	China
Wu et al. (2017)	2014.1–2015.1	60/60; 30/30	20/10; 18/12	57.6 ± 5.1; 56.9 ± 5.3	NR	1 month	GBE 10 mL ivgtt qd + CG	Benazepril 10 mg po qd	②④⑪⑰	NSD	China
Shen (2017)	2015.8–2016.4	60/60; 30/30	17/13; 21/9	66.49 ± 8.62; 65.51 ± 8.38	NR	3 weeks	GBE 20 mL ivgtt qd + CG	Hypoglycemic drugs + Benazepril 5 mg po qd	①②③⑤	NSD	China
Cheng et al. (2018)	2014.1–2017.12	125/125; 68/57	32/36; 26/31	45.3 ± 11.2; 48.2 ± 10.5	III	4 weeks	GBE 20 mL ivgtt qd + CG	Diet and exercise intervention + Hypoglycemic drugs + Telmisartan 80 mg po qd	①②⑫⑬⑭⑮⑯	NSD	China
Hu (2019)	2018.1–2019.1	74/74; 37/37	20/17; 21/16	60.85 ± 6.37; 60.96 ± 6.44	NR	6 weeks	GBE 0.5 g po tid + CG	Diet intervention + Hypoglycemic drugs + Enalapril 10 mg po qd	①②③④⑰⑱	NSD	China
Zhu et al. (2020)	2018.1–2018.10	60/60; 30/30	10/20; 12/18	60.3 ± 7.2; 60.5 ± 7.3	NR	10 days for a course of treatment, a total of 3 courses of treatment	GBE 25 mL ivgtt qd + CG	Diet intervention + Hypoglycemic drugs + Hypolipidemic drugs + Valsartan 80 mg po qd	②③④	NSD	China
Xing (2022)	2019.1–2020.12	196/196; 98/98	52/46; 51/47	56.39 ± 4.55; 56.37 ± 4.21	NR	15 days	GBE 20 mL ivgtt qd + CG	Diet intervention + Hypoglycemic drugs + Candesartan 8 mg po qd	①	NSD	China

Abbreviations: T, treatment group; C, control group; M, male; F, female; GBE, *Ginkgo biloba* L. leaves extract; CG, control group interventions; ivgtt, intravenously guttae; po, per os (oral administration); qd, quaque die (once a day); bid: bis in die (twice a day); tid, ter in die (three times a day); NR, not reported; NSD, no significant difference; Outcomes: ①UAER; ②SCr; ③BUN; ④24hUTP; ⑤Cys-C; ⑥FBG; ⑦2hPG; ⑧HbA1c; ⑨TC; ⑩TG; ⑪LDL-C; ⑫DBP; ⑬SBP; ⑭MDA; ⑮SOD; ⑯AOPP; ⑰hs-CRP; ⑱IL-6; ⑲TNF-α; ⑳hematocrit; ㉑fibrinogen.

### 3.3 Risk of bias assessment

**FIGURE 2:**
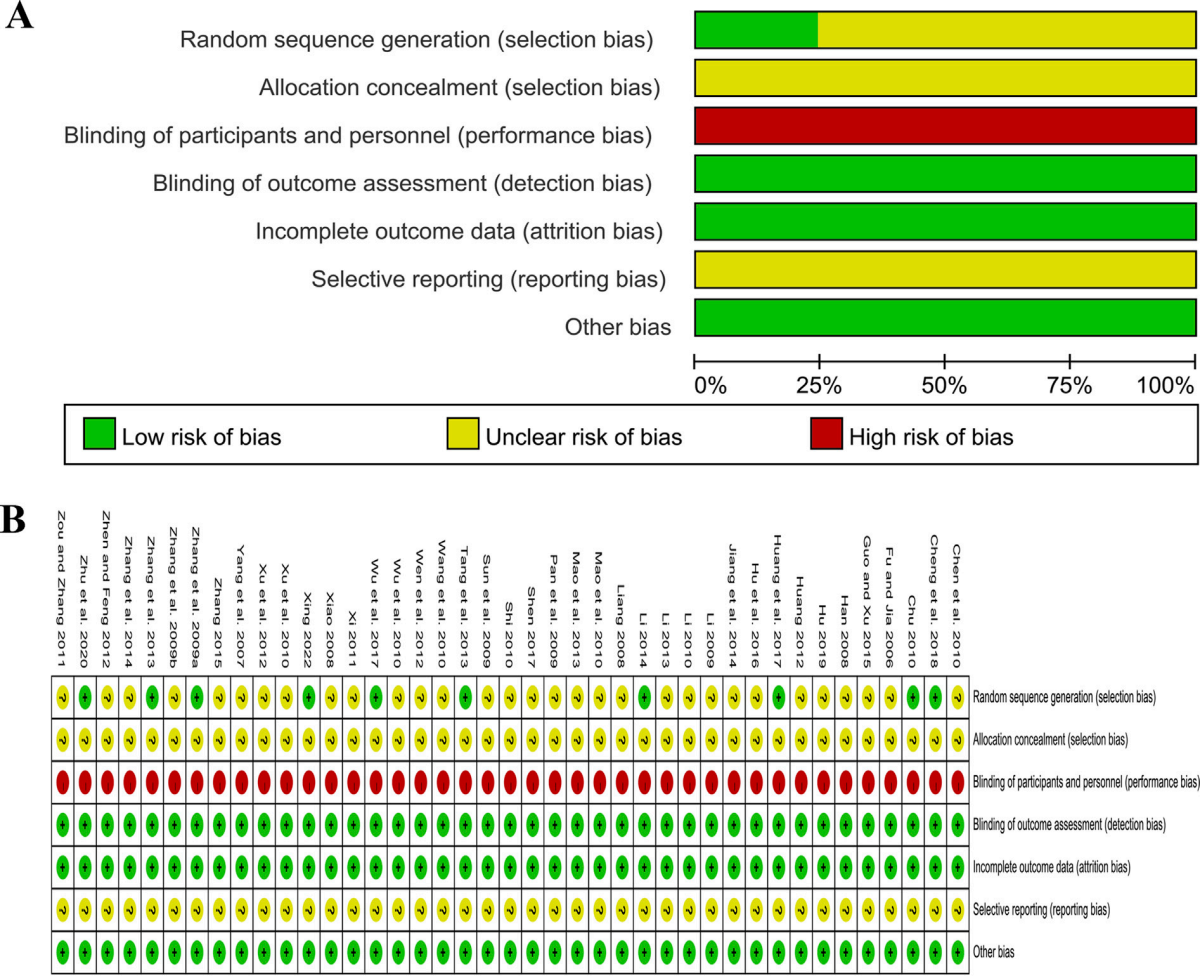
Risk of bias assessment for included studies: **(A)** Risk of bias graph; **(B)** Risk of bias summary.

Among the included studies, nine studies used a random number table (Zhang H. S. et al., 2009; Chu, 2010; Tang et al., 2013; Zhang et al., 2013; Huang et al., 2017; Wu et al., 2017; Cheng et al., 2018; Zhu et al., 2020; Xing, 2022) and one study utilized a computer-generated random sequence (Li, 2014), which were considered to be low risk. Other studies did not specify the methods used, and these were rated as unclear risk. Additionally, none of the studies provided details regarding allocation concealment, resulting in an assessment of unclear risk in this aspect. None of the studies used placebo to blind participants and researchers; therefore, all were rated as high risk. All studies assessed objective indicators, so although none reported whether the outcome assessors were blinded, the assessment of results was not affected, and all were rated as low risk for detection bias. In one study (Mao et al., 2010), both groups exhibited similar amounts of missing data, with similar reasons for their absence, and the others showed no cases of incomplete data, contributing to a low-risk rating for all included studies. Since no studies were registered, we were unable to obtain the study protocols to determine whether there was selective reporting, so all were rated as unclear risk. No additional significant biases were identified and all studies were considered to be low risk for other biases (Figure 2).

### 3.4 Primary outcomes

None of the included studies reported whether renal or cardiovascular disease progression events occurred during treatment or follow-up.

### 3.5 Secondary outcomes

#### 3.5.1 Effect on renal function markers

##### 3.5.1.1 UAER

**FIGURE 3:**
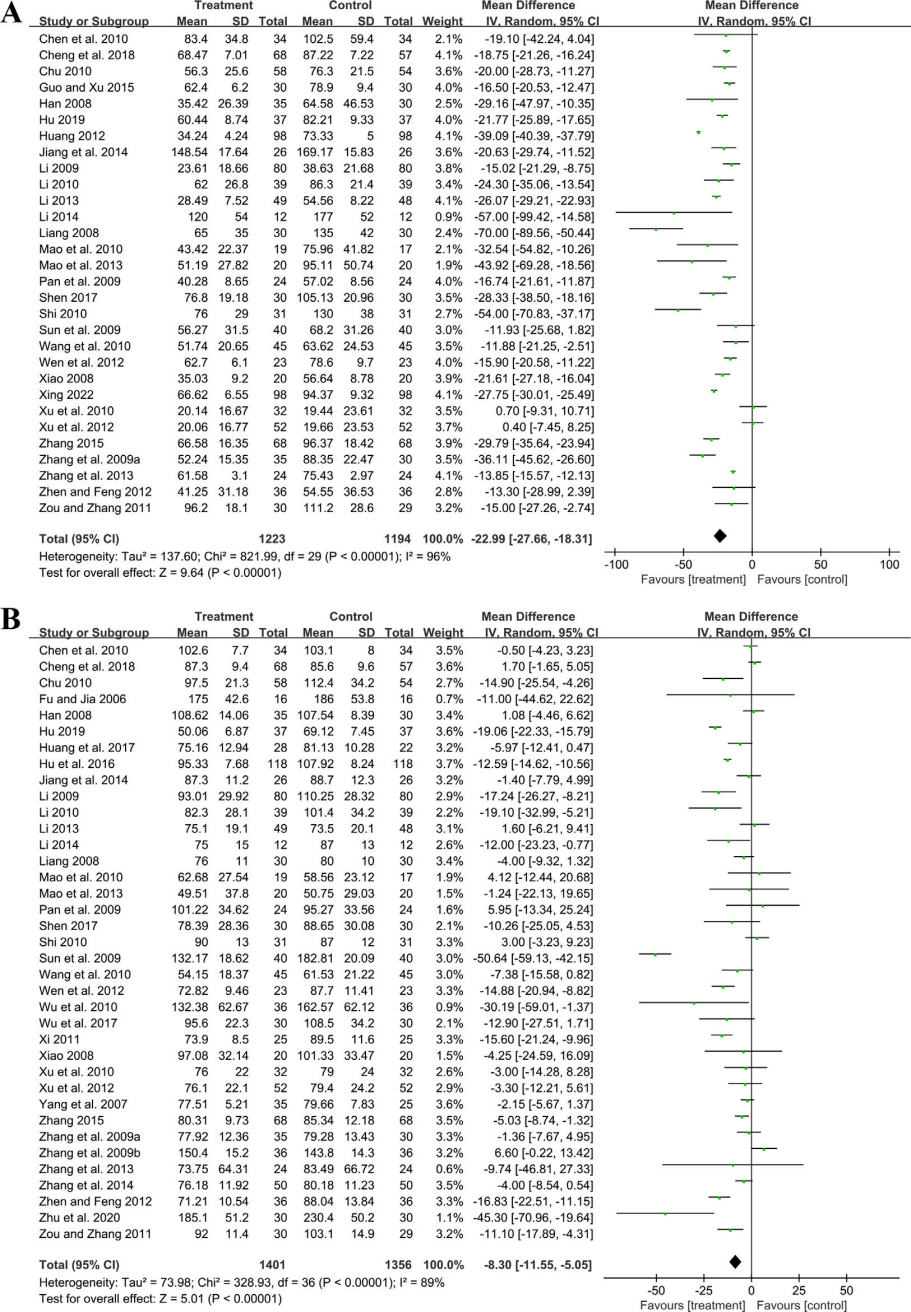
Forest plot of **(A)** UAER and **(B)** Scr.

**FIGURE 4:**
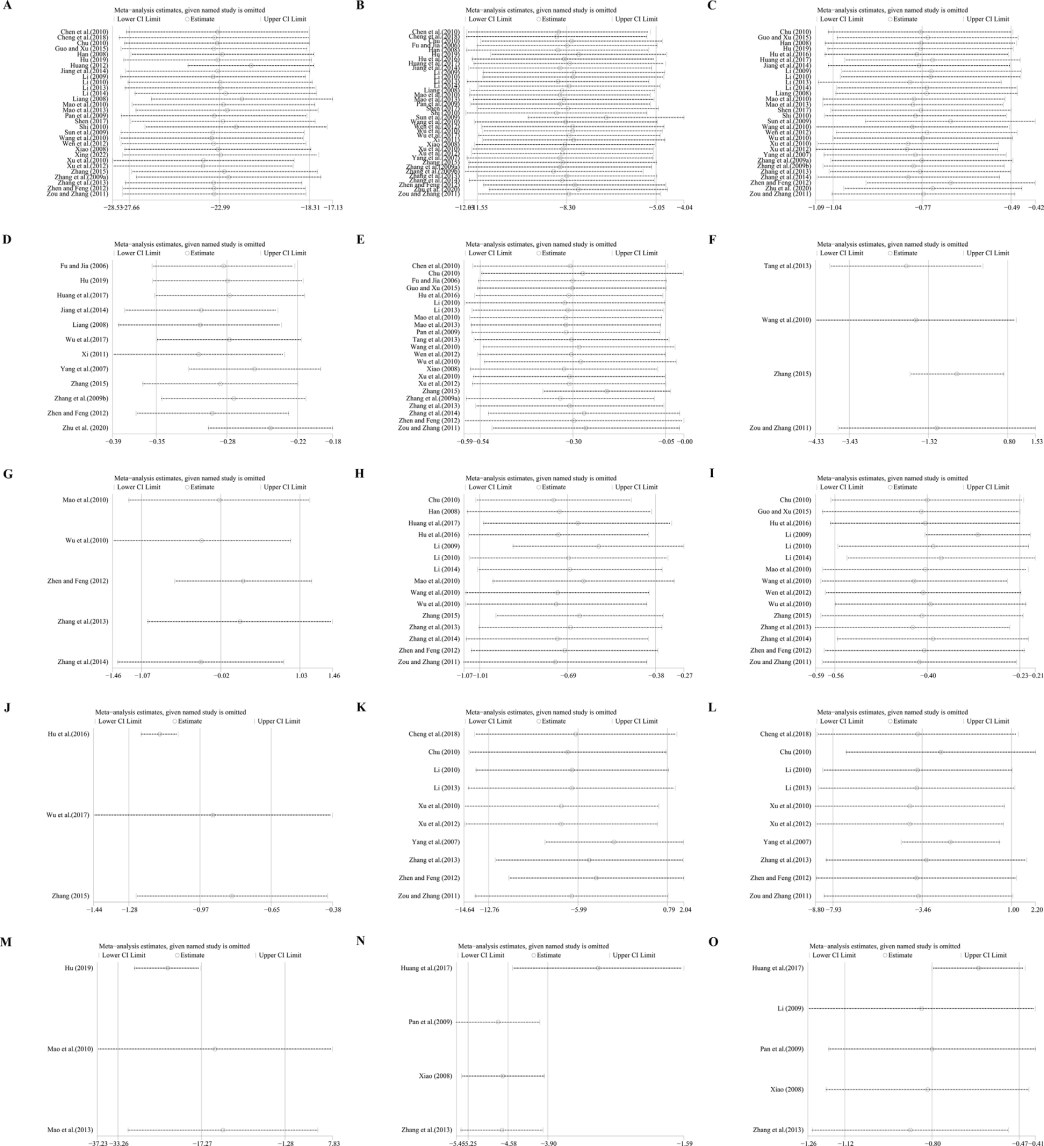
Sensitivity analysis: **(A)** UAER; **(B)** Scr; **(C)** BUN; **(D)** 24hUTP; **(E)** FBG; **(F)** 2hPG; **(G)** HbA1c; **(H)** TC; **(I)** TG; **(J)** LDL-C; **(K)** SBP; **(L)** DBP; **(M)** IL-6; **(N)** hematocrit; **(O)** fibrinogen.

Thirty studies involving 2,417 participants compared GBE plus ACEI/ARB with ACEI/ARB. A random effect model was utilized following the heterogeneity test (*p* < 0.01, I^2^ = 96%). The pooled result indicated that the addition of GBE to ACEI/ARB led to a statistically significant reduction in UAER compared to ACEI/ARB alone (MD = −22.99 μg/min, 95%CI: −27.66 to −18.31, *p* < 0.01) (Figure 3A). Meta-regression was conducted to investigate potential factors contributing to the observed heterogeneity. Generally, the average age (*p* = 0.56, Adj R^2^ = −2.87%), GBE dosage form (*p* = 0.80, Adj R^2^ = −5.21%), control preparation (*p* = 0.09, Adj R^2^ = 8.20%) and the sample size (*p* = 0.85, Adj R^2^ = −4.70%) could not explain the heterogeneity for UAER (Supplementary Material S5). Sensitivity analysis revealed consistent pooled effect sizes, indicating the robustness of the findings (Figure 4A; Supplementary Material S7).

##### 3.5.1.2 Scr

Thirty-seven trials involving 2,757 participants assessed the efficacy of GBE plus ACEI/ARB with ACEI/ARB. A random effect model was selected to synthesize original data following the heterogeneity test (*p* < 0.01, I^2^ = 89%). Comparing ACEI/ARB, the meta-analysis indicated that GBE in combination with ACEI/ARB could reduce Scr level (MD = −8.30 μmol/L, 95%CI: −11.55 to −5.05, *p* < 0.01) (Figure 3B). Meta-regression showed that average age (*p* = 0.32, Adj R^2^ = −1.16%), GBE dosage form (*p* = 0.82, Adj R^2^ = −3.01%), control preparation (*p* = 0.42, Adj R^2^ = −1.73%) and the sample size (*p* = 0.62, Adj R^2^ = −3.29%) could not explain the heterogeneity for Scr (Supplementary Material S5). Sensitivity analysis demonstrated consistent pooled effect sizes, indicating the robustness of the outcome (Figure 4B; Supplementary Material S7).

##### 3.5.1.3 BUN

**FIGURE 5:**
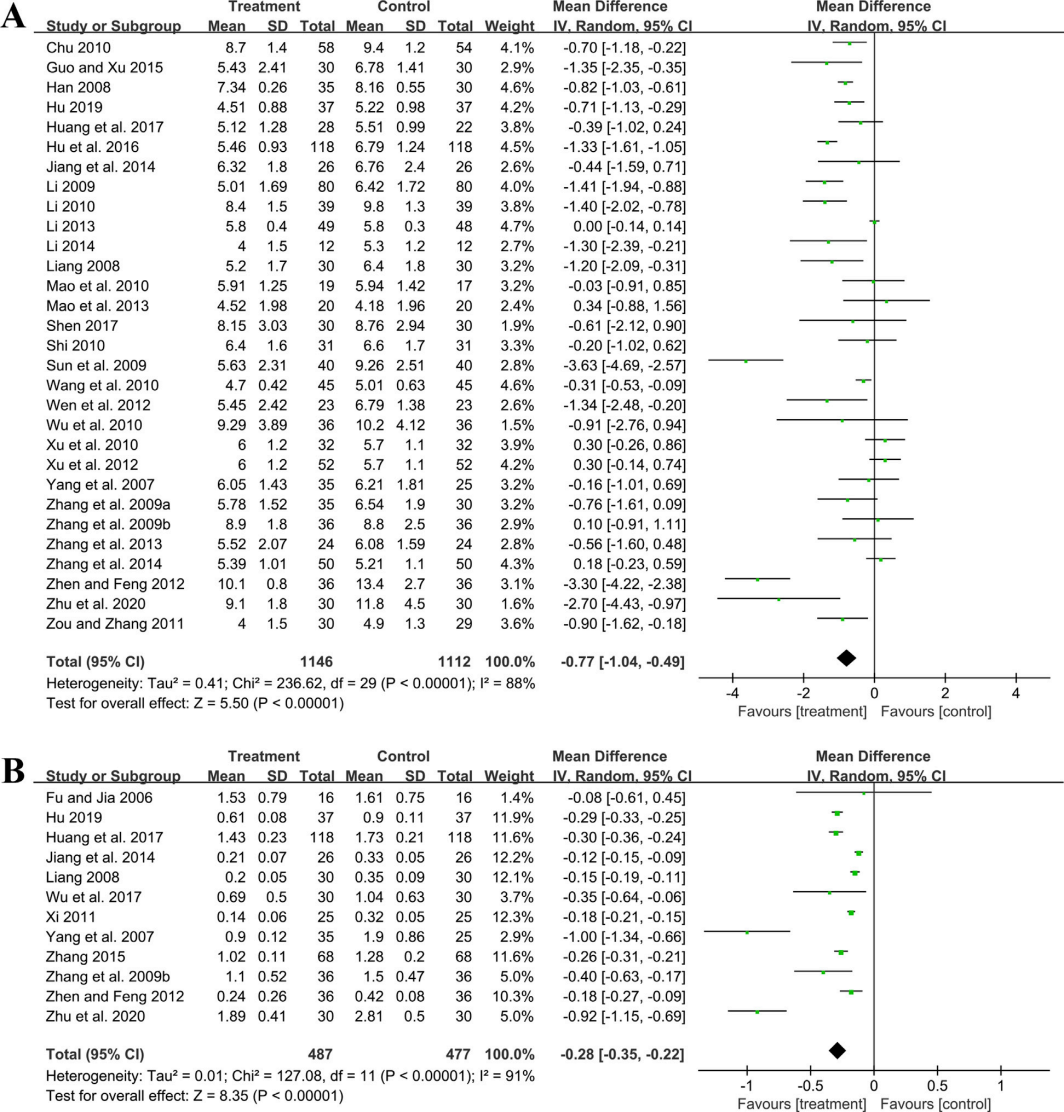
Forest plot of **(A)** BUN and **(B)** 24hUTP.

Thirty studies including 2,258 patients evaluated BUN levels. A random effect model was utilized following the heterogeneity test (*p* < 0.01, I^2^ = 88%). The result revealed a significant reduction in BUN with the combined use of GBE and ACEI/ARB (MD = −0.77 mmol/L, 95%CI: −1.04 to −0.49, *p* < 0.01) (Figure 5A). Meta-regression according to average age (*p* = 0.79, Adj R^2^ = −5.70%), GBE dosage form (*p* = 0.54, Adj R^2^ = −4.03%), control preparation (*p* = 0.87, Adj R^2^ = −4.82%) and sample size (*p* = 0.58, Adj R^2^ = −3.59%) showed no difference (Supplementary Material S5), which means none of these factors appeared to be the source of heterogeneity. Sensitivity analysis revealed a stable outcome, with similar pooled effect sizes (Figure 4C; Supplementary Material S7).

##### 3.5.1.4 24hUTP

Twelve studies including 964 patients evaluated 24hUTP outcomes. A random effect model was employed following the heterogeneity test (*p* < 0.01, I^2^ = 91%). Meta-analysis indicated that the combination of GBE with ACEI/ARB could significantly decrease 24hUTP compared to the control group (MD = −0.28 g/d, 95%CI: −0.35 to −0.22, *p* < 0.01) (Figure 5B). Meta-regression examining average age (*p* = 0.44, Adj R^2^ = −4.91%), GBE dosage form (*p* = 0.86, Adj R^2^ = −16.23%), control preparation (*p* = 0.76, Adj R^2^ = −11.15%) and sample size (*p* = 0.88, Adj R^2^ = −15.77%) did not identify factor to explain heterogeneity (Supplementary Material S5). Sensitivity analysis revealed a stable outcome, with similar pooled effect sizes (Figure 4D; Supplementary Material S7).

##### 3.5.1.5 Cys-C

One study (Shen, 2017) including 60 patients reported that, in comparison to ACEI/ARB alone, the addition of GBE led to a further reduction in Cys-C levels after 3 weeks of treatment (MD = −0.30 mg/L, 95%CI: −0.43 to −0.17, *p* < 0.01).

#### 3.5.2 Effect on glucose metabolism

##### 3.5.2.1 FBG

**FIGURE 6:**
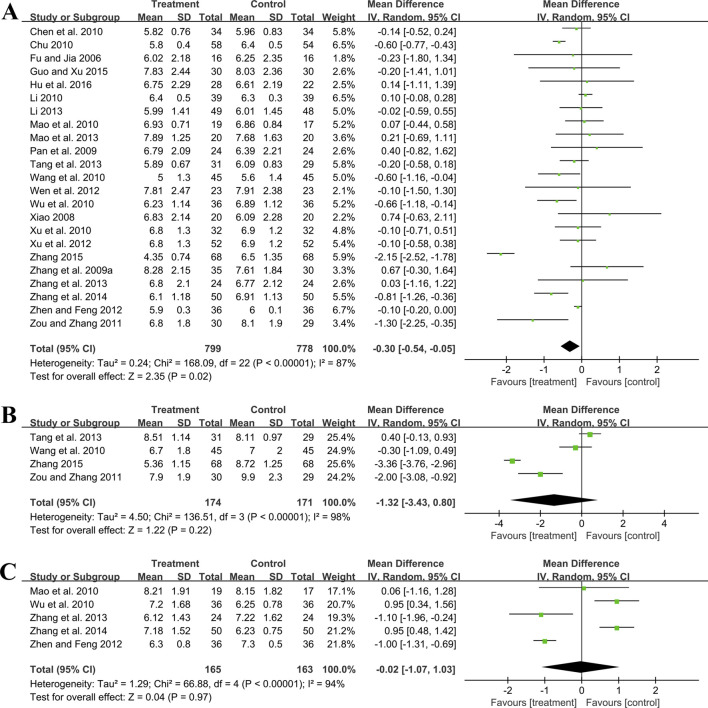
Forest plot of glucose metabolism outcomes: **(A)** FBG; **(B)** 2hPG; **(C)** HbA1c.

Twenty-three trials involving 1,577 patients assessed the efficacy of FBG. A random effect model was utilized following the heterogeneity test (*p* < 0.01, I^2^ = 87%). As shown by the pooled result, the combined therapy was more effective in reducing FBG (MD = −0.30 mmol/L, 95%CI: −0.54 to −0.05, *p* = 0.02) (Figure 6A). Meta-regression based on average age, GBE dosage form, control preparation and sample size was conducted to investigate potential factors contributing to the observed heterogeneity. Based on the meta-regression of sample size, scatters exhibited a linear pattern, with Tau^2^ decreasing from 0.240 to 0.142. This indicated that sample size may be the source of heterogeneity, with it accounting for 53.77% of the variability among study points (*p* < 0.01, Adj R^2^ = 53.77%) (Supplementary Material S5). Regression bubble plots showed that the difference in efficacy between groups increased with the study sample size (Supplementary Material S5). We further conducted subgroup analysis and found that when the sample size was ≤80 cases, there was no significant difference between the two groups (MD = −0.08 mmol/L, 95%CI: −0.21 to 0.06, *p* = 0.26). On the contrary, when the sample size was >80 cases, the combined treatment group had better efficacy in reducing FBG (MD = −0.73 mmol/L, 95%CI: −1.33 to −0.13, *p* < 0.01) (Supplementary Material S6). We considered the reason might be that the increased sample size reduced sampling error, improved statistical power and detected minor effects. Additionally, the average age (*p* = 0.18, Adj R^2^ = 8.40%), GBE dosage form (*p* = 0.56, Adj R^2^ = −3.62%) and control preparation (*p* = 0.96, Adj R^2^ = −6.17%) exhibited no difference (Supplementary Material S5). Sensitivity analysis revealed a relatively stable outcome, with consistent pooled effect sizes (Figure 4E; Supplementary Material S7).

##### 3.5.2.2 2hPG

Four trials involving 345 participants evaluated 2hPG outcomes. A random effect model was selected to synthesize original data following the heterogeneity test (*p* < 0.01, I^2^ = 98%). The pooled effect indicated no difference between groups (MD = −1.32 mmol/L, 95%CI: −3.43 to 0.80, *p* = 0.22) (Figure 6B). Sensitivity analysis showed consistent pooled effect sizes, indicating the robustness of the outcome (Figure 4F; Supplementary Material S7).

##### 3.5.2.3 HbA1c

Five studies including 328 patients evaluated HbA1c levels. A random effect model was utilized following the heterogeneity test (*p* < 0.01, I^2^ = 94%). The pooled effect indicated no difference between groups (MD = −0.02%, 95%CI: −1.07 to 1.03, *p* = 0.97) (Figure 6C). Sensitivity analysis showed that no matter which study was excluded, the effect size was not statistically significant, meaning the results were robust (Figure 4G; Supplementary Material S7).

#### 3.5.3 Effect on lipid metabolism

##### 3.5.3.1 TC

**FIGURE 7:**
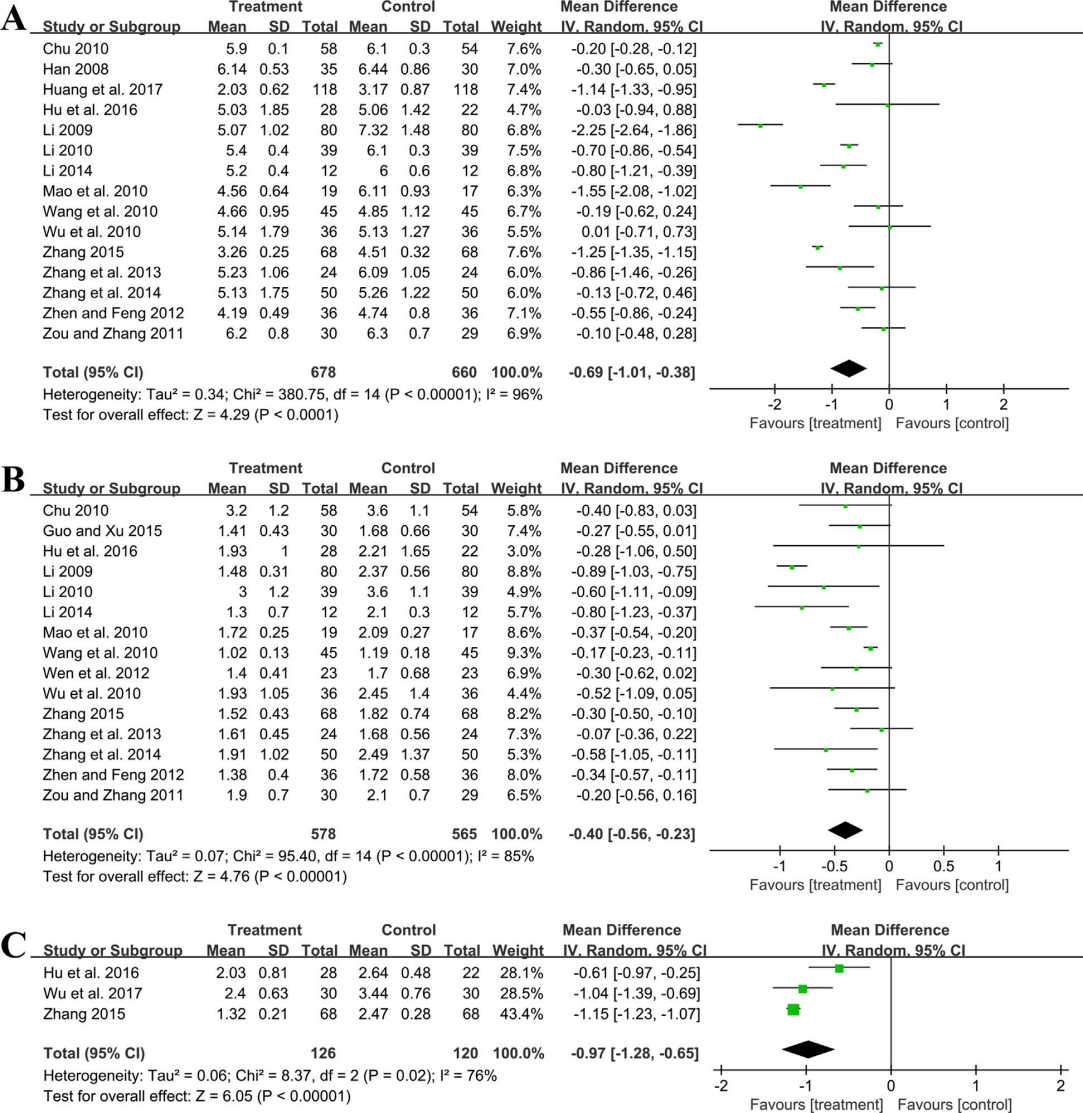
Forest plot of lipid metabolism outcomes: **(A)** TC; **(B)** TG; **(C)** LDL-C.

Fifteen studies including 1,338 patients reported TC levels. A random effect model was selected to synthesize original data following the heterogeneity test (*p* < 0.01, I^2^ = 96%). The pooled effect revealed that GBE combined with ACEI/ARB reduced TC more than ACEI/ARB (MD = −0.69 mmol/L, 95%CI: −1.01 to −0.38, *p* < 0.01) (Figure 7A). Meta-regression based on average age (*p* = 0.65, Adj R^2^ = −8.02%), GBE dosage form (*p* = 0.20, Adj R^2^ = 5.63%), control preparation (*p* = 0.74, Adj R^2^ = −8.43%) and sample size (*p* = 0.15, Adj R^2^ = 10.67%) did not find the factor to explain the heterogeneity (Supplementary Material S5). Sensitivity analysis suggests robustness in the result (Figure 4H; Supplementary Material S7).

##### 3.5.3.2 TG

Fifteen studies including 1,143 patients evaluated TG levels. A random effect model was used to synthesize original data following the heterogeneity test (*p* < 0.01, I^2^ = 85%). The pooled result indicated that the combination therapy was more beneficial (MD = −0.40 mmol/L, 95%CI: −0.56 to −0.23, *p* < 0.01) (Figure 7B). The meta-regression on control preparation revealed a decrease in Tau^2^ from 0.070 to 0.009, implying that control preparation might account for the heterogeneity and interpret 78.91% of the variation among studies (*p* < 0.01, Adj R^2^ = 78.91%) (Supplementary Material S5). Interestingly, the regression scatter plot indicated that the combination of GBE with ACEI was superior in lowering TG compared to its combination with ARB. Similar to the meta-regression, the subgroup analysis of control preparation exhibited a significant difference between subgroups (*p* < 0.01), with reduced heterogeneity within each group (I^2^ = 38% and 44%, respectively), suggesting that control preparation might be accountable for the heterogeneity (Supplementary Material S6). Additionally, the average age (*p* = 0.80, Adj R^2^ = −13.59%), GBE dosage form (*p* = 0.92, Adj R^2^ = −11.70%) and sample size (*p* = 0.15, Adj R^2^ = 18.56%) exhibited no difference (Supplementary Material S5). Sensitivity analysis indicated that the result remained consistent, suggesting its robustness (Figure 4I; Supplementary Material S7).

##### 3.5.3.3 LDL-C

Three trials involving 246 participants reported LDL-C levels. A random effect model was utilized following the heterogeneity test (*p* = 0.02, I^2^ = 76%). The pooled result indicated that the addition of GBE to ACEI/ARB led to a greater decrease in LDL-C (MD = −0.97 mmol/L, 95%CI: −1.28 to −0.65, *p* < 0.01) (Figure 7C). After excluding the study (Hu et al., 2016), the I^2^ changed from 76% to 0%, which means that this seems to be a source of heterogeneity. This study used lipid-lowering drugs to control blood lipids at a stable level during the lead-in period. Therefore, when GBE was added, the reduction in LDL-C was less than that of the other two studies, resulting in heterogeneity after the merger. Sensitivity analysis indicated that the effect size was statistically significant regardless of which study was excluded, suggesting the robustness of the result (Figure 4J; Supplementary Material S7).

#### 3.5.4 Effect on blood pressure

##### 3.5.4.1 SBP

**FIGURE 8:**
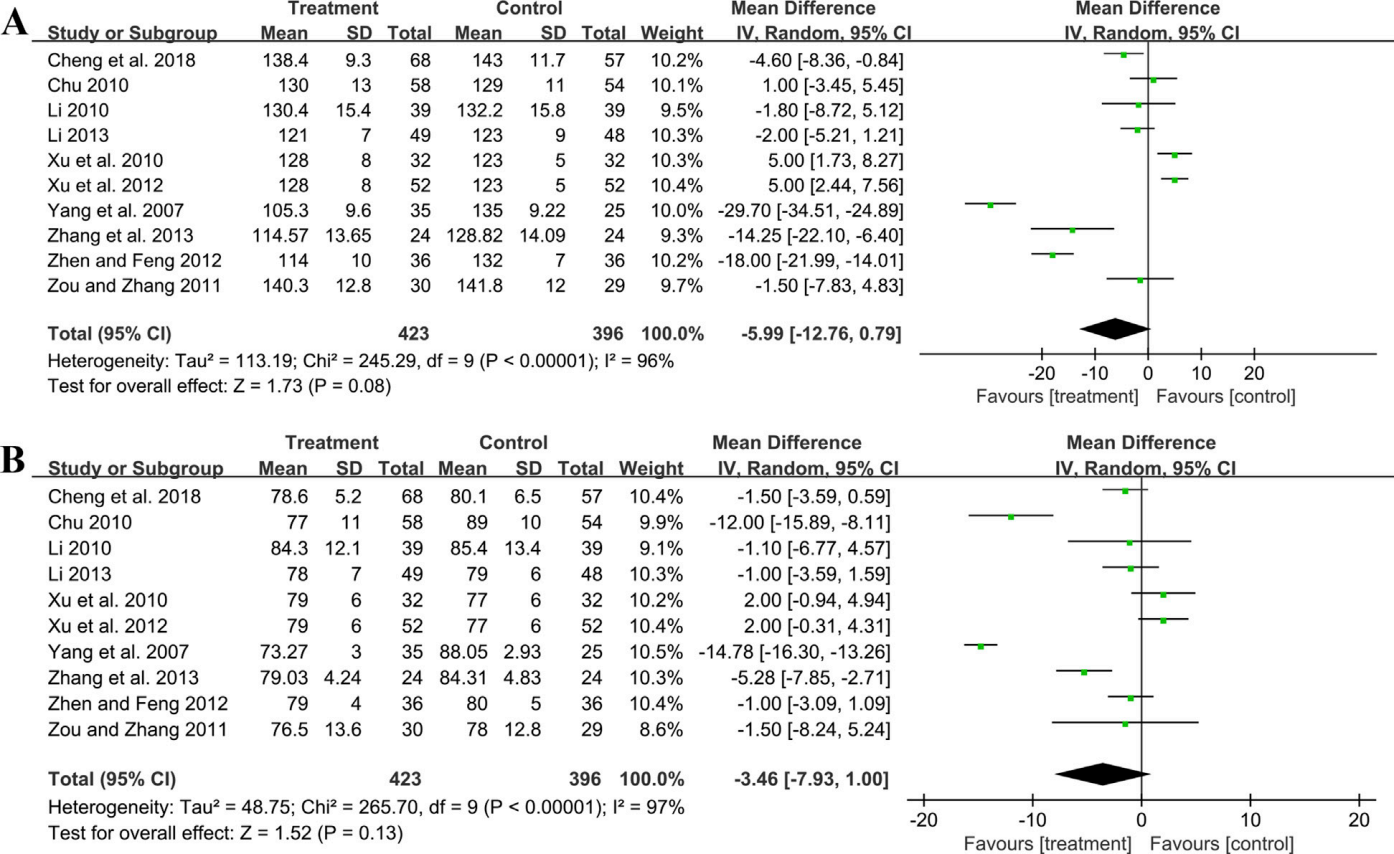
Forest plot of blood pressure outcomes: **(A)** SBP; **(B)** DBP.

Ten studies including 819 patients evaluated SBP. A random effect model was employed following the heterogeneity test (*p* < 0.01, I^2^ = 96%). The pooled result showed no statistical significance between two groups (MD = −5.99 mmHg, 95%CI: −12.76 to 0.79, *p* = 0.08) (Figure 8A). Subgroup analysis based on control preparation and sample size did not identify the factors that could account for the heterogeneity (Supplementary Material S6). Sensitivity analysis revealed a stable outcome, with similar pooled effect sizes (Figure 4K; Supplementary Material S7).

##### 3.5.4.2 DBP

Ten studies including 819 patients reported DBP. A random effect model was employed following the heterogeneity test (*p* < 0.01, I^2^ = 97%). The result indicated that the effect of GBE combined with ACEI/ARB on DBP was not statistically significant compared with ACEI/ARB (MD = −3.46 mmHg, 95%CI: −7.93 to 1.00, *p* = 0.13) (Figure 8B). Sensitivity analysis revealed a relatively stable outcome, with consistent pooled effect sizes (Figure 4L; Supplementary Material S7).

#### 3.5.5 Effect on oxidative stress

##### 3.5.5.1 MDA

**FIGURE 9:**
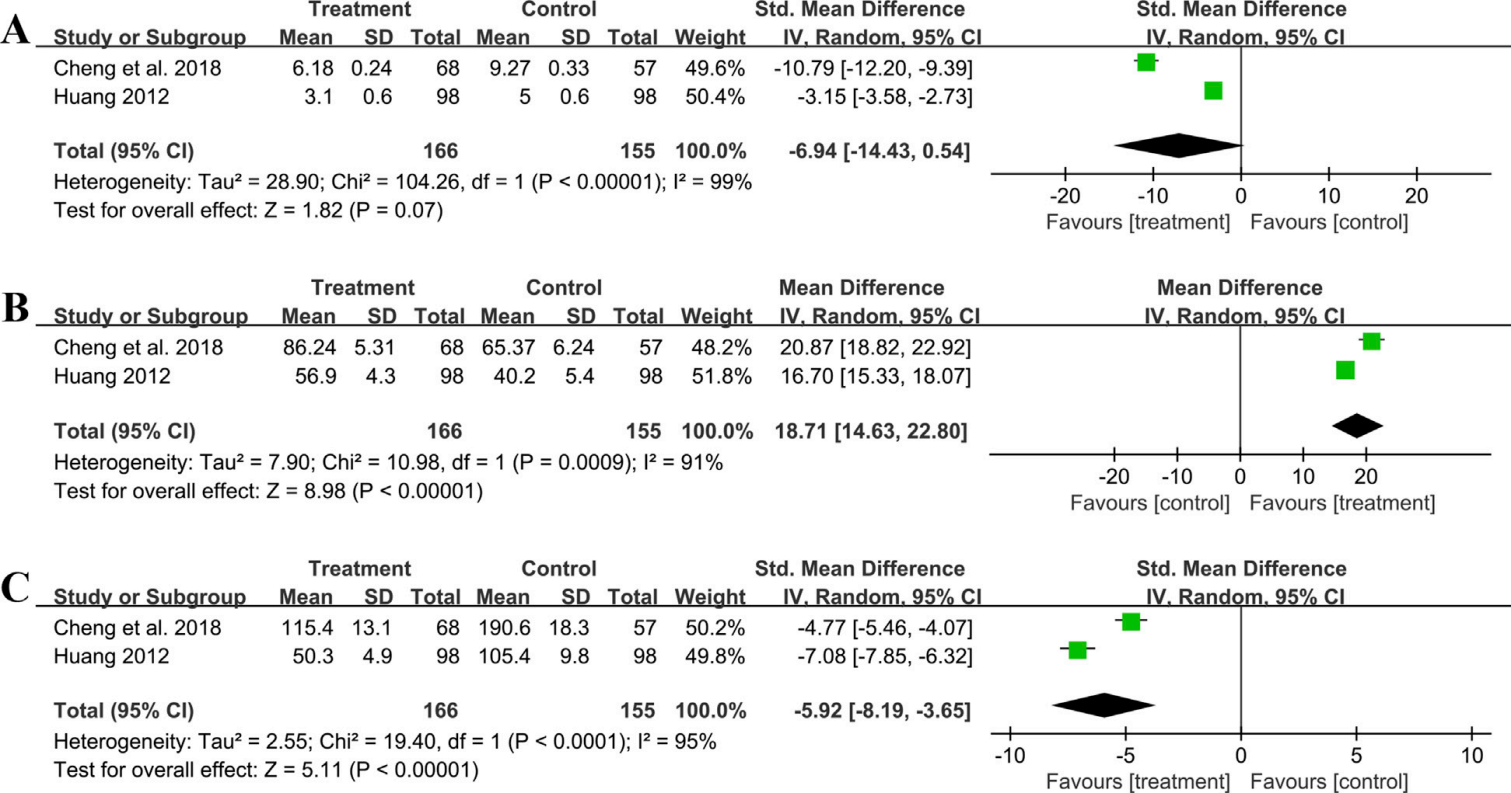
Forest plot of oxidative stress outcomes: **(A)** MDA; **(B)** SOD; **(C)** AOPP.

Two trials including 321 patients evaluated MDA levels. Both studies suggested that the addition of GBE to ACEI/ARB was better in lowering MDA. However, after merging these studies into the meta-analysis, we got opposite results (SMD = −6.94, 95%CI: −14.43 to 0.54, *p* = 0.07) (Figure 9A). This was due to the use of a random effect model to account for high heterogeneity (*p* < 0.01, I^2^ = 99%). Since the result of one study (Cheng et al., 2018) was much better than another (Huang, 2012), there was a lack of overlap in the effect size intervals between them, resulting in high heterogeneity. On the contrary, if we used a fixed effect model, we can get a positive result. Therefore, the effect of GBE combined with ACEI/ARB on MDA remains uncertain, and further rigorous research is needed to explore this.

##### 3.5.5.2 SOD

Two studies involving 321 patients provided data on SOD. According to the heterogeneity test (*p* < 0.01, I^2^ = 91%), the random effect model was applied. The pooled result showed that combination treatment significantly improved SOD than ACEI/ARB (MD = 18.71 U/mL, 95%CI: 14.63 to 22.80, *p* < 0.01) (Figure 9B). The result remained similar when changing to a fixed effect model, indicating its robustness (Supplementary Material S7).

##### 3.5.5.3 AOPP

Two studies including 321 patients evaluated AOPP. According to the heterogeneity test (*p* < 0.01, I^2^ = 95%), the random effect model was used. The pooled result showed that GBE combined with ACEI/ARB could significantly reduce the AOPP level compared to the control group (SMD = −5.92, 95%CI: −8.19 to −3.65, *p* < 0.01) (Figure 9C). The heterogeneity could be associated with variations in AOPP baseline levels between study points, as well as the potential measurement bias arising from different detection methods. The result remained similar when changing to a fixed effect model, indicating its robustness (Supplementary Material S7).

#### 3.5.6 Effect on inflammatory factors

##### 3.5.6.1 hs-CRP

**FIGURE 10:**
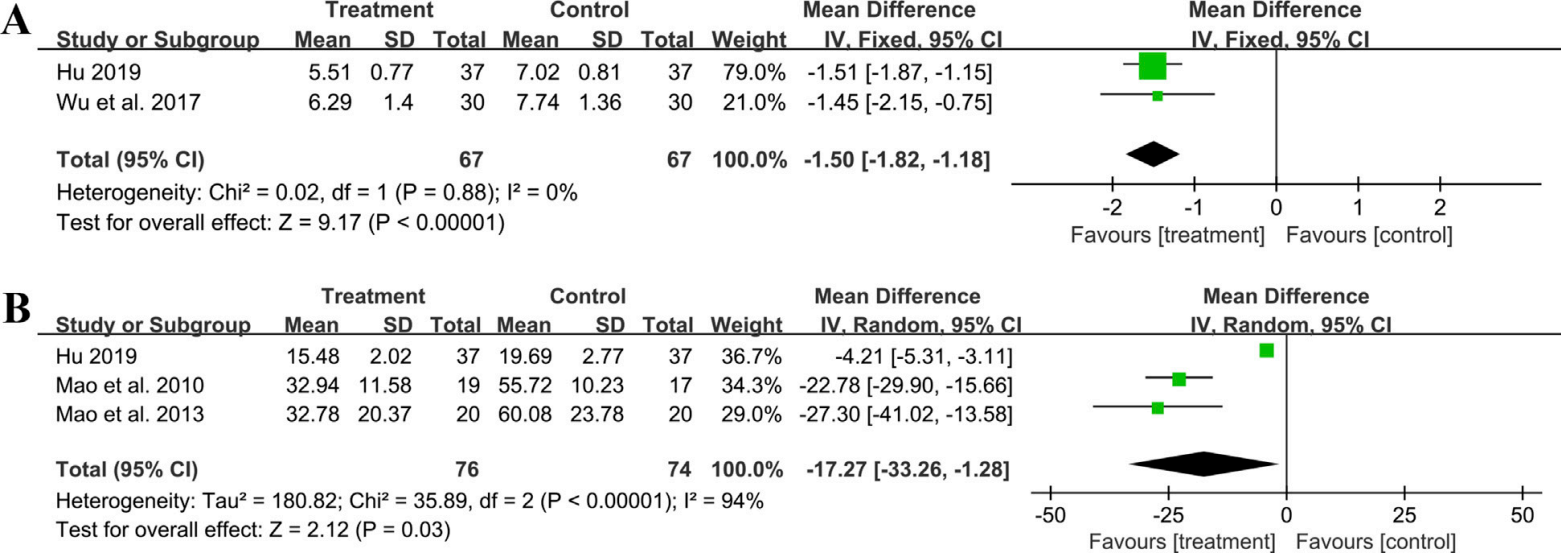
Forest plot of inflammatory factors: **(A)** hs-CRP; **(B)** IL-6.

Two studies including 134 patients reported hs-CRP outcomes. A fixed effect model was used to synthesize the original data following the heterogeneity test (*p* = 0.88, I^2^ = 0%). The pooled effect indicated that the combination of GBE with ACEI/ARB was superior to ACEI/ARB alone in reducing hs-CRP levels (MD = −1.50 mg/L, 95%CI: −1.82 to −1.18, *p* < 0.01) (Figure 10A). The result remained statistically significant after transitioning the effect model, implying its robustness (Supplementary Material S7).

##### 3.5.6.2 IL-6

Three studies including 150 patients evaluated IL-6 levels. According to the heterogeneity test (*p* < 0.01, I^2^ = 94%), the random effect model was used. The meta-analysis showed that combination treatment was better than ACEI/ARB in reducing IL-6 (MD = −17.27 ng/L, 95%CI: −33.26 to −1.28, *p* = 0.03) (Figure 10B). Sensitivity analysis found that after excluding Mao et al., 2010 or Mao et al., 2013, the combined findings exhibited a reversal and had no statistical significance, meaning the results were not robust (Figure 4M; Supplementary Material S7). This might be attributed to the literature (Hu, 2019), which showed a weaker efficacy. It carries a large weight in the pooled results, causing the upper confidence interval tending toward the null line. After excluding Mao et al., 2010 or Mao et al., 2013, the result’s weight of Hu, 2019 becomes even more prominent, leading to an exceeding of the null line. Additionally, the use of a random effect model makes the results more conservative. Given that all three studies have suggested positive results, we are optimistic about the effect of combination treatment on IL-6, but more rigorous trials are still needed to verify this.

##### 3.5.6.3 TNF-α

One study (Mao et al., 2010) including 36 patients reported that the combination treatment resulted in a greater reduction in TNF-α levels compared to ACEI/ARB alone following 6 months of treatment (MD = −25.95 ng/L, 95%CI: −34.64 to −17.26, *p* < 0.01).

#### 3.5.7 Effect on hemorheology indicators

##### 3.5.7.1 Hematocrit

**FIGURE 11:**
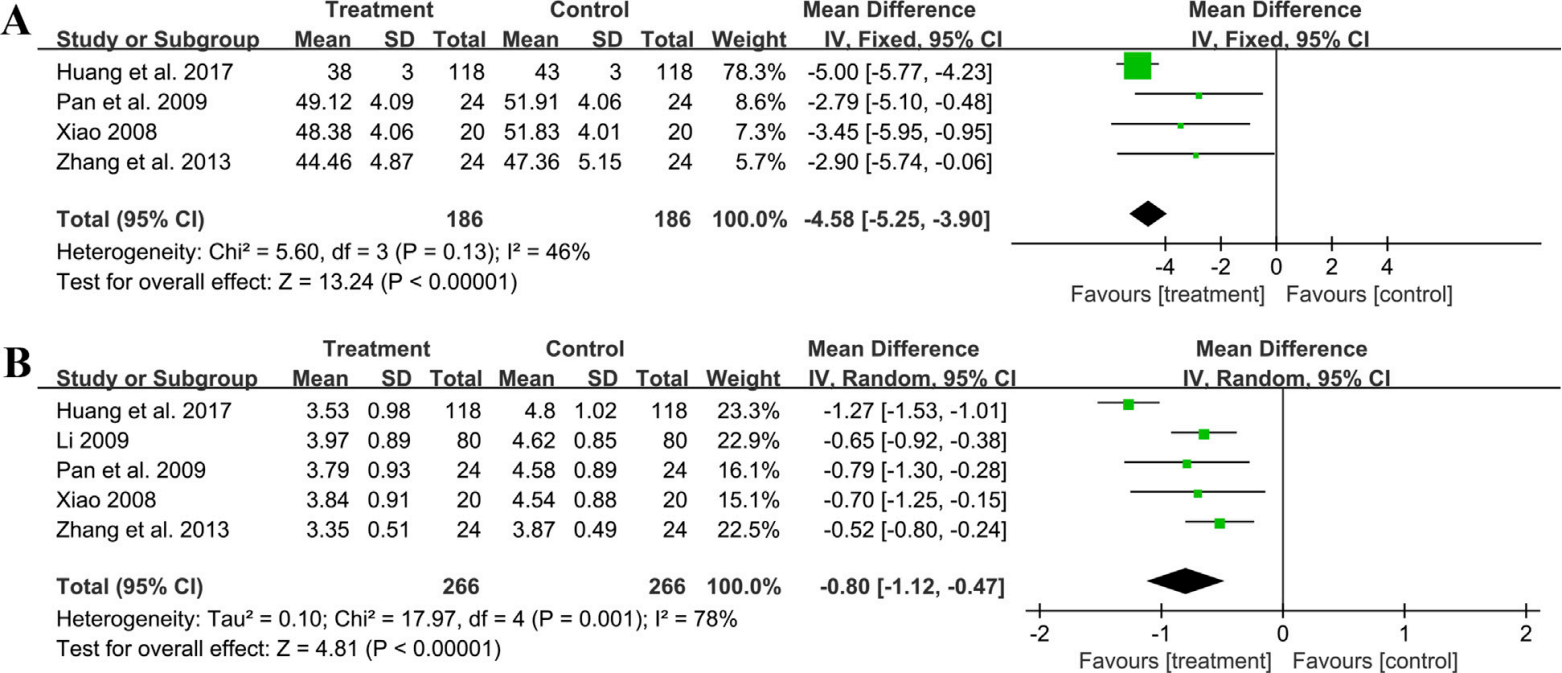
Forest plot of hemorheology indicators: **(A)** hematocrit; **(B)** fibrinogen.

Four trials involving 372 individuals assessed the hematocrit outcome. A fixed effect model was used to synthesize original data following the heterogeneity test (*p* = 0.13, I^2^ = 46%). The pooled result indicated a greater reduction on hematocrit for combined therapy compared to ACEI/ARB alone (MD = −4.58%, 95%CI: −5.25 to −3.90, *p* < 0.01) (Figure 11A). Sensitivity analysis revealed a relatively stable outcome, with consistent pooled effect sizes (Figure 4N, Supplementary Material S7).

##### 3.5.7.2 Fibrinogen

Five studies including 532 patients reported the fibrinogen level. A random effect model was used to synthesize original data following the heterogeneity test (*p* < 0.01, I^2^ = 78%). The result suggested that combination treatment was superior to ACEI/ARB alone in lowering fibrinogen levels (MD = −0.80 g/L, 95%CI: −1.12 to −0.47, *p* < 0.01) (Figure 11B). After excluding the study (Huang et al., 2017), the I^2^ changed from 78% to 0%, implying that this literature may be a source of heterogeneity. This study had a longer treatment duration than the other studies, demonstrating a better efficacy. And no matter which study was excluded, the pooled effect size remained statistically significant, suggesting a relatively stable result (Figure 4O, Supplementary Material S7).

### 3.6 Safety outcomes

**FIGURE 12:**
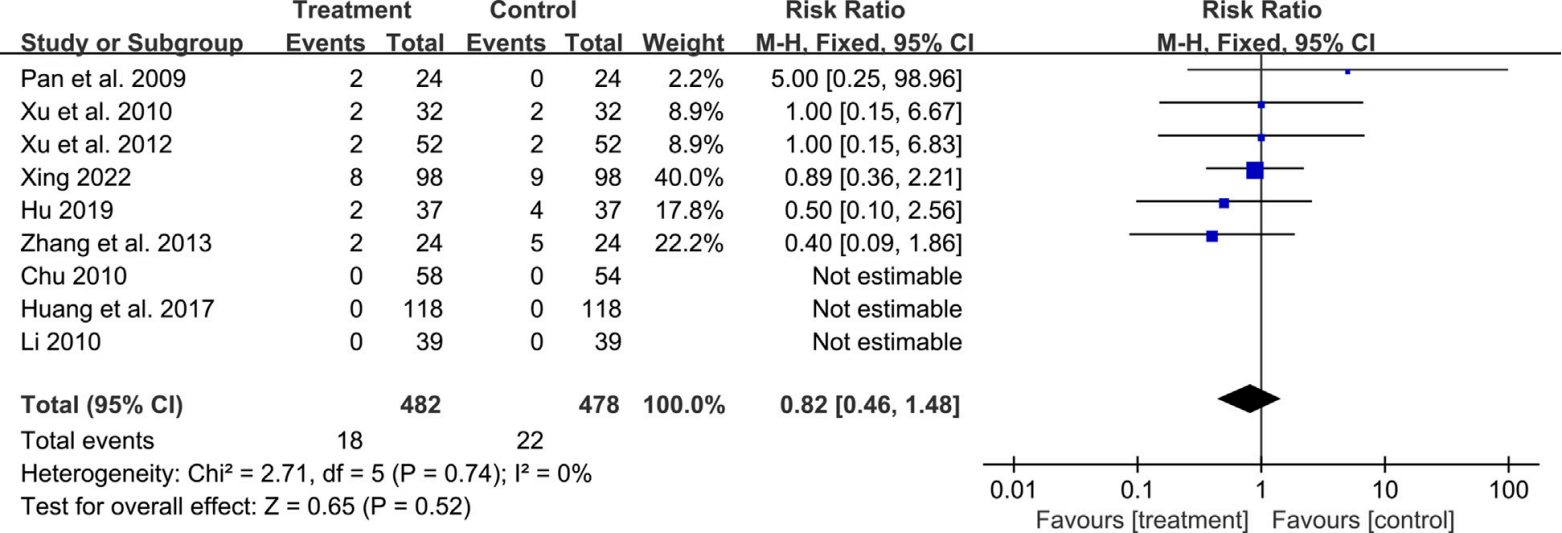
Forest plot of adverse events.

A total of ten studies reported adverse events. Among them, nine studies reported the number of adverse events occurring in each group (Pan et al., 2009; Chu, 2010; Li, 2010; Xu et al., 2010; Xu et al., 2012; Zhang et al., 2013; Huang et al., 2017; Hu, 2019; Xing, 2022). The occurrence of adverse events in the two groups was 18/482 and 22/478 respectively, and meta-analysis indicated no significant difference (RR = 0.82, 95%CI: 0.46 to 1.48, *p* = 0.52) (Figure 12). Another study (Xiao, 2008) reported the sum adverse events of two groups, with three cases of mild cough and no other adverse events observed. As shown in Table 2, the most common adverse event was dizziness, and other adverse events such as skin rash, nausea and vomiting, dry cough, headache and lower limb soreness were also reported. In addition, a study (Xing, 2022) reported that *one* dyspnea occurred in the treatment group and *one* laryngeal edema in the control group, which were both rare and severe adverse events. No deaths were reported in these studies.

**TABLE 2:** Summary of adverse events.

Adverse events	GBE combined with ACEI/ARB group (482 patients)	ACEI/ARB group (478 patients)
Headache	2 (0.41%)	1 (0.21%)
Dry cough	2 (0.41%)	4 (0.84%)
Dizziness	5 (1.04%)	5 (1.05%)
Nausea and vomiting	4 (0.83%)	4 (0.84%)
Skin rash	4 (0.83%)	5 (1.05%)
Lower limb soreness	0 (0.00%)	2 (0.42%)
Laryngeal edema	0 (0.00%)	1 (0.21%)
Dyspnea	1 (0.21%)	0 (0.00%)

### 3.7 Subgroup analysis

Subgroup analysis was performed on UAER, Scr, BUN, 24hUTP, FBG, TC, TG, SBP and DBP to explore the influence of different average age, GBE dosage form, control preparation and sample size on efficacy. Pooled effect sizes for subgroups are summarized in Supplementary Material S6. Subgroup analysis implied that the combination treatment had a significant effect on UAER, Scr and 24hUTP levels in patients with different ages. As for the BUN, TC and TG, it could reduce their levels in patients under 60 years old, but may not be effective in elderly patients over 60. Conversely, when it came to FBG, the situation was reversed. In addition, GBE, whether administered through injection or capsules, demonstrated efficacy in reducing UAER, 24hUTP, TC and TG levels. However, when administered orally in capsule form, GBE may not lower Scr, BUN and FPG.

The combined use of GBE with either ACEI or ARB led to reductions in UAER, Scr, BUN, 24hUTP and TG levels. GBE in combination with ARB effectively lowered FBG and TC levels, while in combination with ACEI, it did not seem to have the same effect. The effect of combination treatment on UAER, Scr, BUN, 24hUTP, TC, TG, SBP and DBP was not affected by the sample size. However, the effect on FBG did not reach statistical significance when the sample size was less than 80, but it became statistically significant when the sample size exceeded 80.

### 3.8 Publication bias

**FIGURE 13:**
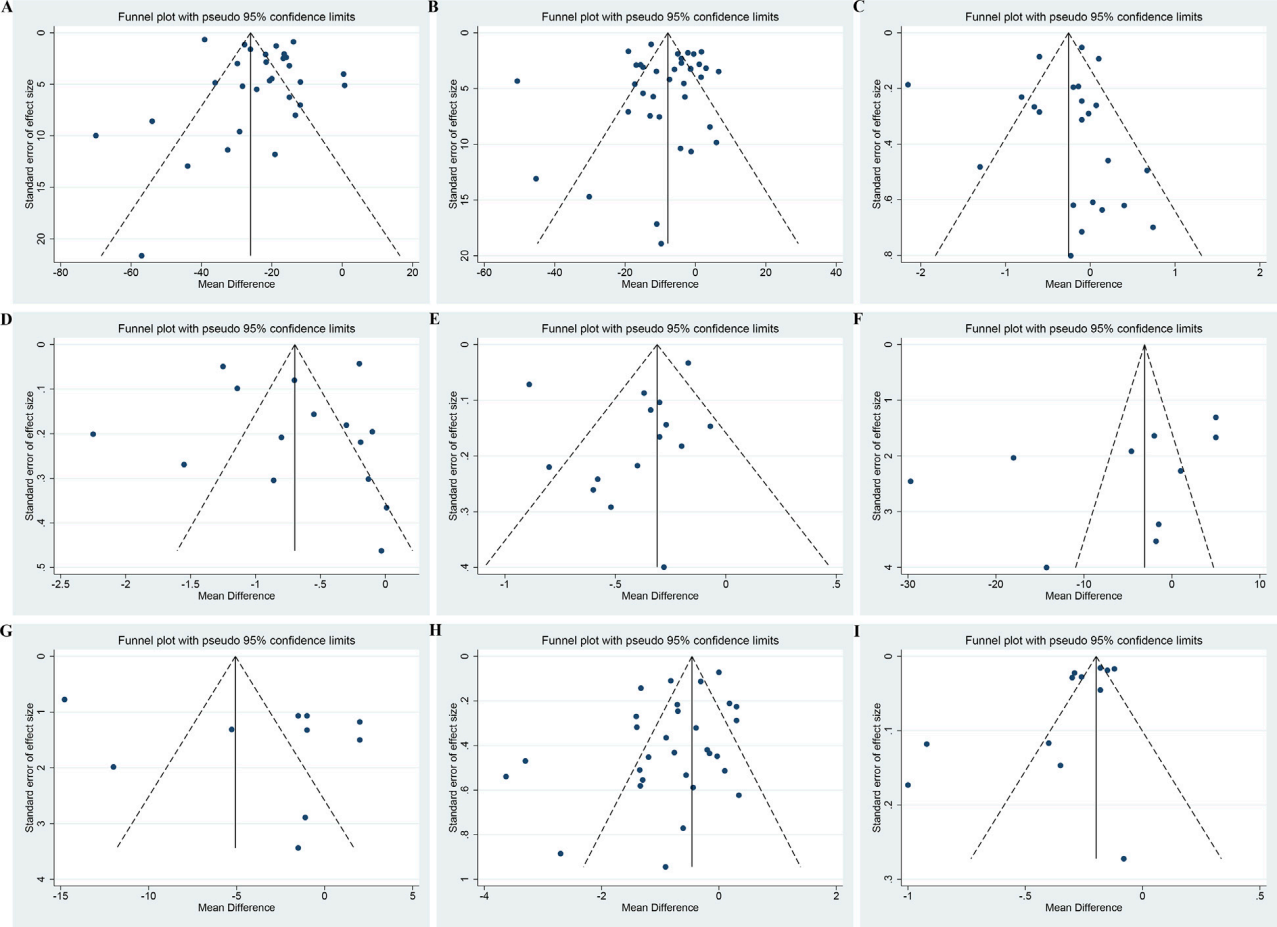
Funnel plots of **(A)** UAER, **(B)** Scr, **(C)** FBG, **(D)** TC, **(E)** TG, **(F)** SBP, **(G)** DBP, **(H)** BUN and **(I)** 24hUTP.

For UAER, Scr, FBG, TC, TG, SBP, and DBP, both the funnel plots (Figure 13A–G) and Egger’s test (*p* = 0.20, 0.93, 0.69, 0.86, 0.25, 0.17 and 0.27, respectively) suggested no obvious publication bias (Supplementary Material S8). For BUN and 24hUTP, the funnel plots (Figures 13H, I) and Egger’s test (*p* = 0.04 and 0.03, respectively) indicated potential publication bias (Supplementary Material S8). The trim and fill method was employed to correct for publication bias. The estimated effect size after trimming and filling showed little change compared with the original results, implying that the publication bias did not affect the results significantly and the results were robust (Supplementary Material S8).

### 3.9 Assessment of evidence quality

According to the GRADE method, among the 22 outcomes evaluated, five were of moderate quality (Cys-C, hs-CRP, TNF-α, hematocrit, safety), seven were of low quality (UAER, SCr, FBG, TC, TG, LDL-C, fibrinogen) and ten were of very low quality (BUN, 24hUTP, 2hPG, HbA1c, SBP, DBP, MDA, SOD, AOPP, IL-6) (Supplementary Material S9).

## 4 Discussion

### 4.1 Main results of this research

Effective treatment for DKD is of great importance in delaying disease progression, reducing cardiovascular and renal endpoints, and improving life quality and survival rate (Wang and Zhang, 2024). The ACEI/ARBs are recommended as first-line medications in guidelines for their benefits in renal protection independent of blood pressure control (Chinese Diabetes Society, 2021; American Diabetes Association, 2024). However, the renal function of some patients continues to deteriorate (Barrera-Chimal and Jaisser, 2020). Therefore, there is an urgent need to explore additional intervention measures to delay the disease progression. Many clinical studies have shown that GBE, one of the most influential herbal products, has better efficacy in treating DKD.

Our meta-analysis found that combining GBE with ACEI/ARB was more effective than ACEI/ARB alone in improving renal function, including UAER, Scr, BUN, 24hUTP and Cys-C levels, impling that GBE may serve as a beneficial complementary treatment. There was large heterogeneity observed in the results for UAER, Scr, BUN and 24hUTP. We hypothesized it might be associated with the uneven methodological quality. As DKD gradually progresses, different stages will appear in sequence, including the hyperfiltration, microalbuminuria, and macroalbuminuria stages. Different severity and stages may result in different responsiveness to drug therapy. Furthermore, the variation in treatment duration and drug dosages between study points could also lead to differences in efficacy. These factors could lead to methodological and clinical heterogeneity, resulting in statistical heterogeneity.

DKD is often complicated by dyslipidemia due to metabolic disorders, and elevated blood lipids, in turn, act as risk factors that further accelerate renal disease progression and increase the risk of cardiovascular events (ABCD and Renal Association, 2021). Studies have shown that each 1% reduction in LDL-C reduced the combined endpoint of coronary death and myocardial infarction by 22%, the major vascular events by 21% and the all-cause death by 9% (Kearney et al., 2008). Our meta-analysis revealed that combination treatment could further reduce the TC, TG and LDL-C levels, meaning that the combination with GBE is suitable for DKD patients with dyslipidemia and may have the potential to reduce endpoint events. Given that the combination treatment has a weak impact on blood glucose, with significant effects on FBG but no significant improvement on 2hPG and HbA1c, it is still necessary to maintain a basic hypoglycemic regimen when using GBE. What’s more, the combination treatment had no significant effect on SBP and DBP, meaning the risk of hypotension caused by combination with GBE is relatively low.

Oxidative stress and inflammatory response are crucial contributors to DKD (Charlton et al., 2020; Rayego-Mateos et al., 2020; Vodošek Hojs et al., 2020). Hyperglycemia could induce the overproduction of reactive oxygen species (ROS). The accumulation of ROS directly injures podocytes, mesangial cells and endothelial cells, causing proteinuria and tubulointerstitial fibrosis (Hung et al., 2021; Hu et al., 2024). Oxidative stress can also mediate macrophage infiltration and inflammatory cell recruitment, increase the expression of inflammatory factors as well as the secretion of fibronectin, resulting in kidney injury and fibrosis (Su et al., 2019; Antar et al., 2023). Our findings suggest that combined GBE treatment appears to be beneficial for both oxidative stress and inflammation.

Abnormalities in hemorheology are also a crucial pathological basis for DKD. Diabetic patients experience increased blood viscosity, enhanced red blood cell aggregation and poor microcirculatory blood flow (Lee et al., 2019). This will cause local tissue hypoxia and ischemia, leading to renal function impairment and disease progression (Wang et al., 2021). Our study suggested that combination treatment reduces the hematocrit and fibrinogen levels, meaning that GBE can improve blood fluidity and vascular microcirculation in DKD patients.

Among the 41 included studies, only ten studies reported adverse events, and others did not report whether adverse events occurred during treatment. The results revealed that combined with GBE did not lead to a higher occurrence of negative effects. The most common adverse event was dizziness, followed by skin rash, nausea and vomiting, dry cough, headache and lower limb soreness. It is worth noting that one case of dyspnea was reported in the treatment group and one case of laryngeal edema was reported in the control group (Xing, 2022). With the widespread use of GBE, reports on its safety are gradually increasing. A comprehensive analysis of 11,374 patients treated with GBE from 607 articles (Hu et al., 2017) showed that the incidence of adverse reactions to GBE was 2.6%. The main symptoms were local pain, abdominal distension, skin flushing, allergic reactions and palpitations. These symptoms were relieved after drug withdrawal or symptomatic treatment, and there were no cases of death or sequelae. Evidence suggests that attention should be paid to the adverse effects of GBE to avoid serious adverse events during treatment. Once an adverse event occurs, the medication should be stopped immediately, actively handled and reported. Since we could only conduct safety analysis based on the several studies that reported adverse events, whether the available results on safety are actual or biased in some way is still uncertain. Future studies should focus on monitoring and reporting adverse events.

### 4.2 Study on the internal possible mechanism

Studies have shown that GBE induces the renal nuclear factor erythroid 2-related factor 2/heme oxygenase-1 (Nrf-2/HO-1) pathway expression in high glucose environments, which could decrease ROS release, modulate inflammatory factors expression and fibronectin production, reduce renal podocyte injury and lipid accumulation as well as ameliorate glomerular hypertrophy (Chang et al., 2021). GBE can also reduce collagen accumulation and laminin expression and inhibit the transformation of renal tubular epithelial cells from epithelial to mesenchymal phenotype by regulating the protein kinase B/mammalian target of rapamycin (Akt/mTOR) signaling pathway, thereby reducing glomerular basement membrane thickening (Lu et al., 2015). It could downregulate tissue transglutaminase (tTG) expression by inhibiting transforming growth factor-β (TGF-β), which can reduce the accumulation of extracellular matrix constituents such as fibronectin, collagen and collagen peptides (Yu et al., 2021). It was also found that GBE significantly reduced 78-kDa glucose-regulated protein (GRP78) and activating transcription factor 6 (ATF6) protein expression in diabetic renal tissues, suggesting that GBE may attenuate renal injury by inhibiting endoplasmic reticulum stress (Pang et al., 2020). In addition, ginkgetin may activate the adenosine 5′-monophosphate-activated protein kinase/mammalian target of rapamycin (AMPK/mTOR)-mediated autophagy pathway to attenuate mesangial cell proliferation and extracellular matrix accumulation (Wei et al., 2021). The above pathways may be the internal molecular mechanisms by which GBE exerts its renal protective effects.

### 4.3 Limitation of this study

There are several potential limitations in this meta-analysis. Firstly, although we did not restrict literature characteristics during searching and screening, all included studies were conducted in China and were single-center studies, which may affect the generalization of the results. Secondly, the methodological rigor of many studies is not high, raising concerns about potential biases. And our further exploration on heterogeneity sources was limited due to inadequate reporting on study characteristics such as average age or disease stage. Thirdly, all studies’ treatment and follow-up durations were short, and there was no evidence regarding the long-term efficacy and cardiorenal benefits for combined GBE treatment. Fourthly, there were large differences between studies in the selection of outcome indicators. Some parameters, such as MDA, SOD, AOPP, hs-CRP, TNF-α and IL-6, were reported less frequently, leading to possible instability. Finally, adverse reactions went unmonitored and unreported in most studies, potentially impacting the evaluation of safety. Although these limitations exist objectively, we prefer to maintain an optimistic attitude. Identifying deficiencies through our current study can provide a foundation for further research.

### 4.4 Implication for clinical practice and future research

Some recommendations should be addressed in future research. Firstly, the Standard Protocol Items: Recommendations for Interventional Trials (SPIRIT) and Consolidated Standards of Reporting Trials (CONSORT) statements should be strictly followed when designing and reporting RCTs. For example, registration in advance to reduce deviations from the study design. Properly implement randomization and allocation concealment, and use double-blinding or triple-blinding to reduce potential methodological bias. Secondly, future RCTs should recruit larger multicenter cohorts with diverse populations to improve the generalizability of the findings. Thirdly, we stress monitoring adverse events closely during treatment and implementing rigorous procedures for handling and reporting them, to provide more safety information for GBE. Fourthly, DKD is a chronic progressive disease that requires ongoing treatment. Long-term and follow-up studies are necessary for exploring GBE’s long-term efficacy on cardiorenal endpoints. Fifthly, it is necessary to record the study characteristics clearly to better analyze the sources of heterogeneity and explore dominant populations. A complete core indicator set for DKD should be established, and more representative and convincing indicators should be selected when designing the protocol. Last but not least, further research should be conducted on the interaction between GBE and common hypoglycemic and antihypertensive drugs, which would help determine the safety and rationality of combined usage. We encourage future studies to implement these recommendations to gain a deeper understanding on the benefits and safety of GBE, thus providing higher-quality evidence for decision-making in DKD treatment.

## 5 Conclusion

The available evidence suggests that combining GBE with ACEI/ARB may be more effective in improving UAER, SCr, BUN, 24hUTP, Cys-C, TC, TG, LDL-C, hematocrit and fibrinogen. It also seems beneficial for oxidative stress and inflammation but has minimal impact on glucose and blood pressure. Although combined GBE treatment is generally tolerated, its safety needs to be further monitored given the potential adverse effects. Due to the uneven methodological quality and high heterogeneity, the overall strength of evidence is not high. In the future, additional rigorous clinical trials and thorough molecular investigations are imperative to furnish robust evidence regarding the benefits and safety of GBE in DKD, thereby offering new directions and possibilities for the management of DKD.

## Data Availability

The original contributions presented in the study are included in the article/Supplementary Material, further inquiries can be directed to the corresponding authors.

## Abbreviations

24hUTP, 24-hour urinary total protein; 2hPG, 2-hour postprandial glucose; ACEIs, angiotensin-converting enzyme inhibitors; AOPP, advanced oxidation protein product; ARBs, angiotensin II receptor blockers; BUN, blood urea nitrogen; CG, control group interventions; CI, confidence interval; Cys-C, cystatin C; DBP, diastolic blood pressure; DKD, diabetic kidney disease; ESRD, end-stage renal disease; FBG, fasting blood glucose; GBE, *Ginkgo biloba* L. leaves extract; HbA1c, glycated hemoglobin; hs-CRP, high-sensitivity C-reactive protein; IL-6, interleukin-6; LDL-C, low-density lipoprotein cholesterol; MD, mean difference; MDA, malondialdehyde; NR, not reported; NSD, no significant difference; RAAS, renin-angiotensin-aldosterone system; RCTs, randomized controlled trials; RR, relative risk; SBP, systolic blood pressure; SCr, serum creatinine; SMD, standardized mean difference; SOD, superoxide dismutase; TC, total cholesterol; TG, triglyceride; TNF-α, tumor necrosis factor-α; UAER, urinary albumin excretion rate.
