# Supplementary material for: The efficacy and safety of Ginkgo biloba L. leaves extract combined with ACEI/ARB on diabetic kidney disease: a systematic review and meta-analysis of 41 randomized controlled trials
Source: Front Pharmacol. 2025 Jan 3;15:1408546. doi: 10.3389/fphar.2024.1408546 (PMC11739094; doi:10.3389/fphar.2024.1408546)
Supplement: Supplementary file 1 [file Table1.docx]

**Contents Page**

[Supplementary Material S1. PRISMA 2020 checklist 1](#_Toc174042810)

[Supplementary Material S2. Database and Search Strategies 4](#_Toc174042811)

[Supplementary Material S3. Literature excluded after reading the full text and reasons 12](#_Toc174042812)

[Supplementary Material S4. Detailed information of GBE formulations accroding to the ConPhyMP statement 26](#_Toc174042813)

[Supplementary Material S5. Meta-regression of UAER, Scr, BUN, 24hUTP, FBG, TC and TG 38](#_Toc174042814)

[Supplementary Material S6. Subgroup analysis of UAER, Scr, BUN, 24hUTP, FBG, TC, TG, SBP and DBP. 43](#_Toc174042815)

[Supplementary Material S7. Sensitivity analysis. 51](#_Toc174042816)

[Supplementary Material S8. Publication Bias 56](#_Toc174042817)

[Supplementary Material S9. Assessment of evidence quality for each outcome. 59](#_Toc174042818)

# Supplementary Material S1. PRISMA 2020 checklist

| **Section and Topic** | **Item #** | **Checklist item** | **Location where item is reported** |
| --- | --- | --- | --- |
| **TITLE** | | |  |
| Title | 1 | Identify the report as a systematic review. | Title |
| **ABSTRACT** | | |  |
| Abstract | 2 | See the PRISMA 2020 for Abstracts checklist. | Abstract |
| **INTRODUCTION** | | |  |
| Rationale | 3 | Describe the rationale for the review in the context of existing knowledge. | Introduction |
| Objectives | 4 | Provide an explicit statement of the objective(s) or question(s) the review addresses. | Introduction |
| **METHODS** | | |  |
| Eligibility criteria | 5 | Specify the inclusion and exclusion criteria for the review and how studies were grouped for the syntheses. | Materials and Methods |
| Information sources | 6 | Specify all databases, registers, websites, organisations, reference lists and other sources searched or consulted to identify studies. Specify the date when each source was last searched or consulted. | Materials and Methods |
| Search strategy | 7 | Present the full search strategies for all databases, registers and websites, including any filters and limits used. | Supplementary S2 |
| Selection process | 8 | Specify the methods used to decide whether a study met the inclusion criteria of the review, including how many reviewers screened each record and each report retrieved, whether they worked independently, and if applicable, details of automation tools used in the process. | Materials and Methods |
| Data collection process | 9 | Specify the methods used to collect data from reports, including how many reviewers collected data from each report, whether they worked independently, any processes for obtaining or confirming data from study investigators, and if applicable, details of automation tools used in the process. | Materials and Methods |
| Data items | 10a | List and define all outcomes for which data were sought. Specify whether all results that were compatible with each outcome domain in each study were sought (e.g. for all measures, time points, analyses), and if not, the methods used to decide which results to collect. | Materials and Methods |
|  | 10b | List and define all other variables for which data were sought (e.g. participant and intervention characteristics, funding sources). Describe any assumptions made about any missing or unclear information. | Materials and Methods |
| Study risk of bias assessment | 11 | Specify the methods used to assess risk of bias in the included studies, including details of the tool(s) used, how many reviewers assessed each study and whether they worked independently, and if applicable, details of automation tools used in the process. | Materials and Methods |
| Effect measures | 12 | Specify for each outcome the effect measure(s) (e.g. risk ratio, mean difference) used in the synthesis or presentation of results. | Materials and Methods |
| Synthesis methods | 13a | Describe the processes used to decide which studies were eligible for each synthesis (e.g. tabulating the study intervention characteristics and comparing against the planned groups for each synthesis (item #5)). | Materials and Methods |
|  | 13b | Describe any methods required to prepare the data for presentation or synthesis, such as handling of missing summary statistics, or data conversions. | Materials and Methods |
|  | 13c | Describe any methods used to tabulate or visually display results of individual studies and syntheses. | Materials and Methods |
|  | 13d | Describe any methods used to synthesize results and provide a rationale for the choice(s). If meta-analysis was performed, describe the model(s), method(s) to identify the presence and extent of statistical heterogeneity, and software package(s) used. | Materials and Methods |
|  | 13e | Describe any methods used to explore possible causes of heterogeneity among study results (e.g. subgroup analysis, meta-regression). | Materials and Methods |
|  | 13f | Describe any sensitivity analyses conducted to assess robustness of the synthesized results. | Materials and Methods |
| Reporting bias assessment | 14 | Describe any methods used to assess risk of bias due to missing results in a synthesis (arising from reporting biases). | Materials and Methods |
| Certainty assessment | 15 | Describe any methods used to assess certainty (or confidence) in the body of evidence for an outcome. | Materials and Methods |
| **RESULTS** | | |  |
| Study selection | 16a | Describe the results of the search and selection process, from the number of records identified in the search to the number of studies included in the review, ideally using a flow diagram. | Results, figure 1 |
|  | 16b | Cite studies that might appear to meet the inclusion criteria, but which were excluded, and explain why they were excluded. | Supplementary Material S3 |
| Study characteristics | 17 | Cite each included study and present its characteristics. | Results, table 1 |
| Risk of bias in studies | 18 | Present assessments of risk of bias for each included study. | Results, figure 2 |
| Results of individual studies | 19 | For all outcomes, present, for each study: (a) summary statistics for each group (where appropriate) and (b) an effect estimate and its precision (e.g. confidence/credible interval), ideally using structured tables or plots. | Results |
| Results of syntheses | 20a | For each synthesis, briefly summarise the characteristics and risk of bias among contributing studies. | Results |
|  | 20b | Present results of all statistical syntheses conducted. If meta-analysis was done, present for each the summary estimate and its precision (e.g. confidence/credible interval) and measures of statistical heterogeneity. If comparing groups, describe the direction of the effect. | Results |
|  | 20c | Present results of all investigations of possible causes of heterogeneity among study results. | Results |
|  | 20d | Present results of all sensitivity analyses conducted to assess the robustness of the synthesized results. | Results |
| Reporting biases | 21 | Present assessments of risk of bias due to missing results (arising from reporting biases) for each synthesis assessed. | Results |
| Certainty of evidence | 22 | Present assessments of certainty (or confidence) in the body of evidence for each outcome assessed. | Results,Supplementary Material S9 |
| **DISCUSSION** | | |  |
| Discussion | 23a | Provide a general interpretation of the results in the context of other evidence. | Discussion |
|  | 23b | Discuss any limitations of the evidence included in the review. | Discussion |
|  | 23c | Discuss any limitations of the review processes used. | Discussion |
|  | 23d | Discuss implications of the results for practice, policy, and future research. | Discussion |
| **OTHER INFORMATION** | | |  |
| Registration and protocol | 24a | Provide registration information for the review, including register name and registration number, or state that the review was not registered. | Materials and Methods,CRD42023455792 |
|  | 24b | Indicate where the review protocol can be accessed, or state that a protocol was not prepared. | - |
|  | 24c | Describe and explain any amendments to information provided at registration or in the protocol. | - |
| Support | 25 | Describe sources of financial or non-financial support for the review, and the role of the funders or sponsors in the review. | Funding |
| Competing interests | 26 | Declare any competing interests of review authors. | Conflict of Interest Statement |
| Availability of data, code and other materials | 27 | Report which of the following are publicly available and where they can be found: template data collection forms; data extracted from included studies; data used for all analyses; analytic code; any other materials used in the review. | Data Availability Statement |

*From:* Page MJ, McKenzie JE, Bossuyt PM, Boutron I, Hoffmann TC, Mulrow CD, et al. The PRISMA 2020 statement: an updated guideline for reporting systematic reviews. BMJ 2021;372:n71. doi: 10.1136/bmj.n71 For more information, visit: http://www.prisma-statement.org/

# Supplementary Material S2. Database and Search Strategies

PubMed

The retrieval of the PubMed database was conducted on July 21, 2023, and 28 record was retrieved.

| Search | Query | Results | Time |
| --- | --- | --- | --- |
| #7 | Search: (("Diabetic Nephropathies"[Mesh]) OR (((((((((((((((((Nephropathies, Diabetic[Title/Abstract]) OR (Nephropathy, Diabetic[Title/Abstract])) OR (Diabetic Nephropathy[Title/Abstract])) OR (Diabetic Nephropathy[Title/Abstract])) OR (Diabetic Kidney Diseases[Title/Abstract])) OR (Kidney Disease, Diabetic[Title/Abstract])) OR (Kidney Diseases, Diabetic[Title/Abstract])) OR (Diabetic Glomerulosclerosis[Title/Abstract])) OR (Glomerulosclerosis, Diabetic[Title/Abstract])) OR (Intracapillary Glomerulosclerosis[Title/Abstract])) OR (Nodular Glomerulosclerosis[Title/Abstract])) OR (Glomerulosclerosis, Nodular[Title/Abstract])) OR (Kimmelstiel-Wilson Syndrome[Title/Abstract])) OR (Kimmelstiel Wilson Syndrome[Title/Abstract])) OR (Syndrome, Kimmelstiel-Wilson[Title/Abstract])) OR (Kimmelstiel-Wilson Disease[Title/Abstract])) OR (Kimmelstiel Wilson Disease[Title/Abstract]))) AND (("Ginkgo biloba extract" [Supplementary Concept]) OR (((((((((((((((((((Extract of Ginkgo biloba[Title/Abstract]) OR (Ginkgo leaf extract[Title/Abstract])) OR (Ginkgo biloba[Title/Abstract])) OR (Rokan[Title/Abstract])) OR (Tanakan[Title/Abstract])) OR (Ginaton[Title/Abstract])) OR (Jinnaduo[Title/Abstract])) OR (Danakang[Title/Abstract])) OR (Yikangning[Title/Abstract])) OR (GBE 761[Title/Abstract])) OR (Ginkgo biloba extract 761[Title/Abstract])) OR (GBE-761[Title/Abstract])) OR (Tebofortran[Title/Abstract])) OR (Tebokan[Title/Abstract])) OR (Tebonin[Title/Abstract])) OR (EGb 761[Title/Abstract])) OR (GBE 761 ONC[Title/Abstract])) OR (EGb-761[Title/Abstract])) OR (EGb761[Title/Abstract]))) Sort by: Most Recent | 28 | 08:08:54 |
| #6 | Search: ("Ginkgo biloba extract" [Supplementary Concept]) OR (((((((((((((((((((Extract of Ginkgo biloba[Title/Abstract]) OR (Ginkgo leaf extract[Title/Abstract])) OR (Ginkgo biloba[Title/Abstract])) OR (Rokan[Title/Abstract])) OR (Tanakan[Title/Abstract])) OR (Ginaton[Title/Abstract])) OR (Jinnaduo[Title/Abstract])) OR (Danakang[Title/Abstract])) OR (Yikangning[Title/Abstract])) OR (GBE 761[Title/Abstract])) OR (Ginkgo biloba extract 761[Title/Abstract])) OR (GBE-761[Title/Abstract])) OR (Tebofortran[Title/Abstract])) OR (Tebokan[Title/Abstract])) OR (Tebonin[Title/Abstract])) OR (EGb 761[Title/Abstract])) OR (GBE 761 ONC[Title/Abstract])) OR (EGb-761[Title/Abstract])) OR (EGb761[Title/Abstract])) Sort by: Most Recent | 4,574 | 08:08:27 |
| #5 | Search: ((((((((((((((((((Extract of Ginkgo biloba[Title/Abstract]) OR (Ginkgo leaf extract[Title/Abstract])) OR (Ginkgo biloba[Title/Abstract])) OR (Rokan[Title/Abstract])) OR (Tanakan[Title/Abstract])) OR (Ginaton[Title/Abstract])) OR (Jinnaduo[Title/Abstract])) OR (Danakang[Title/Abstract])) OR (Yikangning[Title/Abstract])) OR (GBE 761[Title/Abstract])) OR (Ginkgo biloba extract 761[Title/Abstract])) OR (GBE-761[Title/Abstract])) OR (Tebofortran[Title/Abstract])) OR (Tebokan[Title/Abstract])) OR (Tebonin[Title/Abstract])) OR (EGb 761[Title/Abstract])) OR (GBE 761 ONC[Title/Abstract])) OR (EGb-761[Title/Abstract])) OR (EGb761[Title/Abstract]) Sort by: Most Recent | 4,484 | 08:07:53 |
| #4 | Search: "Ginkgo biloba extract" [Supplementary Concept] Sort by: Most Recent | 1,156 | 08:05:14 |
| #3 | Search: ("Diabetic Nephropathies"[Mesh]) OR (((((((((((((((((Nephropathies, Diabetic[Title/Abstract]) OR (Nephropathy, Diabetic[Title/Abstract])) OR (Diabetic Nephropathy[Title/Abstract])) OR (Diabetic Nephropathy[Title/Abstract])) OR (Diabetic Kidney Diseases[Title/Abstract])) OR (Kidney Disease, Diabetic[Title/Abstract])) OR (Kidney Diseases, Diabetic[Title/Abstract])) OR (Diabetic Glomerulosclerosis[Title/Abstract])) OR (Glomerulosclerosis, Diabetic[Title/Abstract])) OR (Intracapillary Glomerulosclerosis[Title/Abstract])) OR (Nodular Glomerulosclerosis[Title/Abstract])) OR (Glomerulosclerosis, Nodular[Title/Abstract])) OR (Kimmelstiel-Wilson Syndrome[Title/Abstract])) OR (Kimmelstiel Wilson Syndrome[Title/Abstract])) OR (Syndrome, Kimmelstiel-Wilson[Title/Abstract])) OR (Kimmelstiel-Wilson Disease[Title/Abstract])) OR (Kimmelstiel Wilson Disease[Title/Abstract])) Sort by: Most Recent | 38,196 | 08:03:41 |
| #2 | Search: ((((((((((((((((Nephropathies, Diabetic[Title/Abstract]) OR (Nephropathy, Diabetic[Title/Abstract])) OR (Diabetic Nephropathy[Title/Abstract])) OR (Diabetic Nephropathy[Title/Abstract])) OR (Diabetic Kidney Diseases[Title/Abstract])) OR (Kidney Disease, Diabetic[Title/Abstract])) OR (Kidney Diseases, Diabetic[Title/Abstract])) OR (Diabetic Glomerulosclerosis[Title/Abstract])) OR (Glomerulosclerosis, Diabetic[Title/Abstract])) OR (Intracapillary Glomerulosclerosis[Title/Abstract])) OR (Nodular Glomerulosclerosis[Title/Abstract])) OR (Glomerulosclerosis, Nodular[Title/Abstract])) OR (Kimmelstiel-Wilson Syndrome[Title/Abstract])) OR (Kimmelstiel Wilson Syndrome[Title/Abstract])) OR (Syndrome, Kimmelstiel-Wilson[Title/Abstract])) OR (Kimmelstiel-Wilson Disease[Title/Abstract])) OR (Kimmelstiel Wilson Disease[Title/Abstract]) Sort by: Most Recent | 23,480 | 08:03:25 |
| #1 | Search: "Diabetic Nephropathies"[Mesh] Sort by: Most Recent | 29,489 | 08:00:57 |

Embase

The retrieval of the PubMed database was conducted on July 21, 2023, and 46 record was retrieved.

| History |  | Results |
| --- | --- | --- |
| #7 | #3 AND #6 | 46 |
| #6 | #4 OR #5 | 10,304 |
| #5 | 'extract of ginkgo biloba':ab,ti OR 'ginkgo leaf extract':ab,ti OR 'ginkgo biloba':ab,ti OR rokan:ab,ti OR tanakan:ab,ti OR ginaton:ab,ti OR jinnaduo:ab,ti OR danakang:ab,ti OR yikangning:ab,ti OR 'ginkgo biloba extract 761':ab,ti OR 'gbe 761':ab,ti OR tebofortran:ab,ti OR tebokan:ab,ti OR tebonin:ab,ti OR 'gbe 761 onc':ab,ti OR 'egb 761':ab,ti OR egb761:ab,ti | 5,917 |
| #4 | 'ginkgo biloba extract'/exp | 7,988 |
| #3 | #1 OR #2 | 56,453 |
| #2 | 'nephropathies, diabetic':ab,ti OR 'nephropathy, diabetic':ab,ti OR 'diabetic nephropathies':ab,ti OR 'diabetic kidney disease':ab,ti OR 'diabetic kidney diseases':ab,ti OR 'kidney disease, diabetic':ab,ti OR 'kidney diseases, diabetic':ab,ti OR 'diabetic glomerulosclerosis':ab,ti OR 'glomerulosclerosis, diabetic':ab,ti OR 'intracapillary glomerulosclerosis':ab,ti OR 'nodular glomerulosclerosis':ab,ti OR 'glomerulosclerosis, nodular':ab,ti OR 'kimmelstiel-wilson syndrome':ab,ti OR 'kimmelstiel wilson syndrome':ab,ti OR 'syndrome, kimmelstiel-wilson':ab,ti OR 'kimmelstiel-wilson disease':ab,ti OR 'kimmelstiel wilson disease':ab,ti | 8,502 |
| #1 | 'diabetic nephropathy'/exp | 54,909 |


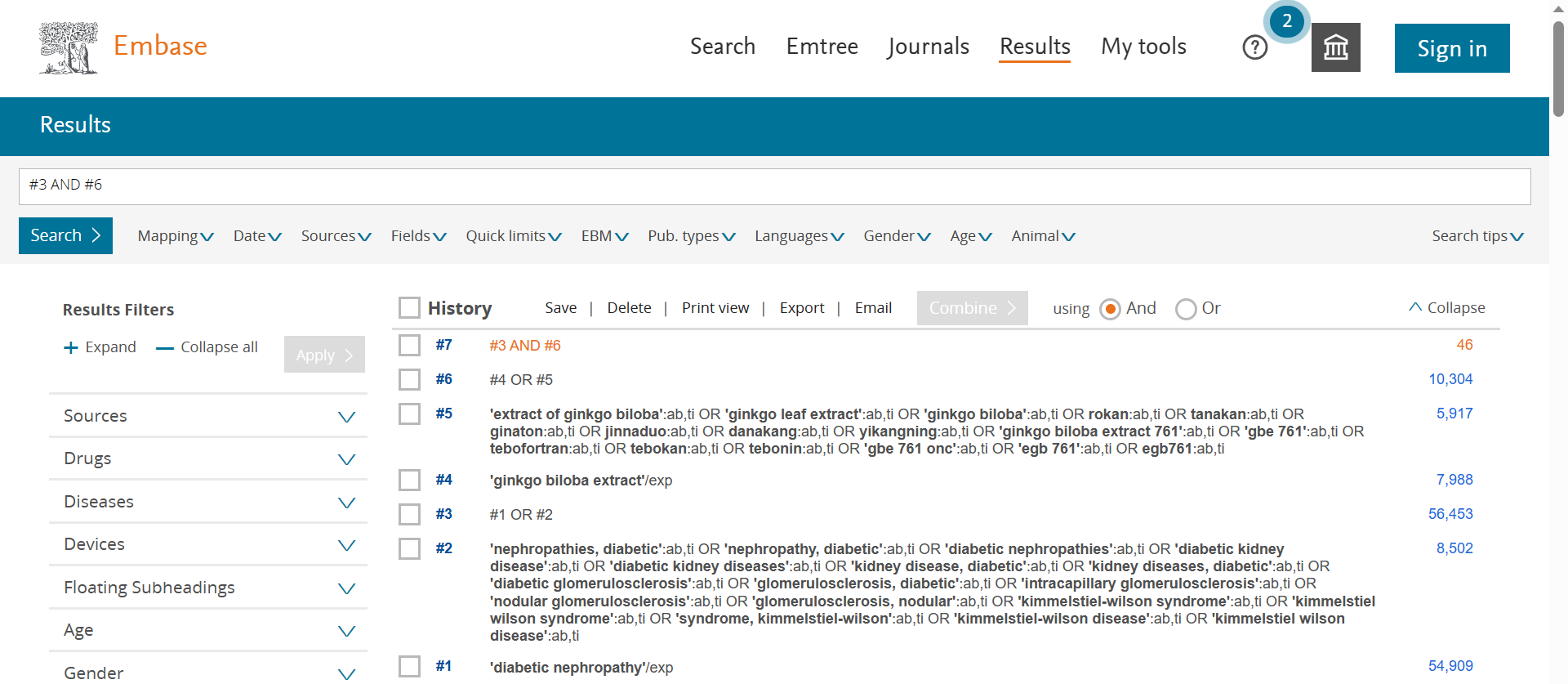


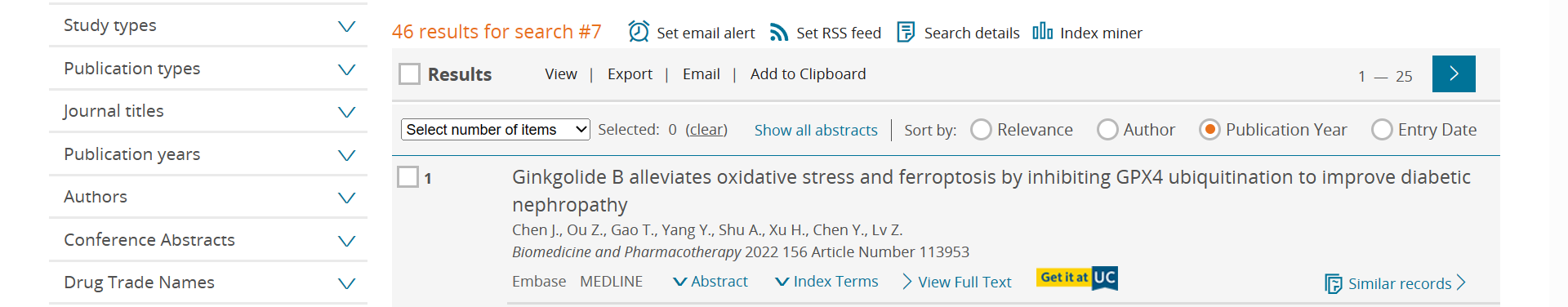


Cochrane

The retrieval of the PubMed database was conducted on July 21, 2023, and 22 record was retrieved.


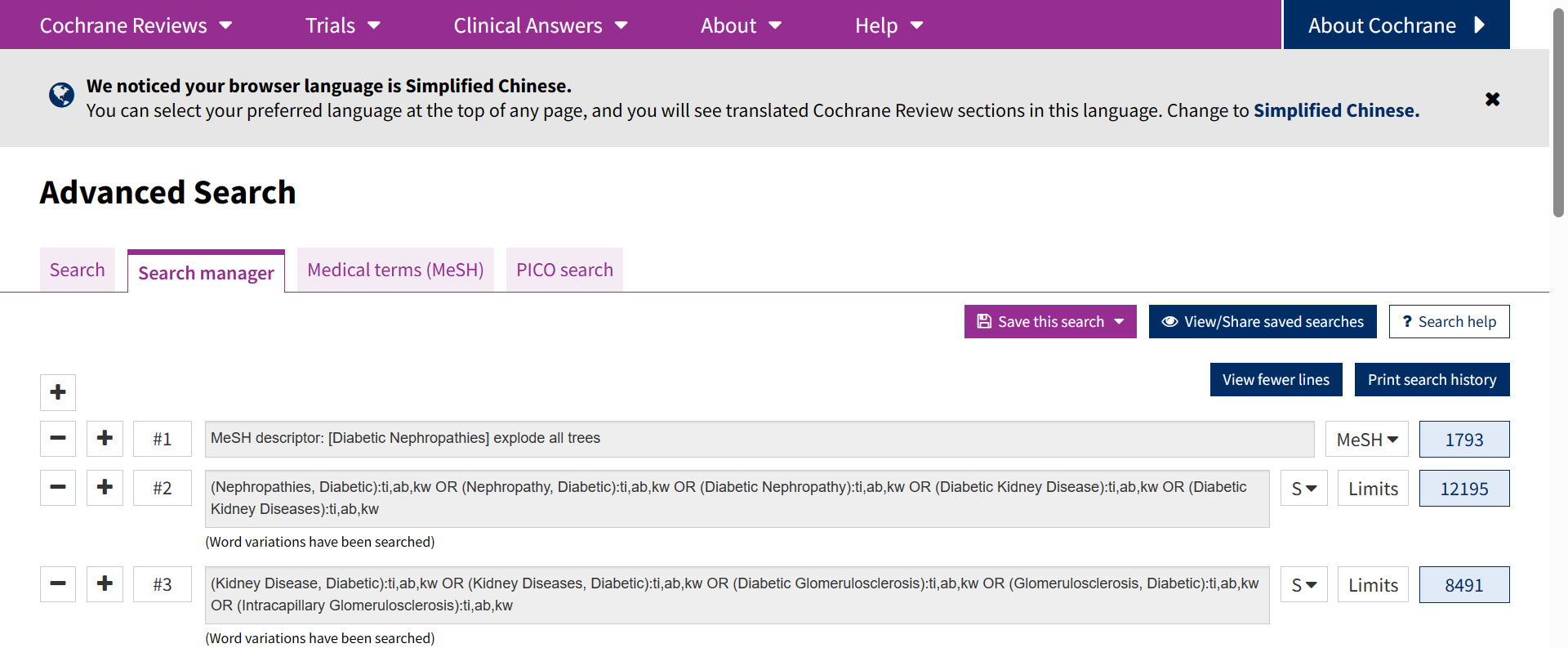


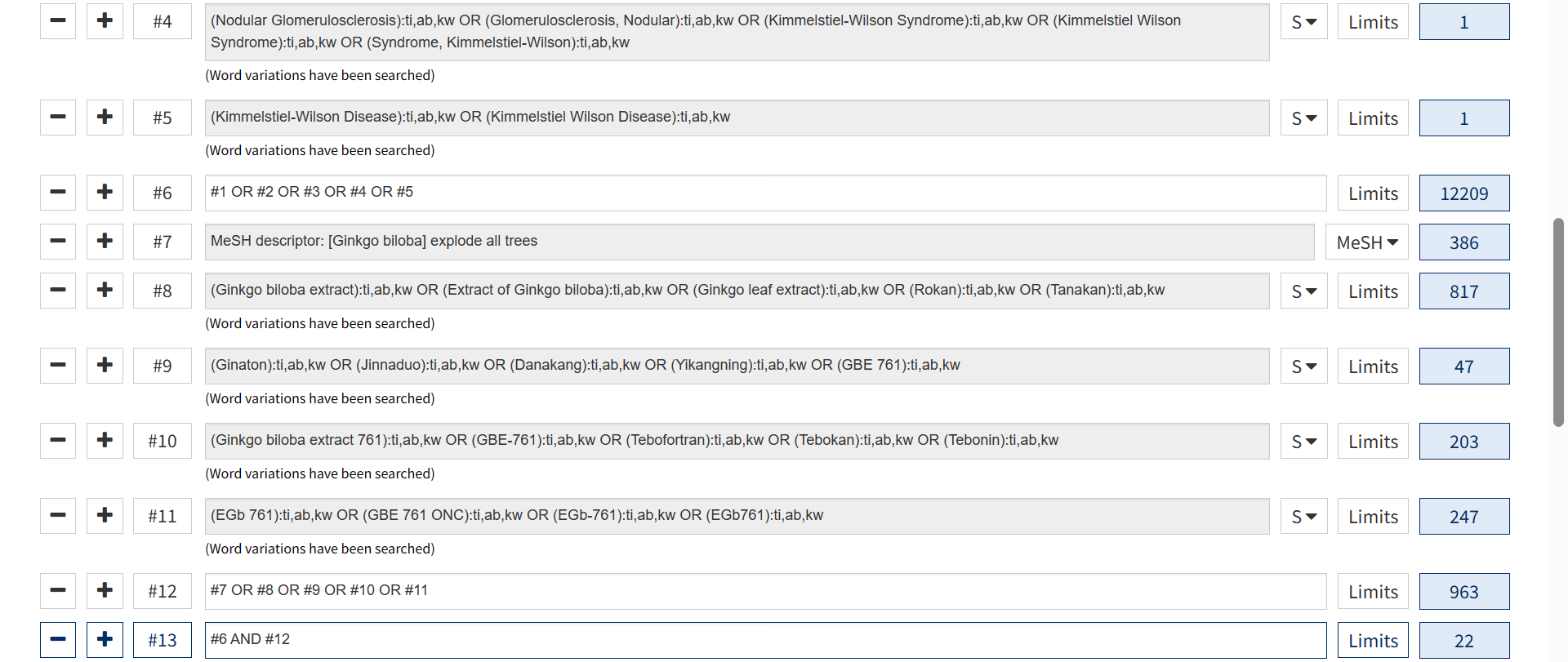


WOS

The retrieval of the PubMed database was conducted on July 21, 2023, and 82 record was retrieved.

| History |  | Results |
| --- | --- | --- |
| #3 | #2 AND #1 | 82 |
| #2 | TS=(Ginkgo biloba extract OR Extract of Ginkgo biloba OR Ginkgo leaf extract OR Ginkgo biloba OR Rokan OR Tanakan OR Ginaton OR Jinnaduo OR Danakang OR Yikangning OR GBE 761 OR Ginkgo biloba extract 761 OR GBE-761 OR Tebofortran OR Tebokan OR Tebonin OR EGb 761 OR GBE 761 ONC OR EGb-761 OR EGb761) | 19009 |
| #1 | TS=(Diabetic Nephropathies OR Nephropathies, Diabetic OR Nephropathy, Diabetic OR Diabetic Nephropathy OR Diabetic Nephropathy OR Diabetic Kidney Diseases OR Kidney Disease, Diabetic OR Kidney Diseases, Diabetic OR Diabetic Glomerulosclerosis OR Glomerulosclerosis, Diabetic OR Intracapillary Glomerulosclerosis OR Nodular Glomerulosclerosis OR Glomerulosclerosis, Nodular OR Kimmelstiel-Wilson Syndrome OR Kimmelstiel Wilson Syndrome OR Syndrome, Kimmelstiel-Wilson OR Kimmelstiel-Wilson Disease OR Kimmelstiel Wilson Disease) | 100616 |


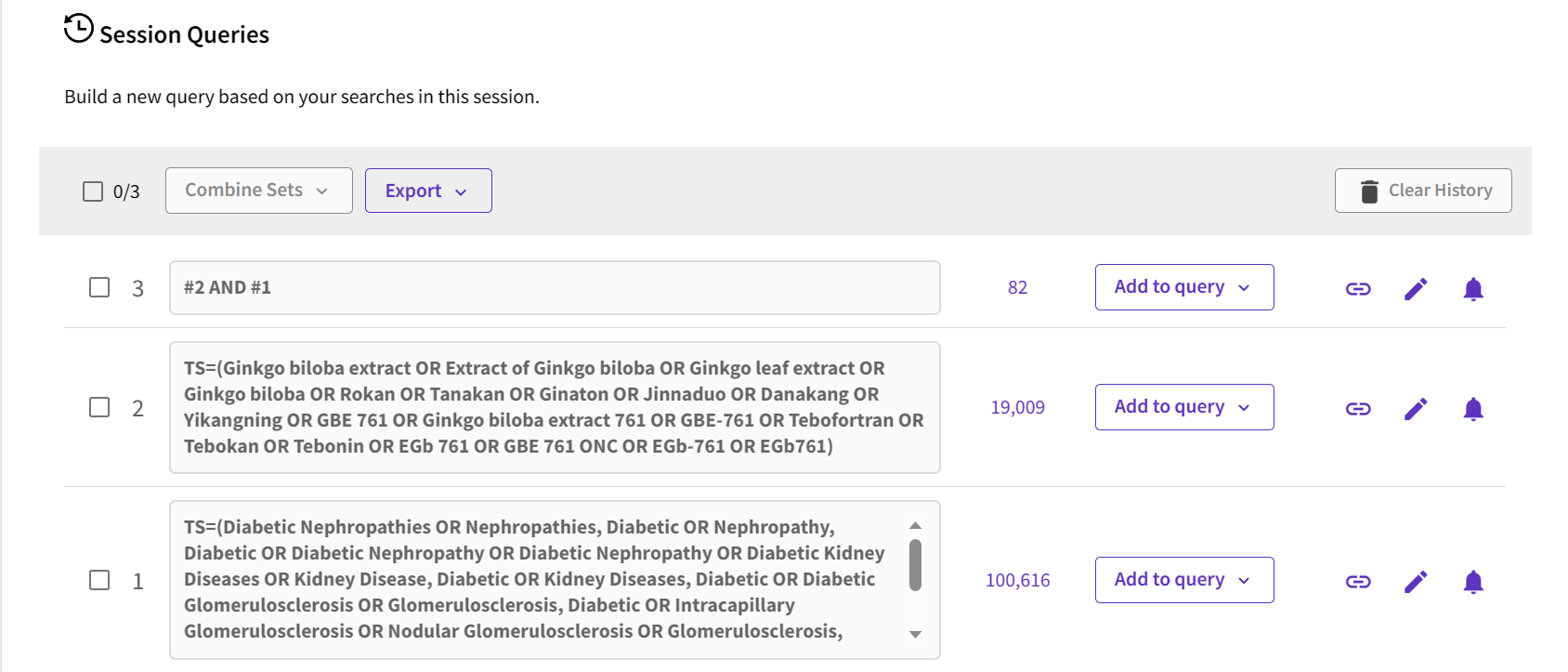


CNKI

The retrieval of the CNKI database was conducted on July 21, 2023, and a total of 365 records were retrieved.

(SU='银杏叶提取物'+'银杏'+'银杏叶'+'白果叶'+'金纳多'+'达纳康'+'依康宁' OR TKA='银杏叶提取物'+'银杏'+'银杏叶'+'白果叶'+'金纳多'+'达纳康'+'依康宁') AND (SU='糖尿病肾病'+'糖尿病性肾病'+'糖尿病肾脏病'+'糖尿病性肾脏病'+'糖尿病肾脏疾病'+'糖尿病肾脏疾病'+'消渴肾病'+'消渴肾衰'+'消渴肾风' OR TKA='糖尿病肾病'+'糖尿病性肾病'+'糖尿病肾脏病'+'糖尿病性肾脏病'+'糖尿病肾脏疾病'+'糖尿病肾脏疾病'+'消渴肾病'+'消渴肾衰'+'消渴肾风')


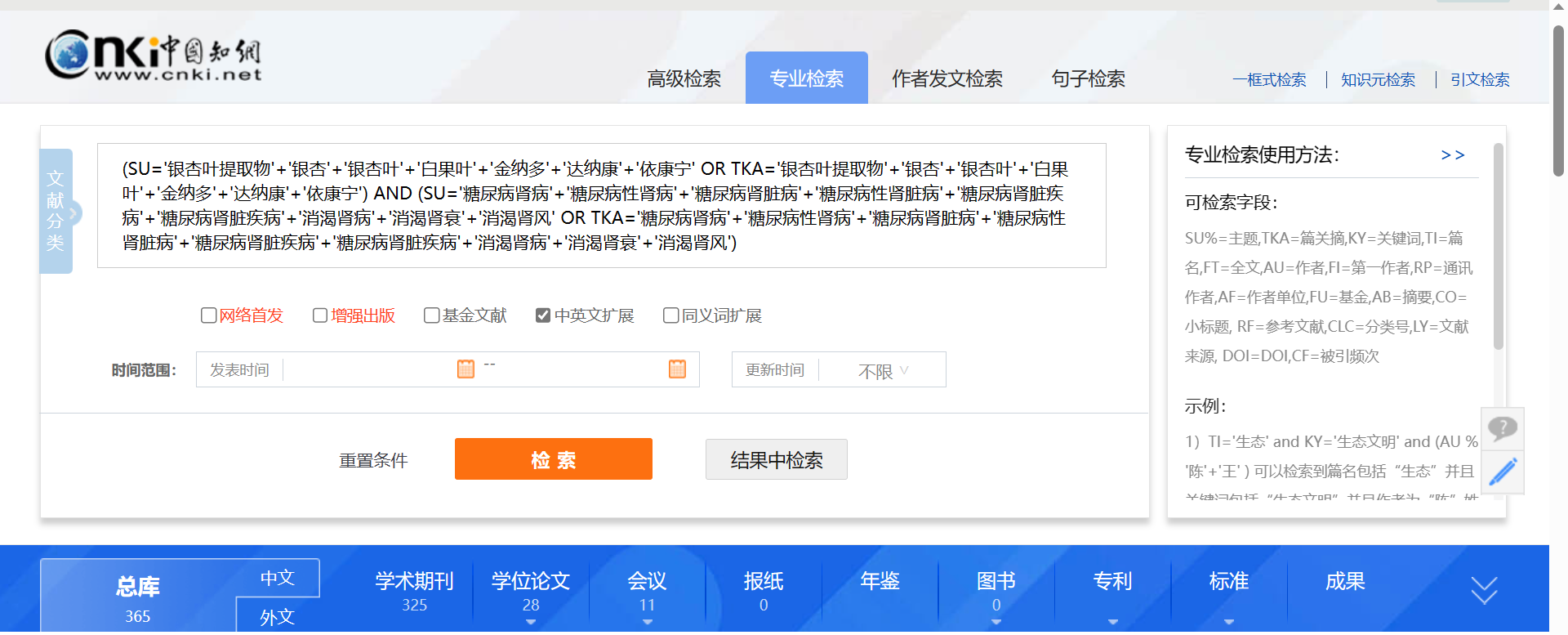


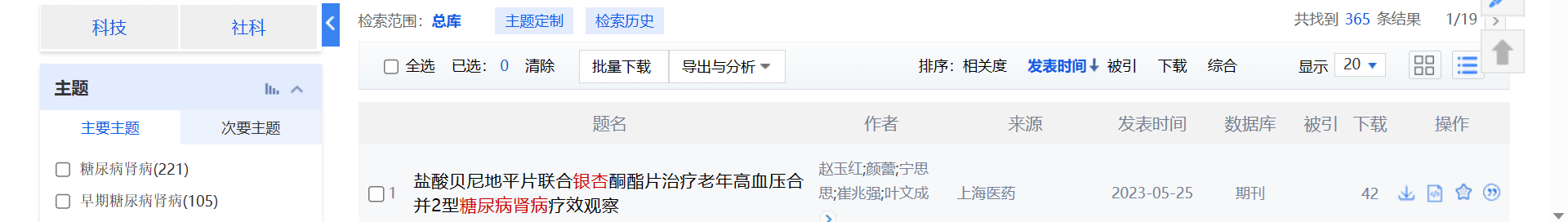


VIP

The retrieval of the VIP database was conducted on July 21, 2023, and a total of 338 records were retrieved.

(M=(银杏叶提取物+银杏+银杏叶+白果叶+金纳多+达纳康+依康宁) OR R=(银杏叶提取物+银杏+银杏叶+白果叶+金纳多+达纳康+依康宁)) AND (M=(糖尿病肾病+糖尿病性肾病+糖尿病肾脏病+糖尿病性肾脏病+糖尿病肾脏疾病+糖尿病性肾脏疾病+消渴肾病+消渴肾衰+消渴肾风) OR R=(糖尿病肾病+糖尿病性肾病+糖尿病肾脏病+糖尿病性肾脏病+糖尿病肾脏疾病+糖尿病性肾脏疾病+消渴肾病+消渴肾衰+消渴肾风))


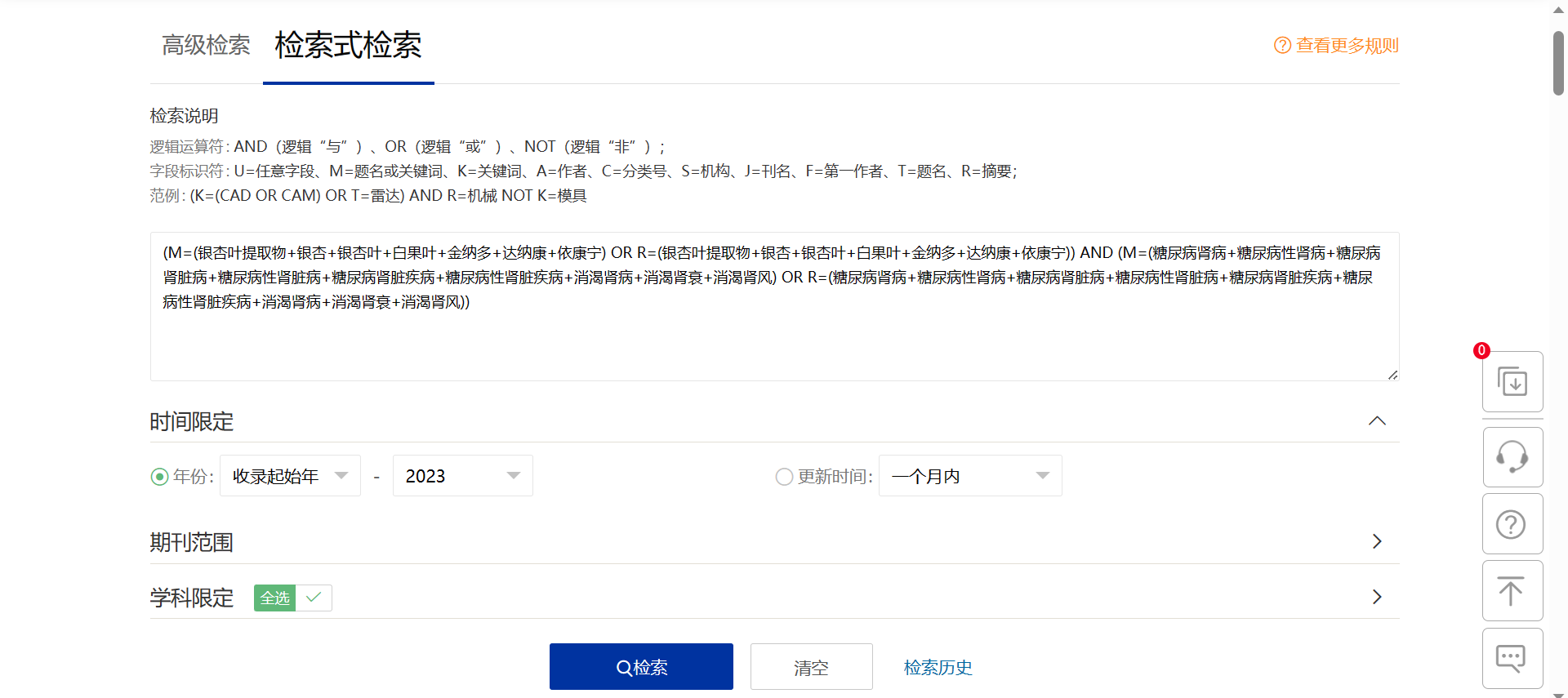


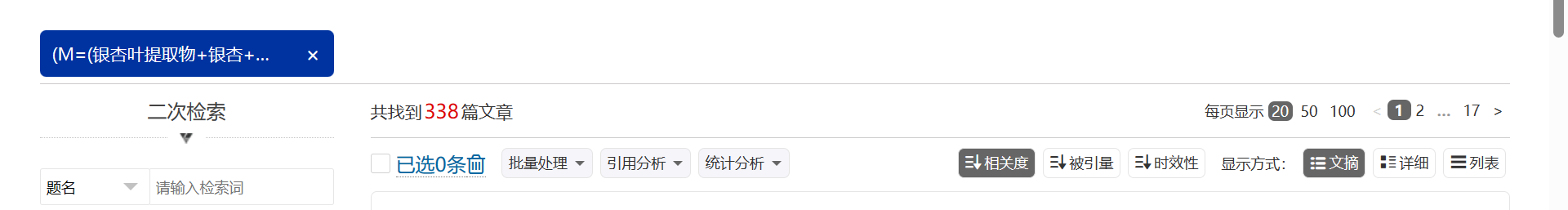


Wan Fang

The retrieval of the Wan Fang database was conducted on July 21, 2023, and a total of 404 records were retrieved.

(主题:(“银杏叶提取物” or “银杏” or “银杏叶” or “白果叶” or “金纳多” or “达纳康” or “依康宁”) or 题名或关键词:(“银杏叶提取物” or “银杏” or “银杏叶” or “白果叶” or “金纳多” or “达纳康” or “依康宁”)) and (主题:(“糖尿病肾病” or “糖尿病性肾病” or “糖尿病肾脏病” or “糖尿病性肾脏病” or “糖尿病肾脏疾病” or “糖尿病性肾脏疾病” or “消渴肾病” or “消渴肾衰” or “消渴肾风”) or 题名或关键词:(“糖尿病肾病” or “糖尿病性肾病” or “糖尿病肾脏病” or “糖尿病性肾脏病” or “糖尿病肾脏疾病” or “糖尿病性肾脏疾病” or “消渴肾病” or “消渴肾衰” or “消渴肾风”))


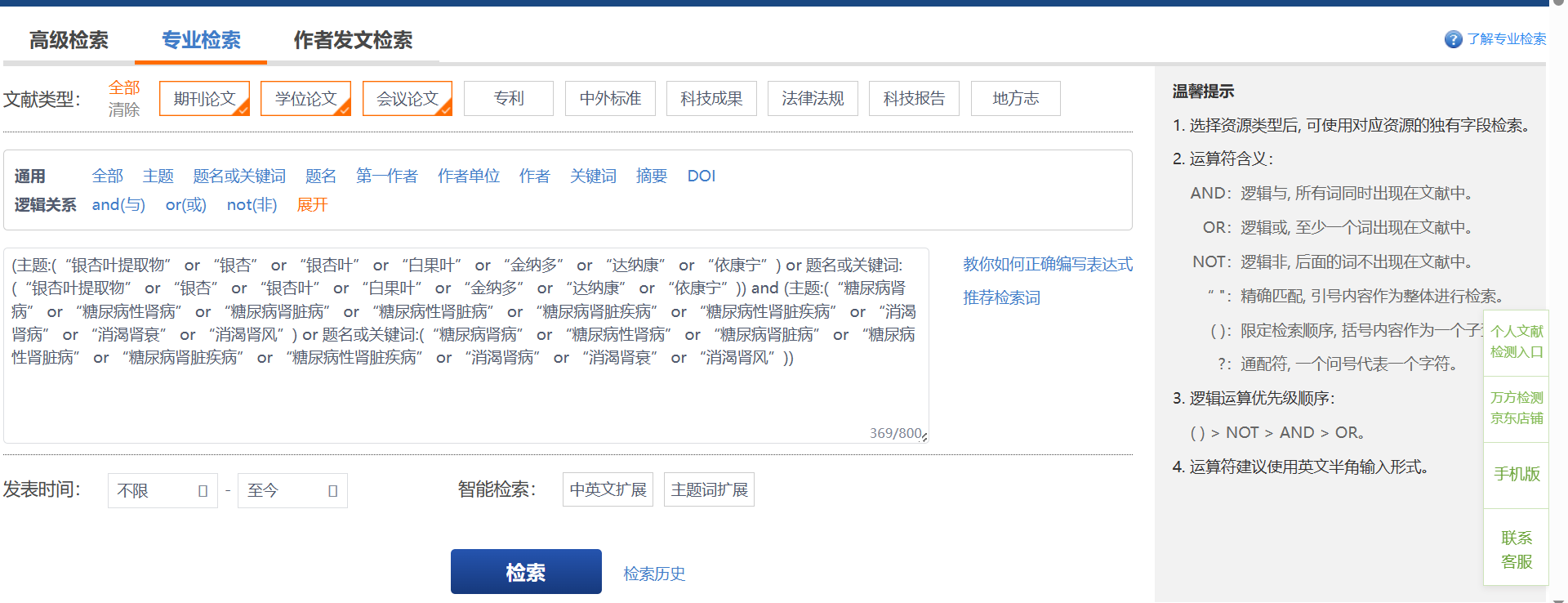


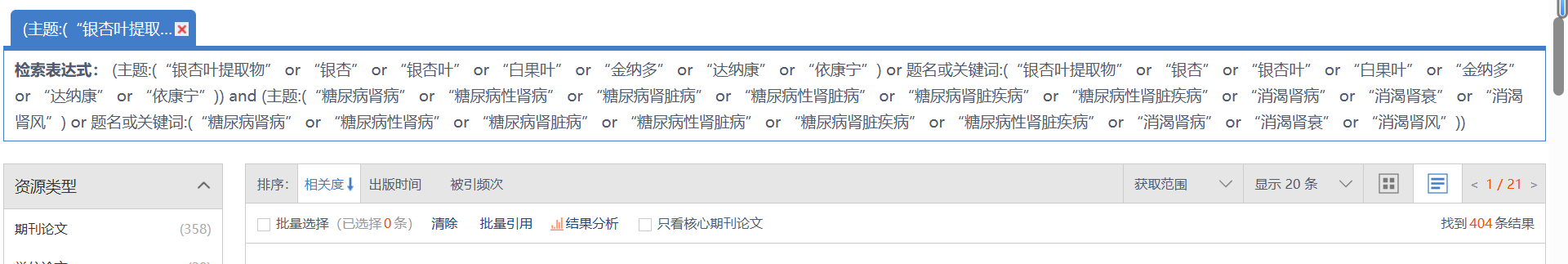


CBM

The retrieval of the CBM database was conducted on July 21, 2023, and a total of 368 records were retrieved.


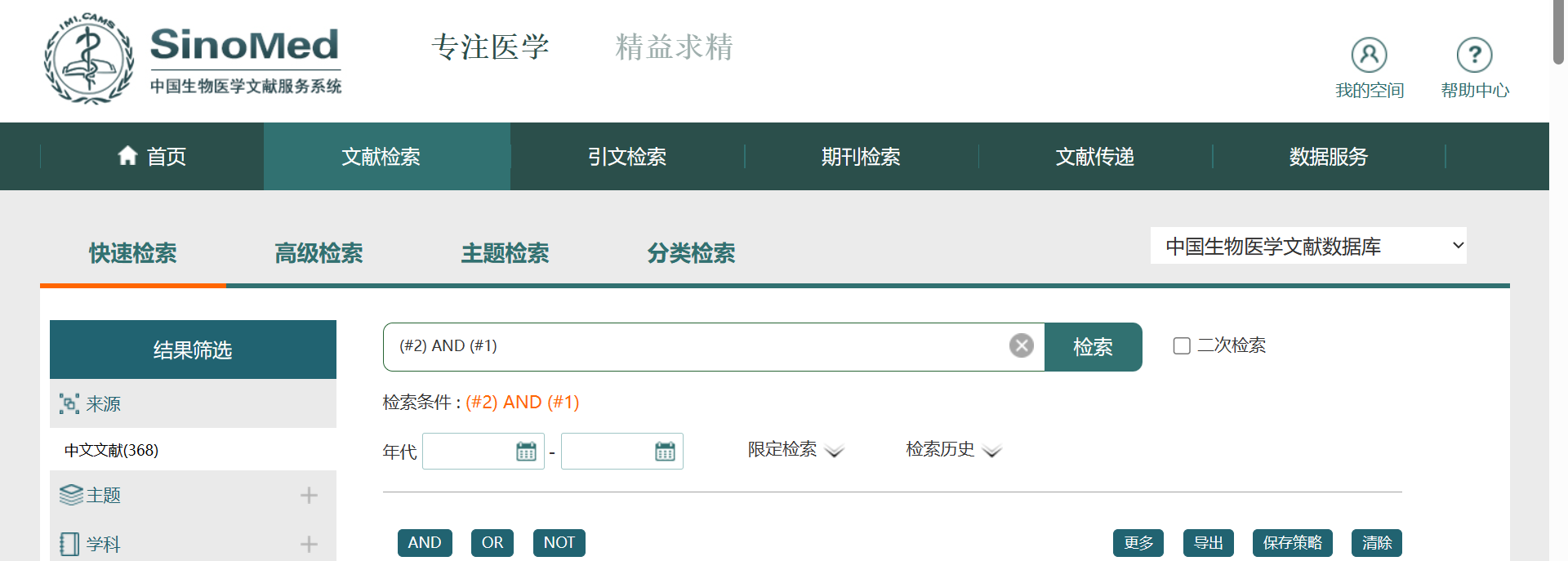


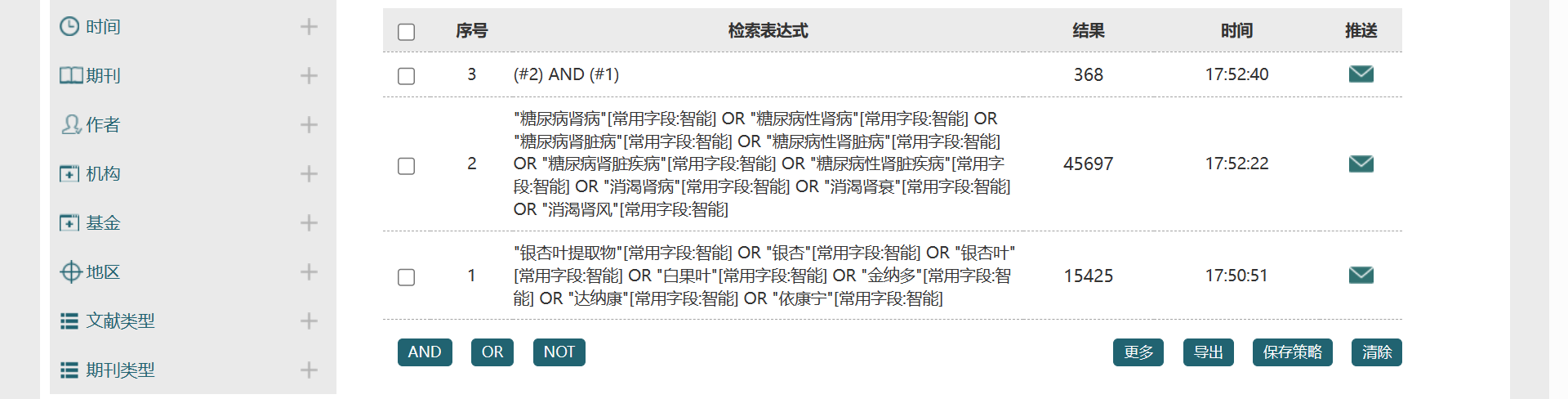


ClinicalTrials.gov

The retrieval of the ClinicalTrials.gov database was conducted on July 21, 2023, and no records were retrieved.


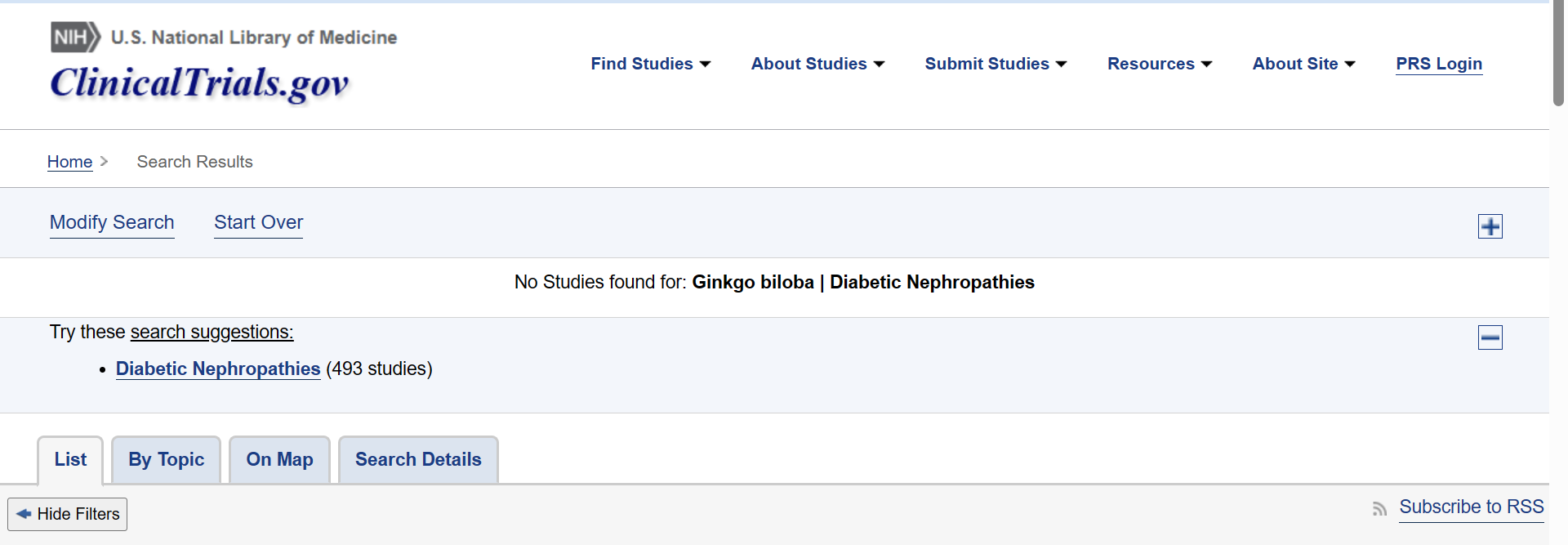


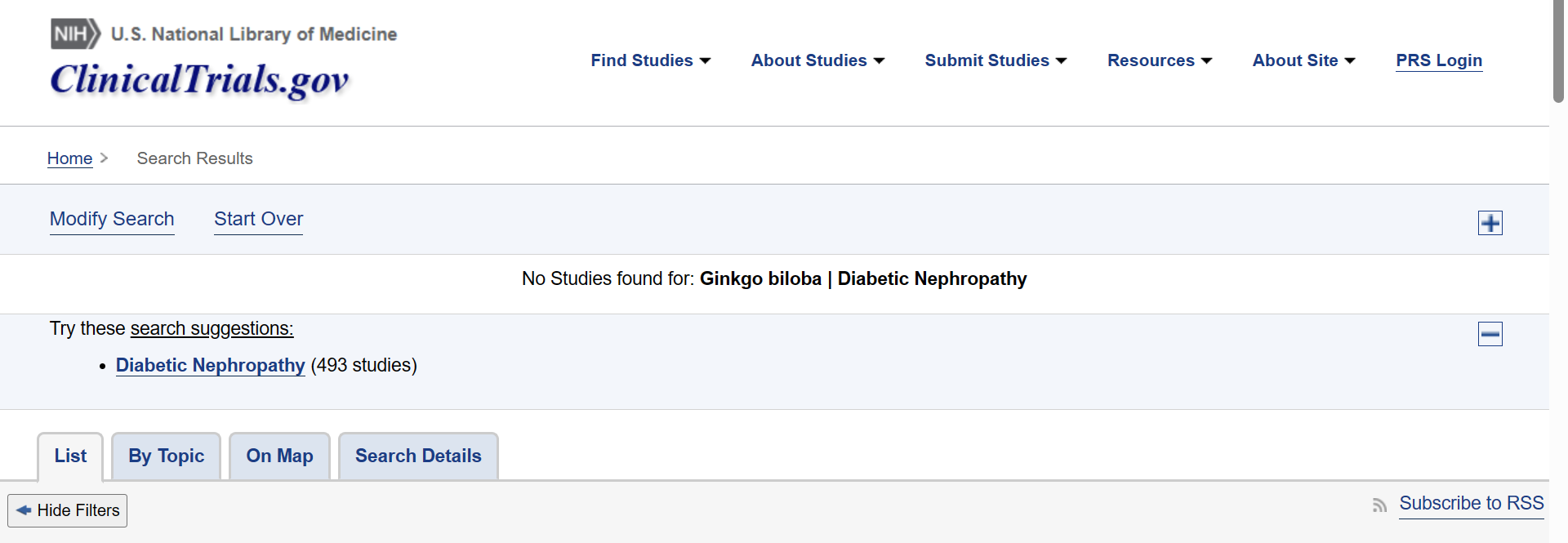


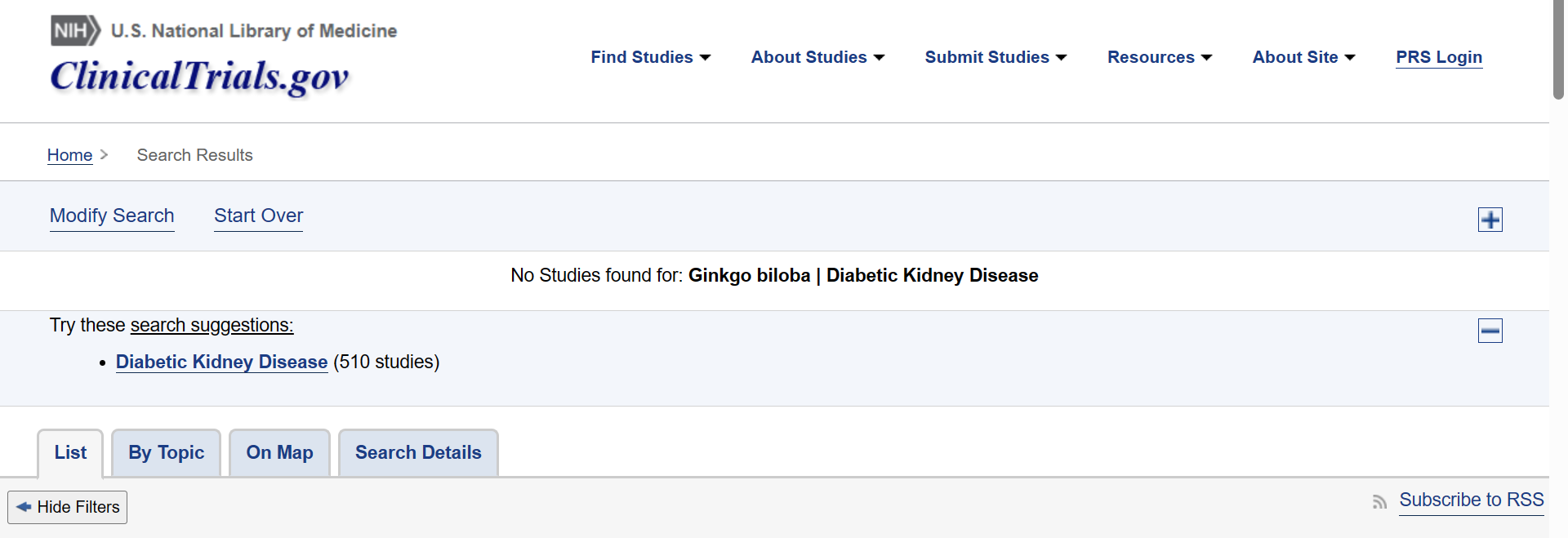


Chinese Clinical Trial Registry

The retrieval of the Chinese Clinical Trial Registry was conducted on July 21, 2023, and no records were retrieved.


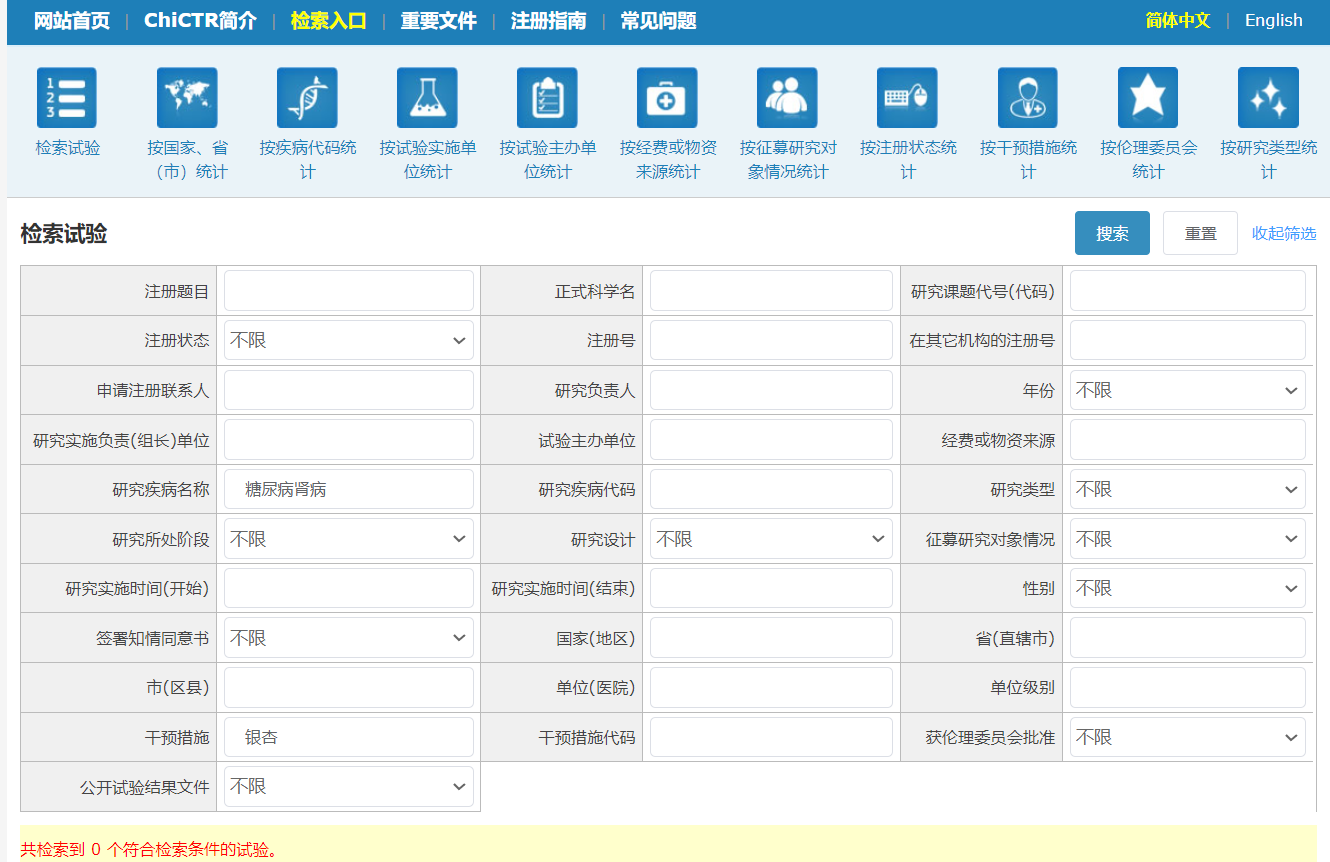


# Supplementary Material S3. Literature excluded after reading the full text and reasons

**1) The study design was not a randomized controlled trial:**

[1] Gao, F.R., Zhao, P., Mei, X.L., 2002. Observation on the Efficacy of Losartan Combined with Ginaton in the Treatment of Elderly Diabetic Nephropathy. Inn. Mong. Med. J. 34 (5), 407-408. (The study was a single-arm trial with no control group.)

[2] Qadir, A., 2016. Study on the Efficacy of Irbesartan Tablets Combined with Ginkgo and Damo Injection in the Treatment of Diabetic Nephropathy. Diabetes New World 19 (6), 6-7. (The subjects in this study were not randomly assigned to each group.)

[3] Han, A.Q., Fang, H.W., Du, P.F., 2013. Clinical Observation on the Adjuvant Treatment of Diabetic Nephropathy with Ginkgo Damo Injection. Zhejiang J. Integr. Tradit. Chin. West. Med. 23 (4), 310-311. (The study was a single-arm trial with no control group.)

[4] Ye, J.R., 2012. The Research of the Yinxingdamo Injection in the Comprehensive Efficacy of Early Diabetic Nephropathy. Chin. J. Med. Guide 14 (12), 2140-2141. (This was a retrospective study.)

[5] Li, S.H., 2012. Study on the Intervention of Ginkgo Damo Injection on Renal Hemodynamics in Patients with Type 2 Diabetic Nephropathy. Chin. J. Crit. Care Med., Electron. Ed. 5 (6), 400-402. (The study was a single-arm trial with no control group.)

[6] Jiang, L.Y., 2012. Observation on the Clinical Efficacy of Ginkgo Damo Injection in the Treatment of 60 Patients with Stage Iii Diabetic Nephropathy. Shanxi Med. J. 41 (4), 397. (The subjects in this study were not randomly assigned to each group.)

[7] Chen, A.M., 2012. Clinical Efficacy of Enalapril Combined with Ginkgo Biloba Capsules in the Treatment of 50 Patients with Early Diabetic Nephropathy. Acad. J. Guangzhou Med. Univ. 40 (6), 79-80. (The study was a single-arm trial with no control group.)

[8] Li, G.X., Jiang, L., Liu, Y., 2004. Effects of Xingding Injection on Hemorrheology in Patients with Diabetic Nephropathy. Shandong Med. J. 44 (31), 25. (The study was a single-arm trial with no control group.)

[9] Zhao, P., 2007. Analysis of 41 Patients with Type 2 Diabetic Nephropathy Treated by Ginaton Injection Combined with Losartan. Chin. J. Misdiagn. 7 (21), 5127-5128. (The study was a single-arm trial with no control group.)

[10] Gu, Y.J., 2011. Ginkgo Leaf Injection Matched with Losartan in Treating Diabetic Nephropathy. China Mod. Med. 18 (34), 97+99. (This was a retrospective study.)

[11] Chen, C.Q., Jiang, G.B., 2012. Observation on the Efficacy of 48 Cases of Early Diabetic Nephropathy Treated with Ginkgo Biloba Combined with Enalapril. China Rural Health (z1), 244-245. (This was a case report.)

[12] Kong, X.M., 2018. Effect of Ginkgo Damo Injection Combined with Irbesartan on Early Diabetic Nephropathy. Health Everyone (12), 63. (This was a case report.)

[13] Yan, X., Wang, W.M., 2012. Observation on the Efficacy of Ginkgo Leaf Extract and Dipyridamole Injection Combined with Irbesartan in the Treatment of Early Diabetic Nephropathy. Chin. J. Clin. Ration. Drug Use 5 (21), 70-71. (The subjects in this study were not randomly assigned to each group.)

[14] Zhou, X.Y., 2011. Observation on the Efficacy of Ginkgo Damo Injection Combined with Benazepril in the Treatment of Early Diabetic Nephropathy. World Health Dig. 8 (22), 85-86. (The subjects in this study were not randomly assigned to each group.)

[15] Xu, Y.G., Gao, F., Li, F., 2010. Clinical Observation of Ginkgo Leaf Extract and Dipyridmoie Injection and Fosinopril in Treatment of Diabetic Nephropathy. Med. J. Liaoning 24 (6), 289-291. (This was a case report.)

[16] Yang, C., Cui, X.G., Lv, F.X., 2008. Observation on 79 Cases of Diabetic Nephropathy Treated by Ginaton Combined with Ligustrazine. Chin. Community Doct. 10 (19), 107-108. (The study was a single-arm trial with no control group.)

**2) The patients had acute infection:**

[1] Qiang, X.Z., Lin, J.S., Huang, H., 2017. Retrospective Analysis of the Clinical Efficacy and Adverse Reactions of Enalapril Combined with Ginkgo Biloba Capsules in the Treatment of Early Diabetic Nephropathy. Diabetes New World 20 (20), 170-171.

[2] Wang, G.C., 2012. Study on the Efficacy of Valsartan Combined with Ginkgo and Damo Injection in Patients with Early Diabetic Nephropathy. China Prac. Med. 7 (25), 153-154.

**3) The treatment group did not combine ACEI/ARB:**

[1] Li, W.H., Jin, Y.H., Li, C.Y., 2003. Observation on the Curative Effect of Ginaton in the Treatment of Early Diabetic Nephropathy. J. Mod. Med. Health 19 (6), 747-748.

[2] Quan, Q.F., 2003. Clinical Observation of Ginkgo Damo Injection in the Treatment of Early Diabetic Nephropathy. Shaanxi Med. J. 32 (9), 829-830.

[3] Jin, M.H., Pan, L., Wang, Y., Sun, Y.Q., Feng, D.W., Zhang, X.L., Hou, L.Q., Chai, G.L., 2016. Effect of Shuxuening on Serum Visfatin and Related Items in Patients with Diabetic Nephropathy. Chin. J. Integr. Tradit. West. Nephrol. 17 (12), 1100-1102.

[4] Xiang, Q., Song, E.F., Mei, S.S., 2014. Effect of Ginkgo Extract Combined with Sulodexide in Diabetic Nephroathy Ⅲ Patients. Chin. J. Microcirc. 24 (1), 27-29+26+29.

[5] Wang, Z.Y., Yang, L.Z., 2013. Effect of Ginkgo Damo Injection Combined with Alprostadil Injection on Adiponectin in Patients with Diabetic Nephropathy (Ⅲ-Ⅳ Stage). For All Health 7 (24), 33.

[6] Li, B., Bi, C.L., 2013. Effect of Ginkgo Leaf Extract and Dipyridamole Injection on Oxidative Stress Level of Patients with Diabetic Nephropathy. Acta Chin. Med. 28 (9), 1354-1355.

[7] Zhu, J.B., 2012. Clinical Observation on Ginkgo Biloba Combined with Conventional Western Medicine in the Treatment of Diabetic Nephropathy. Acta Chin. Med. Pharmacol. 40 (2), 122-124.

[8] Liu, L., 2012. Effect of Shuxuening on Oxidative Stress in Patients with Early Diabetic Nephropathy. Chin. J. Integr. Tradit. West. Nephrol. 13 (7), 638-639.

[9] Hao, G.H., Zhao, T., Chen, D., 2012. Study on the Effect of Ginkgo Damo Injection on Renal Artery Hemodynamics in Diabetic Nephropathy. Guide China Med. 10 (16), 283.

[10] Zhang, X.G., 2011. Effect of Ginkgo Damo Injection on Tnf-Α and Il-6 in Patients with Early Diabetic Nephropathy. Chin. J. Aesthetic Med. 20 (z5), 301.

[11] Wang, D.F., Sun, L., Zhang, L.Q., Liu, B.G., Bai, B.Y., Guo, S.L., 2005. Study on the Effect of Ginkgo Flavonoid Glycosides on Plasma Plasminogen Activator Inhibitor-1 in Patients with Early Diabetic Nephropathy. Clin. Focus 20 (15), 882-883.

[12] Lu, J., He, H., 2005. Effect of Ginkgo Biloba Extract Injection on Urinary Microalbumin of Early Diabetic Nephropathy. Clin. Focus 20 (12), 673-675.

[13] Lu, J., He, H., 2005. Clinical Observation of Gingko Biloba Extract Injection in Treating Early Diabetic Nephropathy. Chin. J. Integr. Med. 11 (3), 226-228.

[14] Huang, J.Z., Qiu, X.P., Wang, J., 2006. Plasma Level of Circulating Endothelial Cells in Patients with Early Diabetic Nephropathy and Interventional Treatment of Ginkgo-Damole Injection. Chin. J. Clin. Pharmacol. Ther. 11 (11), 1309-1312.

[15] Xu, X.M., Song, L.L., Guo, X.Y., 2006. Observation on the Efficacy of Ginkgo Leaf Extract in the Treatment of Early Diabetic Nephropathy. Chin. J. Clin. Med. Res. (160), 27.

[16] Chen, L.G., 2007. Clinical Observation of Ginkgo Leaf Extract and Dipyridamole Injection in the Treatment of Early Diabetic Nephropathy. Chin. J. Inf. Tradit. Chin. Med. 14 (12), 75-76.

[17] Guo, Y.X., Lv, D.M., Cao, S.H., Wang, J.L., 2007. Ginkgo Leaf Extract Combined with Gliclazide and Metformin in the Treatment of 32 Patients with Early Diabetic Nephropathy. Chin. J. Integr. Tradit. West. Nephrol. 8 (10), 607-608.

[18] Tu, Y.B., Liu, X.L., Shu, Y.B., Li, C.P., 2010. Observation on the Efficacy of Ginkgo Leaf Preparations in Treating Stage Ⅲ~Ⅵ Diabetic Nephropathy. Clin. J. Tradit. Chin. Med. 22 (8), 674-675.

[19] Zhang, J.W., 2007. Effect of Xingding on Hemorheology of Early Diabetic Nephropathy. Shanxi Med. J. 36 (1), 74-75.

[20] Zhang, L., Fang, X.M., Song, X.Y., 2007. Effect of Ginkgo Biloba Extract Injection on Urinary Microalbumin Excretion in Patients with Early Diabetic Nephropathy. China Pharm. 18 (30), 2374-2375.

[21] Chai, G.L., Yang, Y.H., Pang, J.W., Pan, J.Q., Tan, L.Y., 2008. Effects of Shuxuening on Plasma Et, Angⅱ, Adm and No Levels in Patients with Diabetic Nephropathy. Chin. J. Integr. Tradit. West. Nephrol. 9 (5), 445-447.

[22] Li, X.S., Lang, X.J., Fu, X.J., Ye, S.H., Chen, Y.H., 2008. Effect of Extract Gingko Biloba on Tgf-Β1 and Ctgf in Patients with Early Diabetic Nephropathy. Chin. J. Gerontol. 28 (6), 580-582.

[23] Liu, Y.C., Luo, S.W., Zhu, Y., 2008. Observation on the Clinical Efficacy of Ginkgo Damo Injection in the Treatment of Diabetic Nephropathy. Mod. J. Integr. Tradit. Chin. West. Med. 17 (21), 3260-3261.

[24] Wang, W.Y., Zhang, J.S., Wang, Z.X., 2008. Effect of Ginkgo Biloba Injection on Urinary Microalbumin in Patients with Diabetic Nephropathy. Zhejiang J. Integr. Tradit. Chin. West. Med. 18 (11), 705.

[25] Li, X.S., Zheng, W.Y., Lou, S.X., Lu, X.W., Ye, S.H., 2009. Effect of Ginkgo Leaf Extract on Vascular Endothelial Function in Patients with Early Stage Diabetic Nephropathy. Chin. J. Integr. Med. 15 (1), 26-29.

[26] Chen, L.B., Zhang, C.N., Gu, J.F., Zhao, H.Y., Han, J., Cui, Y.F., 2009. Influence of Ginkgo Leaf Extract and Dipyridamole Injection in Diabetic Nephropathy. J. Med. Res. 38 (11), 67-68.

[27] Wu, L.H., Liao, Y.F., Zhang, X.R., 2009. Effect of Ginkgo Damo Injection on Thrombomodulin in Patients with Diabetic Nephropathy. Zhejiang Pract. Med. 14 (4), 297-298.

[28] Zhang, G., Xu, L.L., Wang, H.F., Yuan, L.P., Yan, J.J., 2009. Observation on the Efficacy of Ginkgo Leaf Extract in the Treatment of Early Diabetic Nephropathy. Mod. J. Integr. Tradit. Chin. West. Med. 18 (20), 2400-2401.

[29] Zhong, C.F., 2009. Observation on the Efficacy of Ginkgo Damo Injection in the Treatment of Early Diabetic Nephropathy. Natl. Med. Front. China 4 (6), 15.

**4) Treatments other than prescribed were used:**

[1] Wang, Y.P., 2014. Molecular Mechanism of Ginkgo Biloba on Advanced Glycosylation End Products (Ages) Mediated Diabetic Nephropathy [Doctoral thesis]. Nanjing: Southeast University. (Treatment was combined with Liuwei Dihuang Pills.)

[2] Zhai, W.J., Li, A.Q., 2006. Observation on 68 Patients with Early Diabetic Nephropathy Treated with Integrated Traditional Chinese and Western Medicine. J. Med. Res. 35 (9), 106. (Treatment was combined with enemas.)

[3] Zhou, Y.Y., Zhang, X.M., Si, F.X., Tian, Y.Q., 2007. Clinical Observation on the Treatment of Early Diabetic Nephropathy with Ginkgo Leaf Extract. Chronic Pathematology J. (9), 143. (The control group used Salvia miltiorrhiza injection.)

[4] Ning, Y.C., Zhang, Y.H., 2009. Shuxuening Combined with Western Medicine to Treat 33 Cases of Early Diabetic Nephropathy. Shaanxi J. Tradit. Chin. Med. 30 (4), 403-404. (The control group used Salvia miltiorrhiza injection.)

**5) The control measure did not meet the standard:**

[1] Feng, L.Y., 2013. Clinical Observation on Ginkgo Damo Injection Combined with Perindopril in the Treatment of Diabetic Nephropathy. J. Front. Med. (31), 204-205. (The control group did not use ACEI/ARB.)

[2] Wang, Y.H., Zhang, X.Y., Zhang, S.Q., Luan, Y., 2012. Effects of Ginkgo Leaf Extract and Dipyridamole Combined with Telmisartan on Serum Cystatin C in Diabetic Nephropathy. China Pharm. 21 (A02), 25. (The control group did not use ACEI/ARB.)

[3] Yang, F., Liu, M.Y., 2011. Clinical Observation on Benazepril Combined with Ginkgodamole in the Treatment of Diabetic Nephropathy. J. Pract. Diabetol. 7 (6), 24. (The control group did not use ACEI/ARB.)

[4] Li, C.X., 2011. Clinical Observation on 38 Cases of Diabetic Nephropathy Treated with Ginkgo Damo. Jilin Med. J. 32 (19), 3946. (The control group did not use ACEI/ARB.)

[5] Kong, Q., 2006. Efficacy of Lisinopril Combined with Xingding in the Treatment of Early Diabetic Nephropathy. J. Mod. Med. Health 22 (10), 1459-1460. (The control group did not use ACEI/ARB.)

[6] Liang, C.S., 2006. Clinical Observation on Shuxuening Injection in the Treatment of Diabetic Nephropathy. Med. Forum 10 (10), 903-904. (The control group did not all use ACEI/ARB.)

[7] Yang, H., 2008. Ginkgo Damol Combined with Angiotensin Converting Enzyme Inhibitor in the Treatment of Diabetic Nephropathy. Hainan Med. J. 19 (11), 41+29. (The control group did not use ACEI/ARB.)

[8] Gao, Y.B., 2009. Observation on the Efficacy of Irbesartan Combined with Ginkgodamole in the Treatment of Diabetic Nephropathy. Chin. J. Mod. Drug Appl. 3 (20), 121-122. (The ACEI/ARB preparations used by the two groups were inconsistent.)

[9] Liao, X.L., 2009. The Clinical Observation of Benazepril Combined with Ginkgo Leaf Extract and Dipyridamole Injection on Early Diabetic Nephropathy. West China Med. J. 24 (3), 673-675. (The control group did not use ACEI/ARB.)

[10] Ye, P.S., Zhou, W.L., 2009. Effects of Ginkgo Biloba Leaves on Insulin Resistance and Adipocytokines in Diabetic Nephropathy. Zhejiang J. Tradit. Chin. Med. 44 (12), 876-877. (The control group did not all use ACEI/ARB.)

[11] Shi, D., Kong, D.M., Xu, H.S., Chen, Y.H., 2016. Effect of Jiawei Sier Decoction on Ual, Sod and Mda Levels in Patients with Early Type 2 Diabetic Nephropathy. Chin. J. Exp. Tradit. Med. Formulae 22 (14), 177-181. (The control group did not use ACEI/ARB.)

[12] Shi, D., 2015. Clinical Study on the Therapeutic Effect of Jiawei Sier Decoction on Early Diabetic Nephropathy [Master's thesis]. Guiyang: Guizhou University of Traditional Chinese Medicine. (The control group did not use ACEI/ARB.)

[13] He, W., 2009. The Clinical Observation in the Yinxingdamo Injection Treatment of Early Diabetic Nephropathy Urine Microalbumin [Master's thesis]. Wuhan: Hubei University of Chinese Medicine. (The control group did not all use ACEI/ARB.)

[14] Chen, Y., Wu, X.Q., 2015. Observation on the Efficacy of Candesartan Combined with Ginkgo Damo Injection in the Treatment of Early Diabetic Nephropathy. Mod. J. Integr. Tradit. Chin. West. Med. 24 (1), 44-46. (The ACEI/ARB preparations used by the two groups were inconsistent.)

[15] Peng, L.M., Xia, X.F., Zhao, Z.H., 2013. Clinical Observation of Ginkgo Leaf Extract and Dipyridamole Injection in Treating Diabetic Nephropathy. Cardiovasc. Cerebrovasc. Dis. Prev. Treat. 13 (2), 157-158. (The control group did not all use ACEI/ARB.)

[16] Sun, L.P., Liu, H.X., Lu, H.W., Wang, X.J., Liu, C.S., 2009. Ginkgo-Dipyridamolum Injection for Treatment of Early Diabetic Nepbropathy. China Med. 4 (6), 437-438. (The control group did not all use ACEI/ARB.)

**6) Incomplete reporting of intervention features:**

[1] Zhu, M., 2020. Clinical Evaluation of Ginkgo Damo Injection Combined with Candesartan in the Treatment of Early Diabetic Nephropathy. Women's Health Res. (9), 50+96. (The course of treatment was not reported.)

[2] Yang, X.M., 2014. Observation on Curative Effect of Enalapril Combined with Ginkgo Dipyridamole Injection on Early Diabetic Nephropathy. Clin. J. Chin. Med. 6 (21), 66-67. (The frequency of Ginkgo biloba extract was not reported.)

[3] Zhuang, G., 2009. The Clinical Observation of Xingding Injection in Treating Early Diabetic Nephropathy [Master's thesis]. Wuhan: Hubei University of Chinese Medicine. (The frequency of ACEI was not reported.)

[4] Tian, Y.F., Liu, Y.M., Feng, X., 2009. Therapeutic Effect of Ginkgo Damo Injection on Microalbuminuria in Early Diabetic Nephropathy. China Foreign Med. J. 7 (5), 69-70. (The type, usage, dosage and duration of ACEI/ARB was not clearly stated.)

[5] Mao, C.P., Li, X.Y., Zhang, H.M., Lin, G.F., 2009. Clinical Study on the Treatment of Early Diabetic Nephropathy with Ginkgo Leaf Extract. Clin. Med. China 25 (3), 299-301. (The dosage of irbesartan was not clearly stated.)

[6] Mao, C.P., Li, X.Y., Zhang, H.M., Cai, W.T., 2009. Effect of Extract of Gingko Biloba on Oxidative Stress in Early Diabetic Nephropathy. Shandong Med. J. 49 (37), 13-15. (The dosage of irbesartan was not clearly stated.)

[7] Li, X.Y., Mao, C.P., 2009. Clinical Study of Ginkgo Leaf Extract Combined with Irbesartan in the Treatment of Early Diabetic Nephropathy. Suzhou Univ. J. Med. Sci. 29 (4), 715-716+726. (The dosage of irbesartan was not clearly stated.)

[8] Shen, X.M., Jiang, Y., Xu, X.G., 2010. Therapeutic Effect of Ginkgo Biloba Leaf in the Adjuvant Treatment of Early Diabetic Nephropathy. J. Chin. Physician 12 (5), 696-697. (The type, usage and dosage of ACEI/ARB was not clearly stated.)

[9] Chen, H.J., Yu, Y., Lin, H.C., Sun, X.L., 2006. Effect of Ginkgo Biloba Extract in the Adjuvant Treatment of Early Diabetic Nephropathy. Guangdong Med. J. 27 (2), 235-236. (The type, usage and dosage of ACEI/ARB was not clearly stated.)

[10] Liu, H.H., Liu, D.J., Tian, S.J., 2011. Effect of Ginkgo Biloba Extract Capsules on Level of Plasma Brain Natriuretic Peptide and Cardiovascular Event in Diabetic Nephropathy Patients. Chin. J. Postgrad. Med. 34 (25), 7-10. (The type, usage and dosage of ACEI/ARB was not clearly stated.)

[11] Xiong, Y., Zhao, X.M., Chen, M., Shen, J.M., Wang, L.P., 2011. Effects of Ginkgo Leaf Extract and Dipyridamole Injection on Oxidative Stress in Patients with Early Diabetic Nephropathy. J. Hubei Univ. Med. 30 (2), 169-171. (The dosage and frequency of ACEI/ARB dosage was not clearly stated.)

[12] Zhang, P.K., Wang, F., 2011. Efficacy Observation of Diabetic Nephropathy Treated with Ginkgo-Damole Injection. World J. Integr. Tradit. West. Med. 6 (1), 54-55. (The type, usage and dosage of ACEI/ARB was not clearly stated.)

[13] Fu, D.J., Dong, A.W., Zheng, J., Li, J.X., Lan, H., Li, W.B., Cao, L.L., 2007. Clinical Observation of Valsartan Combined with Ginkgo Damol in the Treatment of Early Type 2 Diabetic Nephropathy. J. Gannan Med. Univ. 27 (3), 378-379. (The frequency of ACEI/ARB and Ginkgo biloba extract was not clearly stated.)

**7) Duplicate literature:**

[1] Wang, X.J., 2012. Observation on the Efficacy of Ginkgo Damo Injection Combined with Irbesartan in the Treatment of Early Diabetic Nephropathy. J. Pract. Tradit. Chin. Med. 28 (2), 109-110.

[2] Li, H.M., Li, S.L., Lu, S.R., 2009. Clinical Observation on the Therapeutic Effect of Benazepril and Ginkgo-Damole Injection in Treating Patients with Early Diabetic Nephropathy. Mod. Med. J. China 11 (2), 50-52.

**8) Lack of sufficient data results:**

[1] Wang, H.P., Wang, L.P., Wang, L.J., 2009. Clinical Observation of Valsartan Combined with Ginkgo Damol in the Treatment of Early Diabetic Nephropathy. For All Health (4), 63.

**9) Lack of required indicators:**

[1] Yu, Y.F., Lin, C.X., 2009. Clinical Observation of 32 Cases of Early Diabetic Nephropathy Treated with Ginkgo Biloba Extract. Strait Pharm. J. 21 (2), 95.

[2] Li, C.Y., 2014. Clinical Observation on Treating Diabetic Nephropathy with the Yinxing Damo Injection. Clin. J. Chin. Med. 6 (4), 97-98.

[3] Xu, W.L., Yang, Q.F., Lin, X.Y., Luo, E.S., 2011. Efficacy Evaluation of Ginkgo Damol Injection Combined with Benazepril in the Treatment of Diabetic Nephropathy. China Prac. Med. 6 (24), 177-178.

[4] Guo, X.H., Li, F.S., Wang, L., 2007. Observation on the Treatment of Early Diabetic Nephropathy with Ginkgo Biloba Injection Combined with Telmisartan. Chin. Community Doct. 9 (23), 55.

**10) There were obvious errors:**

[1] Wang, J., Ping, X.Y., 2009. Observation on the Efficacy of Ginkgo Leaf Extract Combined with Irbesartan in the Treatment of Early Diabetic Nephropathy. Shanxi Med. J. 38 (10), 942-943. (The sample size was inconsistent and contradictory in the context before and after.)

[2] Xiong, L., Xiao, Y.Y., 2012. Clinical Observation and Nursing Care of Valsartan Combined with Ginkgo and Damole in the Treatment of Early Diabetic Nephropathy. Chin. J. Misdiagn. 12 (18), 4944-4945. (The sample size was inconsistent and contradictory in the context before and after.)

[3] Wang, X.S., 2010. Observation on the Clinical Efficacy of Ginkgo Damo Injection on Type 2 Diabetic Nephropathy. J. Pract. Diabetol. 6 (5), 40-41. (Incorrect use of statistical method.)

[4] Zhang, S.J., Chen, J., 2010. Clinical Analysis of Ginkgo Biloba Combined with Captopril in the Treatment of Early Diabetic Nephropathy. China Mod. Med. 17 (27), 80-81. (Incorrect use of statistical method.)

[5] Wu, P.L., Liu, R., 2010. Therapeutic Effect of Ginkgo Damol Combined with Enalapril on 26 Cases of Diabetic Nephropathy Proteinuria. J. Xianning Univ. 24 (2), 121-122. (Incorrect use of statistical method.)

[6] Yan, C.Y., Chen, S.Z., 2008. Therapeutic Effect of Ginkgodamol Combined with Valsartan in the Treatment of Diabetic Nephropathy. Mod. Health Med. Innovation Res. 5 (27), 141-142. (The sample size was inconsistent and contradictory in the context before and after.)

[7] Li, L., 2012. Effects of Ginkgo Biloba Extract and Dipyridamole Injection Combined with Valsartan in the Treatment of Early Diabetic Nephropathy. Anhui Med. J. 33 (3), 334-335. (The sample size was inconsistent and contradictory in the context before and after.)

[8] Li, Q., Han, L.Y., 2010. Clinical Observation on Treatment of Early Diabetic Nephropathy with Ginaton Combined with Fosinopril. Chin. J. Misdiagn. 10 (15), 3572. (The sample size was inconsistent and contradictory in the context before and after.)

**11) Data authenticity was questionable:**[1] Wang, H.Y., Hu, J.T., Zheng, G.C., 2014. Observation on the Efficacy of Ginkgo Damo Injection Combined with Irbesartan in the Treatment of Early Diabetic Nephropathy. J. Med. Inf. 27 (9), 267-268. (There were obvious errors in the data.)

[2] Chen, J.S., 2015. Observation on the Efficacy of Valsartan Combined with Ginkgo Damo Injection in the Treatment of Patients with Early Diabetic Nephropathy. Chin. J. Pharm. Econ. 10 (9), 34-35. (There were obvious errors in the data.)

**Final Included References:**

[1] Chen, F., Ma, Y.L., Chen, B.P., 2010. Effect of Shuxuening on Il-18 in Patients with Early Diabetic Nephropathy. China Pharm. 21 (28), 2645-2647.

[2] Cheng, H., Su, K., Chen, C., Liang, W., 2018. Efficacy of Ginkgo Leaf Extract and Dipyridamole Injection Combined with Telmisartan for Early Diabetic Nephropathy. Guangxi Med. J. 40 (24), 2921-2924.

[3] Chu, L., 2010. The Effect of Valsartan Combined with Ginkgo Biloba Extract in the Treatment of Early Diabetic Nephropathy. Chin. J. Postgrad. Med. 33 (7), 16-18.

[4] Fu, Y.Y., Jia, R.H., 2006. Observation on the Therapeutic Effect of Losartan Plus Xingding Injection on Diabetic Nephropathy. J. Pract. Med. 22 (5), 580-581.

[5] Guo, X.C., Xu, X.L., 2015. Clinical Observation on the Treatment of Early Diabetic Nephropathy with Ginkgo Leaf Extract and Dipyridamole Injection Combined with Irbesartan. New Chin. Med. 47 (7), 107-109.

[6] Han, Y.D., 2008. Treatment of 35 Cases of Early Diabetic Nephropathy with Ginkgo Leaf Extract and Dipyridamole Combined with Lotensin. Shaanxi J. Tradit. Chin. Med. 29 (8), 961-962.

[7] Hu, J.Y., 2019. Effects of Ginkgo Leaf Capsules Combined with Enalapril on Inflammatory Factors in Diabetic Nephropathy Patients. Pract. Clin. J. Integr. Tradit. Chin. West. Med. 19 (9), 130-131.

[8] Hu, R.P., Che, H.X., Shi, L., Li, X.X., Wang, M.L., Dong, S.Y., 2016. Observation on the Efficacy of Ginkgo Leaf Extract and Dipyridamole Injection Combined with Imidapril in the Treatment of Early Diabetic Nephropathy. Sci. Technol. Innovation Her. 13 (29), 179-180.

[9] Huang, J.J., 2012. Clinical Analysis of 98 Cases for Treating Diabetic Nephropathy by Losartan Combined with Ginkgo Leaf Extract and Dipyridamole. Chin. J. Med. Guide 14 (10), 1781-1782.

[10] Huang, J.Y., Zhang, F.L., Zhong, H.B., Lin, Y., 2017. Clinical Observation on Ginkgo Leaf Extract and Dipyridamole Injection Combined with Candesartan Cilexetil in Treatment of Early Diabetic Nephropathy. Drugs Clin. 32 (12), 2485-2488.

[11] Jiang, X., Zhu, W.R., Li, J.Y., 2014. Observation on the Clinical Efficacy of Ginkgo Leaf Extract and Dipyridamole Combined with Enalapril in the Treatment of 26 Patients with Early Diabetic Nephropathy. Mod. Diagn. Treat. 25 (15), 3415-3416.

[12] Li, C.X., 2010. Clinical Observation on Shuxuening Injection Combined with Irbesartan in the Treatment of Early Diabetic Nephropathy. Int. J. Urol. Nephrol. 30 (6), 851-853.

[13] Li, H., 2014. Analysis of the Efficacy of Ginkgo Leaf Extract and Dipyridamole Injection Combined with Irbesartan in the Treatment of Early Diabetic Nephropathy. Diabetes New World 34 (7), 19-20.

[14] Li, S.H., 2009. Ginkgo Leaf Extract and Dipyridamole Injection Combined with Fosinopril in the Treatment of Diabetic Nephropathy. Med. Forum 13 (2), 2-3.

[15] Li, Z.Y., 2013. Clinical Observation of Therapy of Integrated Traditional Chinese and Western Medicine on Early Diabetic Nephropathy. Shanxi J. Tradit. Chin. Med. 29 (7), 25-26.

[16] Liang, R.Y., 2008. Observation on the Efficacy of Ginkgo Biloba Injection Combined with Perindopril in the Treatment of 30 Patients with Early Diabetic Nephropathy. Guangxi Med. J. 30 (6), 852-853.

[17] Mao, C.P., Li, X.Y., Zhang, H.M., Cai, W.T., 2010. Extract of Gingko Biloba on Il-6, Tnf-Α in Patients with Early Diabetic Nephropathy. J. Chongqing Med. Univ. 35 (6), 842-844.

[18] Mao, C.P., Zhang, H.M., Zhang, X.L., 2013. Clinical Observation of Ginkgo Biloba Extract on Early Diabetic Nephropathy Patients. J. Pract. Diabetol. 9 (3), 25-26.

[19] Pan, H.J., Huo, G., Kuang, J., 2009. Clinical Observation of Early Diabetic Nephropathy Treated with Temisartan and Ginkgo Biloba Extract Injection. China Clin. Pract. Med. 3 (6), 64-66.

[20] Shen, S.L., 2017. Efficacy of Ginkgo Leaf Extract and Dipyridamole Injection Combined with Benazepril on Serum Cys-C and Β2-Mg Levels in Patients with Early Diabetic Nephropathy. Shaanxi J. Tradit. Chin. Med. 38 (4), 467-468.

[21] Shi, X.H., 2010. Clinical Observation of Benazepril Combined with Ginkgo Leaf Extract and Dipyridamole Injection in the Treatment of 31 Patients with Diabetic Nephropathy. J. Xianning Univ. 24 (6), 492-493.

[22] Sun, C.L., Wang, Z.Q., Liu, G.F., 2009. Clinical Observation on Diabetic Nephropathy Treated with Enalapril and Yinxingdamo Injection. Int. J. Tradit. Chin. Med. 31 (6), 520-521.

[23] Tang, Y., Wu, Y., Liu, Y., Jiang, L., Chen, C., Zhu, X.R., 2013. Clinical Observation on the Efficacy of Ginkgo Biloba Injection Combined with Ramipril on Type 2 Diabetic Nephropathy. Global Tradit. Chin. Med. 6 (S2), 34-35.

[24] Wang, X.W., Zhang, X.L., Xu, Y.H., Wang, D.X., 2010. Observation on the Efficacy of Losartan Combined with Ginkgo Leaf Extract and Dipyridamole in the Treatment of Early Diabetic Nephropathy. Mod. J. Integr. Tradit. Chin. West. Med. 19 (31), 3393-3394.

[25] Wen, Y.H., Wang, X.J., Zhong, X.H., Guo, T.C., 2012. Effect of Ginkgo Leaf Extract and Dipyridamole Injection Combined with Irbesartan on Early Diabetic Nephropathy. Guangdong Med. J. 33 (5), 691-692.

[26] Wu, G.Y., Yan, S.S., Chen, X.B., 2017. The Clinical Efficacy of Treatment of Diabetic Nephropathy with Shuxuening Injection Combined with Benazepril. Chin. J. Biochem. Pharm. 37 (4), 180-182.

[27] Wu, J., Yang, R., Li, X.Y., Yin, Y., Guo, H.L., 2010. Curative Effect of Enalapril Combined with Ginkgo Leaf Extract and Dipyridamole Injection on 36 Patients with Early Diabetic Nephropathy. Sichuan Med. J. 31 (12), 1851-1853.

[28] Xi, H.S., 2011. Therapeutic Effect of Ginkgo Leaf Extract and Dipyridamole Combined with Captopril on Early Diabetic Nephropathy. J. Med. Inf. 24 (3), 1263-1264.

[29] Xiao, Y.Y., 2008. Clinical Observation of Valsartan Combined with Ginkgo Leaf Extract and Dipyridamole in the Treatment of Early Diabetic Nephropathy. J. Clin. Intern. Med. 25 (4), 268-269.

[30] Xing, L.Y., 2022. Analysis of Clinical Effect of Ginkgo Leaf Extract and Dipyridamole Injection Combined with Candesartan Cilexetil in the Treatment of Early Diabetic Nephropathy. China Prac. Med. 17 (23), 145-147.

[31] Xu, Z.H., Dong, M.X., Kong, F.X., Li, S., 2012. Clinical Observation of Ginkgo Leaf Extract and Dipyridamole Combined with Telmisartan in Treatment of Diabetic Nephropathy. Pract. J. Med. Pharm. 29 (3), 209-210.

[32] Xu, Z.H., Dong, M.X., Kong, F.X., Zhou, J.M., 2010. Telmisartan Combined with Ginkgo Leaf Extract and Dipyridamole in the Treatment of 32 Patients with Type 2 Diabetic Nephropathy. Chin. J. Pract. Med. 37 (1), 69-70.

[33] Yang, Q.H., Li, Y.S., Zhang, S.Z., 2007. Observation on the Efficacy of Shuxuening Injection Combined with Benazepril in the Treatment of 35 Patients with Early Diabetic Nephropathy. New Chin. Med. 39 (8), 92-93.

[34] Zhang, C.J., Li, H.M., Shi, F., Song, G.J., 2013. Analysis of the Efficacy of Irbesartan Combined with Ginkgo Dipyridamolum Injection for the Treatment of 48 Patients with Early Diabetic Nephropathy. J. Clin. Res. 30 (5), 850-853.

[35] Zhang, H.S., Zhao, L.J., Peng, B.H., 2009. Effect of Ginkgo Biloba Injection on Urinary Microalbumin in Early Diabetic Nephropathy. Chin. J. Inf. Tradit. Chin. Med. 16 (11), 66.

[36] Zhang, X.G., Zhi, Y.F., Zhang, W., 2009. Observation on the Efficacy of Irbesartan Combined with Ginkgo Leaf Extract and Dipyridamole in the Treatment of Diabetic Nephropathy. Zhejiang Clin. Med. J. 11 (5), 508-509.

[37] Zhang, Y.M., 2015. Clinical Study of Ginkgo Leaf Extract and Dipyridamole Injection Combined with Irbesartan Tablets in the Treatment of Diabetic Nephropathy. Hebei Med. J. 37 (20), 3107-3109.

[38] Zhang, Z.Y., Cao, X., Tan, Z.K., 2014. Enalapril Combined with Yinxingdamo Injection in the Treatment of 100 Patients with Early Diabetic Nephropathy. Mod. Diagn. Treat. 25 (3), 533-534.

[39] Zhen, D., Feng, X.P., 2012. Clinical Observation on Ginkgo Biloba Injection Combined with Telmisartan in the Treatment of Early Diabetic Nephropathy. Tianjin Pharm. 24 (6), 33-35.

[40] Zhu, J., Qiu, C.C., Ye, Y.L., 2020. Observation of Curative Effect of Ginkgo Biloba Injection Combined with Valsartan Capsule on Diabetic Nephropathy. Chin. Foreign Med. Res. 18 (1), 126-127.

[41] Zou, D.X., Zhang, L.M., 2011. Effective Observation on Diabetic Nephropathy Treated by Ginkgo Leaf Extract and Dipyridamole Injection Combined with Irbesartan. J. Mod. Med. Health 27 (12), 1770-1772.

# Supplementary Material S4. Detailed information on GBE formulations according to the ConPhyMP statement

| Study ID (year) | Formulation | Source | Botanical plant name | Plant part used | Origin and harvest time | Specification | Composition and concentration | Quality control reported? (Y/N) | Chemical analysis reported? (Y/N) |
| --- | --- | --- | --- | --- | --- | --- | --- | --- | --- |
| Fu and Jia (2006) | Ginkgo Leaf Extract and Dipyridamole Injection | Guizhou Yibai Pharmaceutical Co., Ltd., China | Ginkgo biloba L | Dry leaves | China. Autumn | 5 mL per vial | Main components: Ginkgo biloba extract 20.5mg (containing Ginkgo flavonoids 4.5-5.5 mg), Dipyridamole 1.8-2.2 mg. Excipients: Glycerin (for injection), Polysorbate 80 (II), Vitamin C. | Y—H52020032, approved by National Medical Products Administration | N |
| Yang et al. (2007) | Shuxuening Injection | HeiLongJiang ZBD Pharmaceutical Co., Ltd., China | Ginkgo biloba L | Dry leaves | China. Autumn | 5 mL per vial | Main components: Ginkgo biloba extract 17.5 mg (containing total flavonol glycosides 4.2 mg, ginkgolides 0.7 mg). Excipients: Ethanol, Sorbitol. | Y—Z23022003, approved by National Medical Products Administration | N |
| Han (2008) | Ginkgo Leaf Extract and Dipyridamole Injection | Shanxi PUDE Pharmaceutical Co., Ltd., China | Ginkgo biloba L | Dry leaves | China. Autumn | 5 mL per vial | Main components: Ginkgo biloba extract 20.5mg (containing Ginkgo flavonoids 4.5-5.5 mg), Dipyridamole 1.8-2.2 mg.Excipients: Vitamin C, Propylene glycol, Polysorbate 80. | Y—H14023515, approved by National Medical Products Administration | N |
| Liang (2008) | Shuxuening Injection | [Commercial Supplier] | Ginkgo biloba L | Dry leaves | China. Autumn | [National Standard] | [National Standard] | Y—Approved by National Medical Products Administration | N |
| Xiao (2008) | Ginkgo Leaf Extract and Dipyridamole Injection | [Commercial Supplier] | Ginkgo biloba L | Dry leaves | China. Autumn | [National Standard] | [National Standard] | Y—Approved by National Medical Products Administration | N |
| Zhang et al. (2009a) | Shuxuening Injection | HeiLongJiang ZBD Pharmaceutical Co., Ltd., China | Ginkgo biloba L | Dry leaves | China. Autumn | 5 ml per vial | Main components: Ginkgo biloba extract 17.5 mg (containing total flavonol glycosides 4.2 mg, ginkgolides 0.7 mg). Excipients: Ethanol, Sorbitol. | Y—Z23022003, approved by National Medical Products Administration | N |
| Pan et al. (2009) | Shuxuening Injection | HeiLongJiang ZBD Pharmaceutical Co., Ltd., China | Ginkgo biloba L | Dry leaves | China. Autumn | 2 ml per vial | Main components: Ginkgo biloba extract 7.0 mg (containing total flavonol glycosides 1.68 mg, ginkgolides 0.28 mg). Excipients: Ethanol, Sorbitol. | Y—Z23022004, approved by National Medical Products Administration | N |
| Li (2009) | Ginkgo Leaf Extract and Dipyridamole Injection | [Commercial Supplier] | Ginkgo biloba L | Dry leaves | China. Autumn | [National Standard] | [National Standard] | Y—Approved by National Medical Products Administration | N |
| Sun et al. (2009) | Ginkgo Leaf Extract and Dipyridamole Injection | Shanxi PUDE Pharmaceutical Co., Ltd., China | Ginkgo biloba L | Dry leaves | China. Autumn | 10 ml per vial | Main components: Ginkgo biloba extract 41.0 mg (containing Ginkgo flavonoids 9.0-11.0 mg), Dipyridamole 3.6-4.4 mg.Excipients: Vitamin C, Propylene glycol, Polysorbate 80. | Y—H14023516, approved by National Medical Products Administration | N |
| Zhang et al. (2009b) | Shuxuening Injection | [Commercial Supplier] | Ginkgo biloba L | Dry leaves | China. Autumn | [National Standard] | [National Standard] | Y—Approved by National Medical Products Administration | N |
| Shi (2010) | Ginkgo Leaf Extract and Dipyridamole Injection | [Commercial Supplier] | Ginkgo biloba L | Dry leaves | China. Autumn | [National Standard] | [National Standard] | Y—Approved by National Medical Products Administration | N |
| Wu et al. (2010) | Ginkgo Leaf Extract and Dipyridamole Injection | [Commercial Supplier] | Ginkgo biloba L | Dry leaves | China. Autumn | [National Standard] | [National Standard] | Y—Approved by National Medical Products Administration | N |
| Chen et al. (2010) | Shuxuening Injection | Langzhi Group Wanrong Pharmaceutical Co., Ltd., China | Ginkgo biloba L | Dry leaves | China. Autumn | 10 ml per vial | Main components: Ginkgo biloba extract 35.0 mg (containing total flavonol glycosides 8.4 mg, ginkgolides 1.4 mg). Excipients: Ethanol, Sorbitol. | Y—Z14021871, approved by National Medical Products Administration | N |
| Mao et al. (2010) | Ginkgo biloba Tablets | Yangtze River Pharmaceutical (Group) Co., Ltd., China | Ginkgo biloba L | Dry leaves | China. Autumn | 0.2 g per tablet | Main components: Ginkgo biloba extract, containing total flavonoid glycosides 9.6 mg and terpene lactones 2.4 mg. Excipients: Starch and others. | Y—Z20027949, approved by National Medical Products Administration | N |
| Xu et al. (2010) | Ginkgo Leaf Extract and Dipyridamole Injection | [Commercial Supplier] | Ginkgo biloba L | Dry leaves | China. Autumn | [National Standard] | [National Standard] | Y—Approved by National Medical Products Administration | N |
| Li (2010) | Shuxuening Injection | HeiLongJiang ZBD Pharmaceutical Co., Ltd., China | Ginkgo biloba L | Dry leaves | China. Autumn | 5 mL per vial | Main components: Ginkgo biloba extract 17.5 mg (containing total flavonol glycosides 4.2 mg, ginkgolides 0.7 mg). Excipients: Ethanol, Sorbitol. | Y—Z23022003, approved by National Medical Products Administration | N |
| Chu (2010) | Shuxuening Injection | [Commercial Supplier] | Ginkgo biloba L | Dry leaves | China. Autumn | [National Standard] | [National Standard] | Y—Approved by National Medical Products Administration | N |
| Wang et al. (2010) | Ginkgo Leaf Extract and Dipyridamole Injection | [Commercial Supplier] | Ginkgo biloba L | Dry leaves | China. Autumn | [National Standard] | [National Standard] | Y—Approved by National Medical Products Administration | N |
| Xi (2011) | Ginkgo Leaf Extract and Dipyridamole Injection | [Commercial Supplier] | Ginkgo biloba L | Dry leaves | China. Autumn | [National Standard] | [National Standard] | Y—Approved by National Medical Products Administration | N |
| Zou and Zhang (2011) | Ginkgo Leaf Extract and Dipyridamole Injection | Hubei Minkang Pharmaceutical Co., Ltd., China | Ginkgo biloba L | Dry leaves | China. Autumn | 10 mL per vial | Main components: Ginkgo biloba extract 41.0 mg (containing Ginkgo flavonoids 9.0-11.0 mg), Dipyridamole 3.6-4.4 mg.Excipients: polysorbate 80, hydrochloric acid, sodium hydroxide, water for injection. | Y—H42022869, approved by National Medical Products Administration | N |
| Wen et al. (2012) | Ginkgo Leaf Extract and Dipyridamole Injection | [Commercial Supplier] | Ginkgo biloba L | Dry leaves | China. Autumn | [National Standard] | [National Standard] | Y—Approved by National Medical Products Administration | N |
| Zhen and Feng (2012) | Shuxuening Injection | [Commercial Supplier] | Ginkgo biloba L | Dry leaves | China. Autumn | [National Standard] | [National Standard] | Y—Approved by National Medical Products Administration | N |
| Xu et al. (2012) | Ginkgo Leaf Extract and Dipyridamole Injection | Guizhou Yibai Pharmaceutical Co., Ltd., China | Ginkgo biloba L | Dry leaves | China. Autumn | 10 mL per vial | Main components: Ginkgo biloba extract 41.0mg (containing Ginkgo flavonoids 9.0-11.0 mg), Dipyridamole 3.6-4.4 mg. Excipients: Glycerin (for injection), Polysorbate 80 (II), Vitamin C. | Y—H52020031, approved by National Medical Products Administration | N |
| Huang (2012) | Ginkgo Leaf Extract and Dipyridamole Injection | [Commercial Supplier] | Ginkgo biloba L | Dry leaves | China. Autumn | [National Standard] | [National Standard] | Y—Approved by National Medical Products Administration | N |
| Li (2013) | Ginkgo Leaf Extract and Dipyridamole Injection | Guizhou Yibai Pharmaceutical Co., Ltd., China | Ginkgo biloba L | Dry leaves | China. Autumn | 10 mL per vial | Main components: Ginkgo biloba extract 41.0mg (containing Ginkgo flavonoids 9.0-11.0 mg), Dipyridamole 3.6-4.4 mg. Excipients: Glycerin (for injection), Polysorbate 80 (II), Vitamin C. | Y—H52020031, approved by National Medical Products Administration | N |
| Zhang et al. (2013) | Ginkgo Leaf Extract and Dipyridamole Injection | Hubei Minkang Pharmaceutical Co., Ltd., China | Ginkgo biloba L | Dry leaves | China. Autumn | 5 mL per vial | Main components: Ginkgo biloba extract 20.5 mg (containing Ginkgo flavonoids 4.5-5.5 mg), Dipyridamole 1.8-2.2 mg.Excipients: polysorbate 80, hydrochloric acid, sodium hydroxide, water for injection. | Y—H42022870, approved by National Medical Products Administration | N |
| Tang et al. (2013) | Shuxuening Injection | [Commercial Supplier] | Ginkgo biloba L | Dry leaves | China. Autumn | [National Standard] | [National Standard] | Y—Approved by National Medical Products Administration | N |
| Mao et al. (2013) | Shuxuening Injection | HeiLongJiang ZBD Pharmaceutical Co., Ltd., China | Ginkgo biloba L | Dry leaves | China. Autumn | 5 mL per vial | Main components: Ginkgo biloba extract 17.5 mg (containing total flavonol glycosides 4.2 mg, ginkgolides 0.7 mg). Excipients: Ethanol, Sorbitol. | Y—Z23022003, approved by National Medical Products Administration | N |
| Zhang et al. (2014) | Ginkgo Leaf Extract and Dipyridamole Injection | [Commercial Supplier] | Ginkgo biloba L | Dry leaves | China. Autumn | [National Standard] | [National Standard] | Y—Approved by National Medical Products Administration | N |
| Li (2014) | Ginkgo Leaf Extract and Dipyridamole Injection | [Commercial Supplier] | Ginkgo biloba L | Dry leaves | China. Autumn | [National Standard] | [National Standard] | Y—Approved by National Medical Products Administration | N |
| Jiang et al. (2014) | Ginkgo Leaf Extract and Dipyridamole Injection | [Commercial Supplier] | Ginkgo biloba L | Dry leaves | China. Autumn | [National Standard] | [National Standard] | Y—Approved by National Medical Products Administration | N |
| Guo and Xu (2015) | Ginkgo Leaf Extract and Dipyridamole Injection | Guizhou Yibai Pharmaceutical Co., Ltd., China | Ginkgo biloba L | Dry leaves | China. Autumn | 5 mL per vial | Main components: Ginkgo biloba extract 20.5mg (containing Ginkgo flavonoids 4.5-5.5 mg), Dipyridamole 1.8-2.2 mg. Excipients: Glycerin (for injection), Polysorbate 80 (II), Vitamin C. | Y—H52020032, approved by National Medical Products Administration | N |
| Zhang (2015) | Ginkgo Leaf Extract and Dipyridamole Injection | Guizhou Yibai Pharmaceutical Co., Ltd., China | Ginkgo biloba L | Dry leaves | China. Autumn | 5 mL per vial | Main components: Ginkgo biloba extract 20.5mg (containing Ginkgo flavonoids 4.5-5.5 mg), Dipyridamole 1.8-2.2 mg. Excipients: Glycerin (for injection), Polysorbate 80 (II), Vitamin C. | Y—H52020032, approved by National Medical Products Administration | N |
| Hu et al. (2016) | Ginkgo Leaf Extract and Dipyridamole Injection | Guizhou Yibai Pharmaceutical Co., Ltd., China | Ginkgo biloba L | Dry leaves | China. Autumn | 10 mL per vial | Main components: Ginkgo biloba extract 41.0mg (containing Ginkgo flavonoids 9.0-11.0 mg), Dipyridamole 3.6-4.4 mg. Excipients: Glycerin (for injection), Polysorbate 80 (II), Vitamin C. | Y—H52020031, approved by National Medical Products Administration | N |
| Huang et al. (2017) | Ginkgo Leaf Extract and Dipyridamole Injection | Guizhou Yibai Pharmaceutical Co., Ltd., China | Ginkgo biloba L | Dry leaves | China. Autumn | 5 mL per vial | Main components: Ginkgo biloba extract 20.5mg (containing Ginkgo flavonoids 4.5-5.5 mg), Dipyridamole 1.8-2.2 mg. Excipients: Glycerin (for injection), Polysorbate 80 (II), Vitamin C. | Y—H52020032, approved by National Medical Products Administration | N |
| Wu et al. (2017) | Shuxuening Injection | HeiLongJiang ZBD Pharmaceutical Co., Ltd., China | Ginkgo biloba L | Dry leaves | China. Autumn | 2 ml per vial | Main components: Ginkgo biloba extract 7.0 mg (containing total flavonol glycosides 1.68 mg, ginkgolides 0.28 mg). Excipients: Ethanol, Sorbitol. | Y—Z23022004, approved by National Medical Products Administration | N |
| Shen (2017) | Ginkgo Leaf Extract and Dipyridamole Injection | Guizhou Yibai Pharmaceutical Co., Ltd., China | Ginkgo biloba L | Dry leaves | China. Autumn | 5 mL per vial | Main components: Ginkgo biloba extract 20.5mg (containing Ginkgo flavonoids 4.5-5.5 mg), Dipyridamole 1.8-2.2 mg. Excipients: Glycerin (for injection), Polysorbate 80 (II), Vitamin C. | Y—H52020032, approved by National Medical Products Administration | N |
| Cheng et al. (2018) | Ginkgo Leaf Extract and Dipyridamole Injection | Guizhou Yibai Pharmaceutical Co., Ltd., China | Ginkgo biloba L | Dry leaves | China. Autumn | 5 mL per vial | Main components: Ginkgo biloba extract 20.5mg (containing Ginkgo flavonoids 4.5-5.5 mg), Dipyridamole 1.8-2.2 mg. Excipients: Glycerin (for injection), Polysorbate 80 (II), Vitamin C. | Y—H52020032, approved by National Medical Products Administration | N |
| Hu (2019) | Ginkgo Biloba Softgel Capsules | [Commercial Supplier] | Ginkgo biloba L | Dry leaves | China. Autumn | [National Standard] | [National Standard] | Y—Approved by National Medical Products Administration | N |
| Zhu et al. (2020) | Ginkgo Leaf Extract and Dipyridamole Injection | Shanxi PUDE Pharmaceutical Co., Ltd., China | Ginkgo biloba L | Dry leaves | China. Autumn | 5 mL per vial | Main components: Ginkgo biloba extract 20.5mg (containing Ginkgo flavonoids 4.5-5.5 mg), Dipyridamole 1.8-2.2 mg.Excipients: Vitamin C, Propylene glycol, Polysorbate 80. | Y—H14023515, approved by National Medical Products Administration | N |
| Xing (2022) | Ginkgo Leaf Extract and Dipyridamole Injection | Tong Hua Gu Hong Pharmaceutical Co., Ltd., China | Ginkgo biloba L | Dry leaves | China. Autumn | 5 mL per vial | Main components: Ginkgo biloba extract 20.5mg (containing Ginkgo flavonoids 4.5-5.5 mg), Dipyridamole 1.8-2.2 mg. Excipients: propylene glycol, sodium chloride, polysorbate 80, disodium edetate, water for injection. | Y—H22026140, approved by National Medical Products Administration | N |

**Note:** The GBE formulations used in the 41 RCTs were all registered and approved by the National Medical Products Administration of China and manufactured by reputable, publicly listed pharmaceutical companies within the country. The quality control standards for all GBE formulations strictly adhered to the requirements of the Chinese Pharmacopoeia, specifying not less than 24% flavonoid glycosides and 6% terpene lactones (2.8-3.4% ginkgolides A, B and C, and 2.6-3.2% bilobalide). Due to the confidentiality surrounding drug manufacturing, access to detailed technical documentation from the manufacturers and specific information on the extraction and production processes is restricted.

**Reference:** Heinrich, M., Jalil, B., Abdel-Tawab, M., Echeverria, J., Kulić, Ž., McGaw, L.J., et al. (2022). Best Practice in the chemical characterisation of extracts used in pharmacological and toxicological research-The ConPhyMP-Guidelines. Front Pharmacol. 13, 953205. doi: 10.3389/fphar.2022.953205. For more information, visit: https://doi.org/10.3389/fphar.2022.953205

# Supplementary Material S5. Meta-regression of UAER, Scr, BUN, 24hUTP, FBG, TC and TG

4.1 Summary of meta-regression results.

| **Variable** | **Number of studies** | **Coefficient** | **95% CI** | **P-value** | **Tau^2^** | **AdjR^2^** |
| --- | --- | --- | --- | --- | --- | --- |
| UAER | 30 |  |  |  | 137.600 |  |
| Average age | 27 | -0.2546 | [-1.1323, 0.6232] | 0.556 | 150.600 | -2.87% |
| GBE dosage form | 30 | -2.6978 | [-24.7790, 19.3834] | 0.804 | 142.900 | -5.21% |
| Control preparation | 30 | 9.5467 | [-1.4452, 20.5386] | 0.086 | 124.700 | 8.20% |
| Sample size | 30 | -0.0112 | [-0.1297, 0.1072] | 0.847 | 142.300 | -4.70% |
| Scr | 37 |  |  |  | 73.980 |  |
| Average age | 31 | -0.3601 | [-1.0839, 0.3627] | 0.316 | 122.500 | -1.16% |
| GBE dosage form | 37 | -1.9123 | [-19.2397, 15.4151] | 0.824 | 106.300 | -3.01% |
| Control preparation | 37 | 3.1997 | [-4.6664, 11.0658] | 0.415 | 105.000 | -1.73% |
| Sample size | 37 | -0.0231 | [-0.1179, 0.0718] | 0.624 | 106.600 | -3.29% |
| BUN | 30 |  |  |  | 0.410 |  |
| Average age | 26 | 0.0070 | [-0.0477, 0.0618] | 0.793 | 0.532 | -5.70% |
| GBE dosage form | 30 | 0.4157 | [-0.9663, 1.7978] | 0.543 | 0.697 | -4.03% |
| Control preparation | 30 | 0.0571 | [-0.6708, 0.7850] | 0.874 | 0.702 | -4.82% |
| Sample size | 30 | -0.0024 | [-0.0109, 0.0062] | 0.576 | 0.694 | -3.59% |
| 24hUTP | 12 |  |  |  | 0.010 |  |
| Average age | 9 | -0.0195 | [-0.0752, 0.0363] | 0.436 | 0.046 | -4.91% |
| GBE dosage form | 12 | 0.0538 | [-0.5957, 0.7033] | 0.857 | 0.070 | -16.23% |
| Control preparation | 12 | -0.0543 | [-0.4315, 0.3228] | 0.755 | 0.067 | -11.15% |
| Sample size | 12 | 0.0002 | [-0.0032, 0.0037] | 0.878 | 0.070 | -15.77% |
| FBG | 23 |  |  |  | 0.240 |  |
| Average age | 19 | -0.0320 | [-0.0804, 0.0163] | 0.180 | 0.306 | 8.40% |
| GBE dosage form | 23 | 0.3749 | [-0.9519, 1.7017] | 0.563 | 0.318 | -3.62% |
| Control preparation | 23 | -0.0180 | [-0.7288, 0.6928] | 0.958 | 0.326 | -6.17% |
| Sample size | 23 | -0.0156 | [-0.0238, -0.0074] | 0.001 | 0.142 | 53.77% |
| TC | 15 |  |  |  | 0.340 |  |
| Average age | 13 | 0.0175 | [-0.0643, 0.0993] | 0.647 | 0.438 | -8.02% |
| GBE dosage form | 15 | -0.9145 | [-2.3604, 0.5313] | 0.195 | 0.347 | 5.63% |
| Control preparation | 15 | -0.1270 | [-0.9402, 0.6862] | 0.741 | 0.399 | -8.43% |
| Sample size | 15 | -0.0045 | [-0.0109, 0.0018] | 0.147 | 0.329 | 10.67% |
| TG | 15 |  |  |  | 0.070 |  |
| Average age | 13 | -0.0029 | [-0.0278, 0.0220] | 0.801 | 0.057 | -13.59% |
| GBE dosage form | 15 | 0.0264 | [-0.5151, 0.5680] | 0.918 | 0.050 | -11.70% |
| Control preparation | 15 | 0.4768 | [0.2343, 0.7193] | 0.001 | 0.009 | 78.91% |
| Sample size | 15 | -0.0025 | [-0.0060, 0.0010] | 0.153 | 0.036 | 18.56% |

4.2 Meta-regression analysis of UAER.


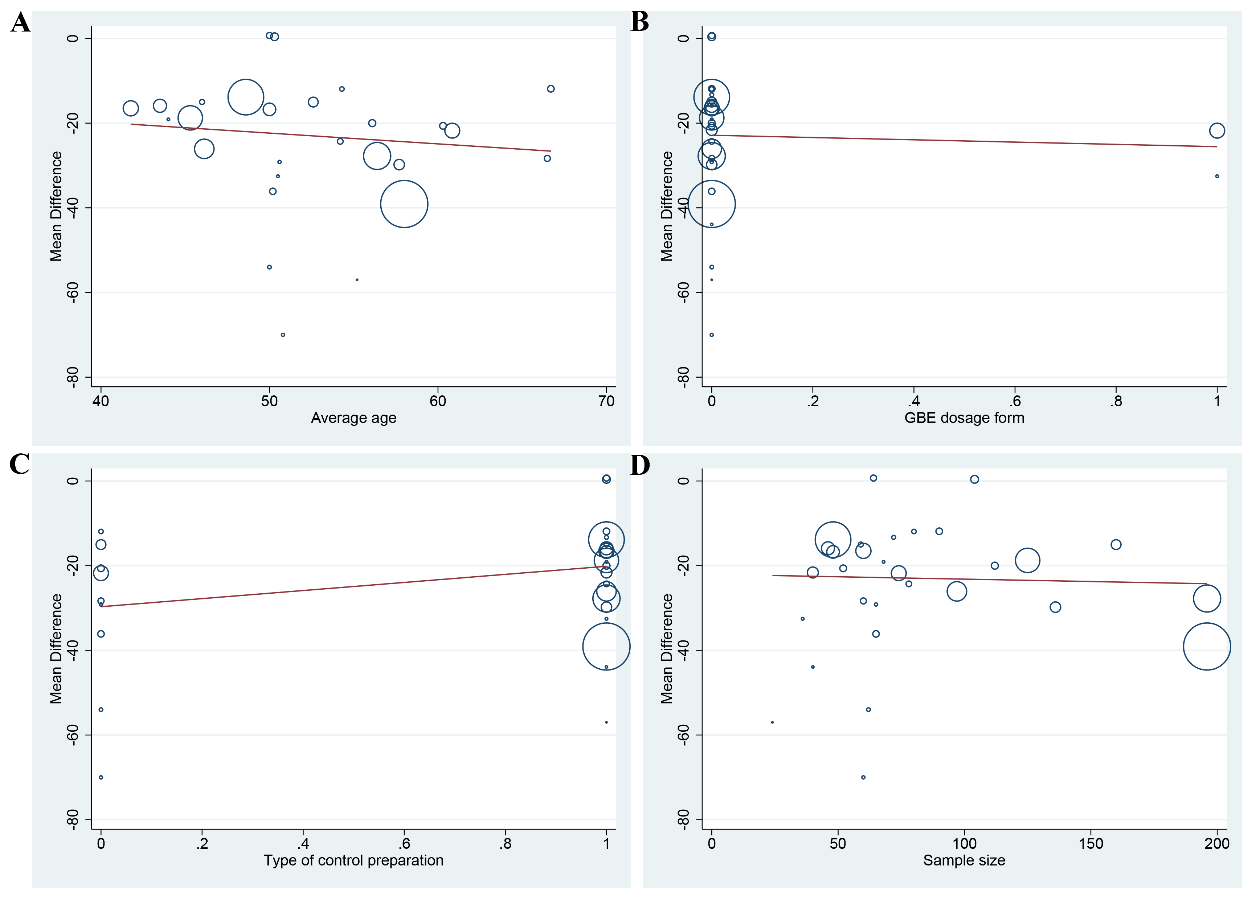


Meta-regression analysis of UAER on (a) Average age, (b) GBE dosage form, (c) Control preparation and (d) Sample size.

4.3 Meta-regression analysis of Scr.


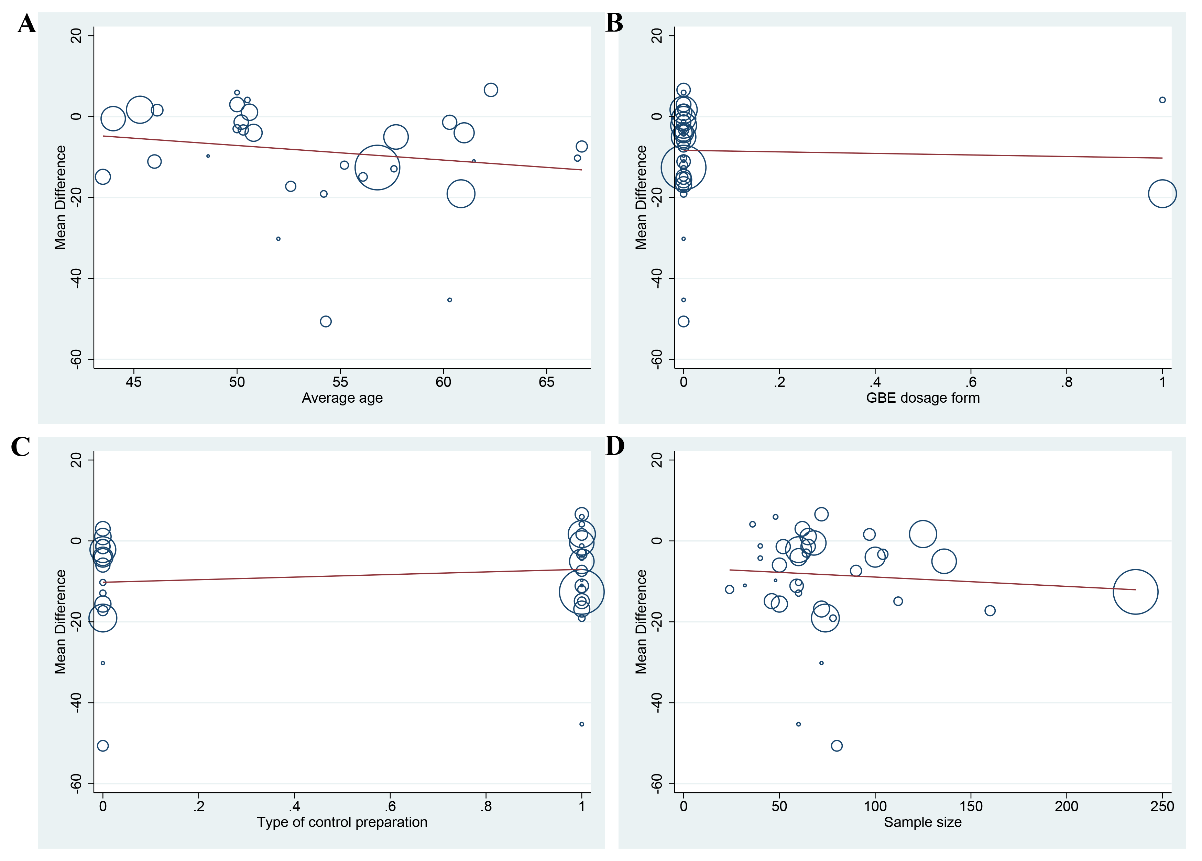


Meta-regression analysis of Scr on (a) Average age, (b) GBE dosage form, (c) Control preparation and (d) Sample size.

4.4 Meta-regression analysis of BUN.


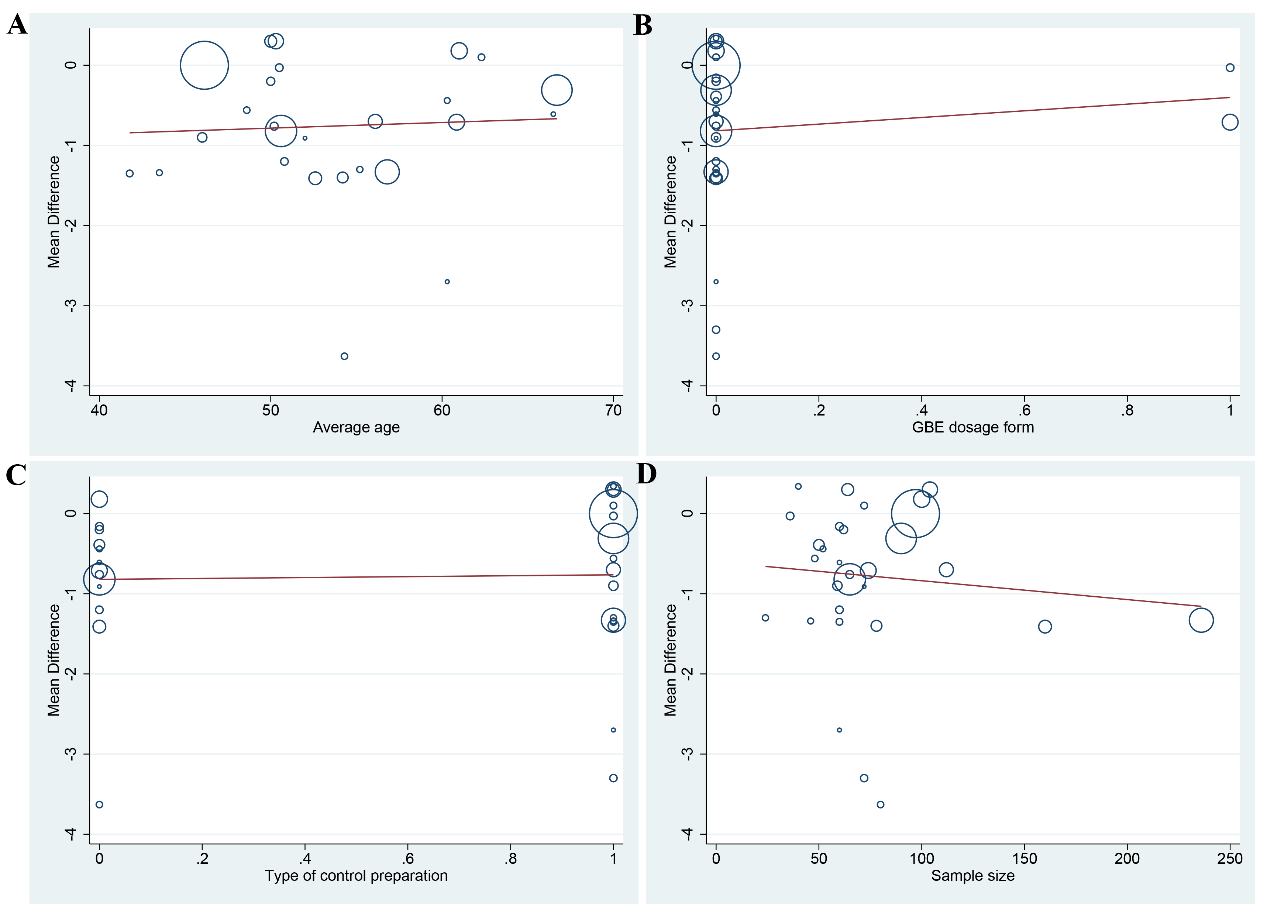


Meta-regression analysis of BUN on (a) Average age, (b) GBE dosage form, (c) Control preparation and (d) Sample size.

4.5 Meta-regression analysis of 24hUTP.


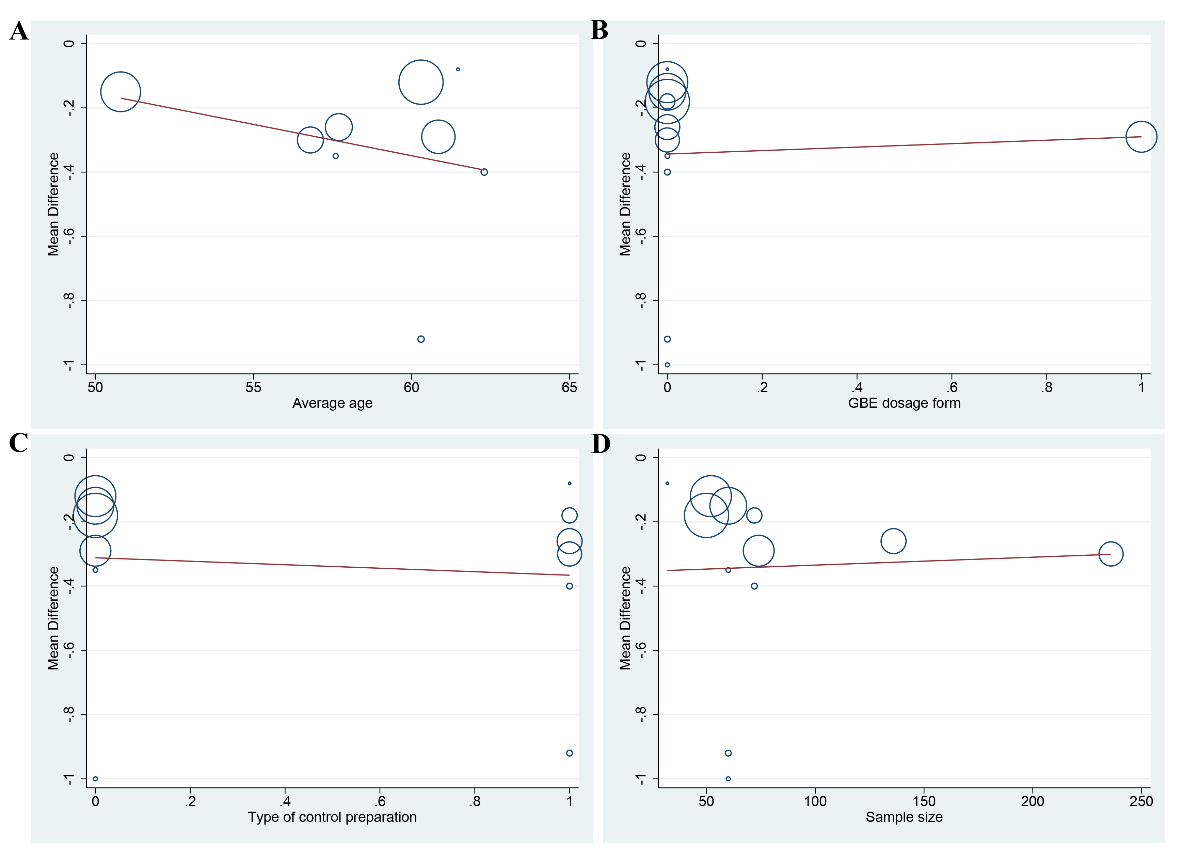


Meta-regression analysis of 24hUTP on (a) Average age, (b) GBE dosage form, (c) Control preparation and (d) Sample size.

4.6 Meta-regression analysis of FBG.


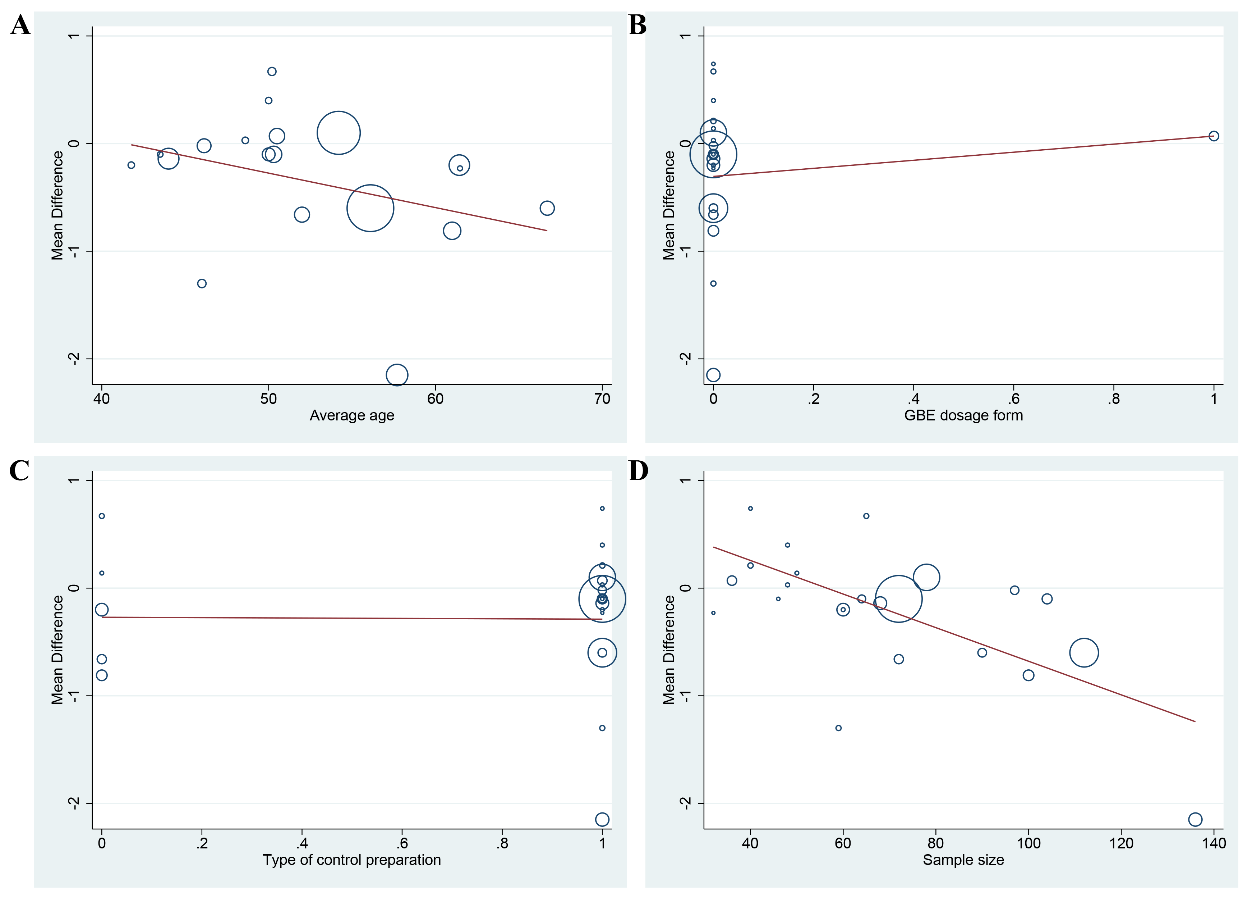


Meta-regression analysis of FBG on (a) Average age, (b) GBE dosage form, (c) Control preparation and (d) Sample size.

4.7 Meta-regression analysis of TC.


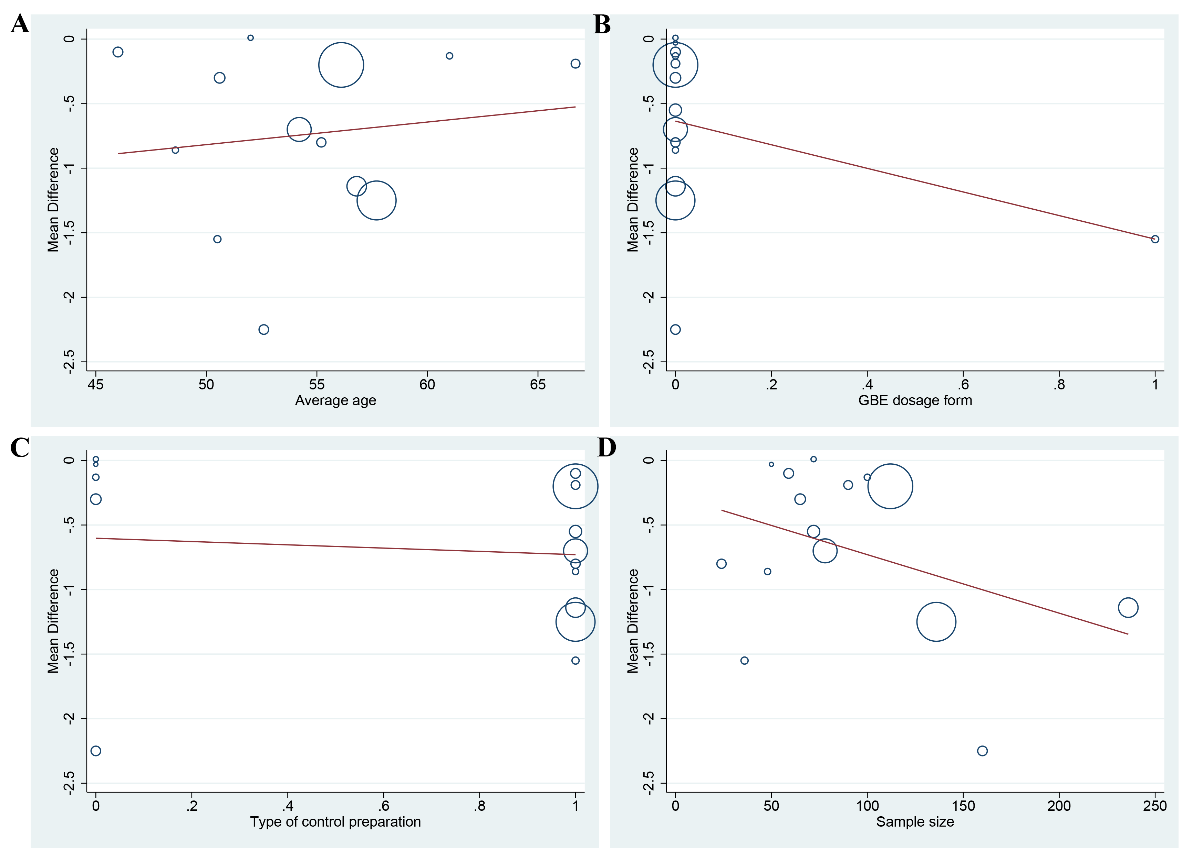


Meta-regression analysis of TC on (a) Average age, (b) GBE dosage form, (c) Control preparation and (d) Sample size.

4.8 Meta-regression analysis of TG.


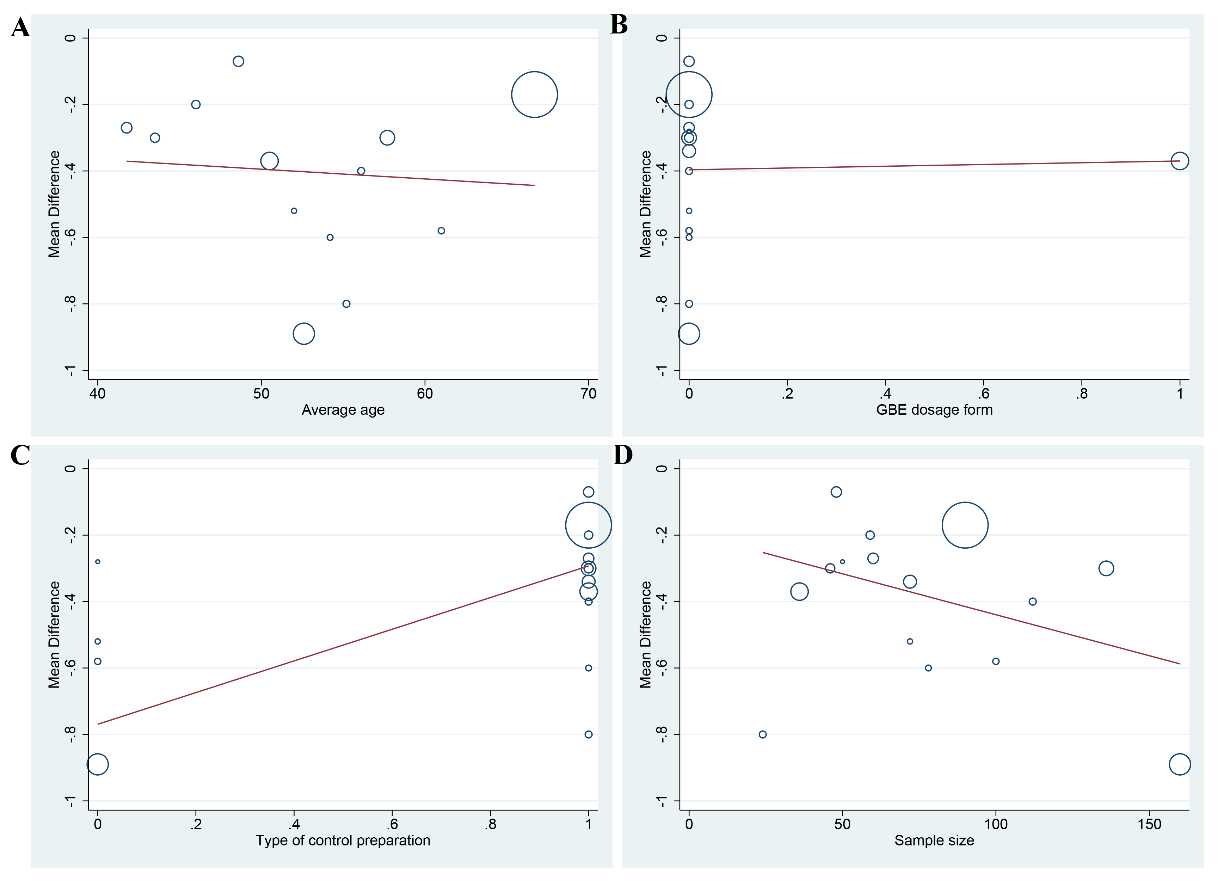


Meta-regression analysis of TG on (a) Average age, (b) GBE dosage form, (c) Control preparation and (d) Sample size.

# Supplementary Material S6. Subgroup analysis of UAER, Scr, BUN, 24hUTP, FBG, TC, TG, SBP and DBP.

5.1 Summary of subgroup analysis results.

| **Outcomes** | **Subgroup** | **Number of**  **studies** | **MD (95% CI)** | **P-value for overall effect** | **I^2^(%)** | **P-value for subgroup difference** |
| --- | --- | --- | --- | --- | --- | --- |
| UAER | Total | 30 | -22.99 [-27.66, -18.31] | ＜0.001 | 96 | - |
|  | Average age ≤ 60 years old | 23 | -23.44 [-28.97, -17.90] | ＜0.001 | 97 | 0.49 |
|  | Average age＞60 years old | 4 | -20.72 [-26.11, -15.33] | ＜0.001 | 48 |  |
|  | Injection | 28 | -22.86 [-27.78, -17.94] | ＜0.001 | 97 | 0.82 |
|  | Capsule | 2 | -22.13 [-26.18, -18.08] | ＜0.001 | 0 |  |
|  | ACEI | 9 | -29.65 [-37.89, -21.40] | ＜0.001 | 85 | 0.06 |
|  | ARB | 21 | -20.21 [-25.87, -14.55] | ＜0.001 | 97 |  |
|  | Sample size＜80 cases | 20 | -22.95 [-27.09, -18.81] | ＜0.001 | 83 | 0.58 |
|  | Sample size≥80 cases | 10 | -20.58 [-27.76, -13.41] | ＜0.001 | 98 |  |
| Scr | Total | 37 | -8.30 [-11.55, -5.05] | ＜0.001 | 89 | - |
|  | Average age ≤ 60 years old | 23 | -8.25 [-12.54, -3.97] | ＜0.001 | 90 | 0.92 |
|  | Average age＞60 years old | 8 | -8.76 [-17.36, -0.15] | 0.046# | 90 |  |
|  | Injection | 35 | -8.10 [-11.33, -4.87] | ＜0.001 | 88 | 0.94 |
|  | Capsule | 2 | -8.95 [-31.48, 13.58] | 0.44 | 86 |  |
|  | ACEI | 15 | -10.29 [-16.28, -4.31] | ＜0.001 | 93 | 0.34 |
|  | ARB | 22 | -6.81 [-10.59, -3.03] | ＜0.001 | 83 |  |
|  | Sample size＜80 cases | 27 | -7.15 [-10.87, -3.42] | ＜0.001 | 84 | 0.35 |
|  | Sample size≥80 cases | 10 | -10.79 [-17.51, -4.07] | 0.002 | 95 |  |
| BUN | Total | 30 | -0.77 [-1.04, -0.49] | ＜0.001 | 88 | - |
|  | Average age ≤ 60 years old | 19 | -0.85 [-1.21, -0.49] | ＜0.001 | 90 | 0.09 |
|  | Average age＞60 years old | 7 | -0.38 [-0.78, 0.02] | 0.06 | 65 |  |
|  | Injection | 28 | -0.80 [-1.09, -0.51] | ＜0.001 | 88 | 0.38 |
|  | Capsule | 2 | -0.48 [-1.11, 0.15] | 0.13 | 46 |  |
|  | ACEI | 13 | -0.80 [-1.20, -0.40] | ＜0.001 | 80 | 0.84 |
|  | ARB | 17 | -0.74 [-1.12, -0.36] | ＜0.001 | 90 |  |
|  | Sample size＜80 cases | 22 | -0.78 [-1.08, -0.48] | ＜0.001 | 70 | 0.89 |
|  | Sample size≥80 cases | 8 | -0.74 [-1.24, -0.23] | 0.004 | 95 |  |
| 24hUTP | Total | 12 | -0.28 [-0.35, -0.22] | ＜0.001 | 91 | - |
|  | Average age ≤ 60 years old | 4 | -0.24 [-0.33, -0.15] | ＜0.001 | 88 | 0.22 |
|  | Average age＞60 years old | 5 | -0.36 [-0.53, -0.19] | ＜0.001 | 95 |  |
|  | Injection | 11 | -0.28 [-0.35, -0.21] | ＜0.001 | 91 | 0.83 |
|  | Capsule | 1 | -0.29 [-0.33, -0.25] | ＜0.001 | - |  |
|  | ACEI | 6 | -0.23 [-0.30, -0.15] | ＜0.001 | 92 | 0.09 |
|  | ARB | 6 | -0.35 [-0.47, -0.23] | ＜0.001 | 86 |  |
|  | Sample size＜80 cases | 10 | -0.29 [-0.36, -0.21] | ＜0.001 | 92 | 0.89 |
|  | Sample size≥80 cases | 2 | -0.28 [-0.32, -0.24] | ＜0.001 | 1 |  |
| FBG | Total | 23 | -0.30 [-0.54, -0.05] | 0.02 | 87 | - |
|  | Average age ≤ 60 years old | 15 | -0.32 [-0.70, 0.06] | 0.10 | 90 | 0.49 |
|  | Average age＞60 years old | 4 | -0.50 [-0.83, -0.16] | 0.003 | 31 |  |
|  | Injection | 22 | -0.32 [-0.57, -0.06] | 0.02 | 87 | 0.19 |
|  | Capsule | 1 | 0.07 [-0.44, 0.58] | 0.79 | - |  |
|  | ACEI | 5 | -0.32 [-0.76, 0.12] | 0.15 | 62 | 0.92 |
|  | ARB | 18 | -0.30 [-0.59, -0.01] | 0.046# | 89 |  |
|  | Sample size＜80 cases | 17 | -0.08 [-0.21, 0.06] | 0.26 | 23 | 0.04 |
|  | Sample size≥80 cases | 6 | -0.73 [-1.33, -0.13] | 0.02 | 93 |  |
| TC | Total | 15 | -0.69 [-1.01, -0.38] | ＜0.001 | 96 | - |
|  | Average age ≤ 60 years old | 11 | -0.84 [-1.21, -0.46] | ＜0.001 | 97 | 0.01 |
|  | Average age＞60 years old | 2 | -0.17 [-0.52, 0.18] | 0.34 | 0 |  |
|  | Injection | 14 | -0.64 [-0.96, -0.31] | ＜0.001 | 96 | 0.004 |
|  | Capsule | 1 | -1.55 [-2.08, -1.02] | ＜0.001 | - |  |
|  | ACEI | 5 | -0.56 [-1.57, 0.44] | 0.27 | 94 | 0.76 |
|  | ARB | 10 | -0.73 [-1.09, -0.37] | ＜0.001 | 97 |  |
|  | Sample size＜80 cases | 9 | -0.57 [-0.83, -0.32] | ＜0.001 | 73 | 0.36 |
|  | Sample size≥80 cases | 6 | -0.87 [-1.45, -0.29] | 0.003 | 99 |  |
| TG | Total | 15 | -0.40 [-0.56, -0.23] | ＜0.001 | 85 | - |
|  | Average age ≤ 60 years old | 11 | -0.42 [-0.62, -0.23] | ＜0.001 | 81 | 0.59 |
|  | Average age＞60 years old | 2 | -0.31 [-0.68, 0.07] | 0.11 | 65 |  |
|  | Injection | 14 | -0.40 [-0.58, -0.22] | ＜0.001 | 86 | 0.81 |
|  | Capsule | 1 | -0.37 [-0.54, -0.20] | ＜0.001 | - |  |
|  | ACEI | 4 | -0.71 [-0.97, -0.44] | ＜0.001 | 38 | 0.004 |
|  | ARB | 11 | -0.29 [-0.39, -0.20] | ＜0.001 | 44 |  |
|  | Sample size＜80 cases | 10 | -0.33 [-0.44, -0.23] | ＜0.001 | 11 | 0.50 |
|  | Sample size≥80 cases | 5 | -0.46 [-0.83, -0.10] | 0.01 | 95 |  |
| SBP | Total | 10 | -5.99 [-12.76, 0.79] | 0.08 | 96 | - |
|  | ACEI | 1 | -29.70 [-34.51, -24.89] | ＜0.001 | - | ＜0.001 |
|  | ARB | 9 | -3.25 [-8.50, 2.00] | 0.23 | 93 |  |
|  | Sample size＜80 cases | 6 | -10.05 [-22.06, 1.95] | 0.10 | 97 | 0.13 |
|  | Sample size≥80 cases | 4 | -0.06 [-4.58, 4.46] | 0.98 | 86 |  |
| DBP | Total | 10 | -3.46 [-7.93, 1.00] | 0.13 | 97 | - |
|  | ACEI | 1 | -14.78 [-16.30, -13.26] | ＜0.001 | - | ＜0.001 |
|  | ARB | 9 | -2.05 [-4.51, 0.41] | 0.10 | 84 |  |
|  | Sample size＜80 cases | 6 | -3.74 [-10.32, 2.85] | 0.27 | 97 | 0.83 |
|  | Sample size≥80 cases | 4 | -2.88 [-7.44, 1.67] | 0.22 | 92 |  |

Note: #: The P-values in RevMan are displayed as 0.05, and Stata is used to calculate P-values with precision up to three decimal places.

5.2 Subgroup analysis of UAER.


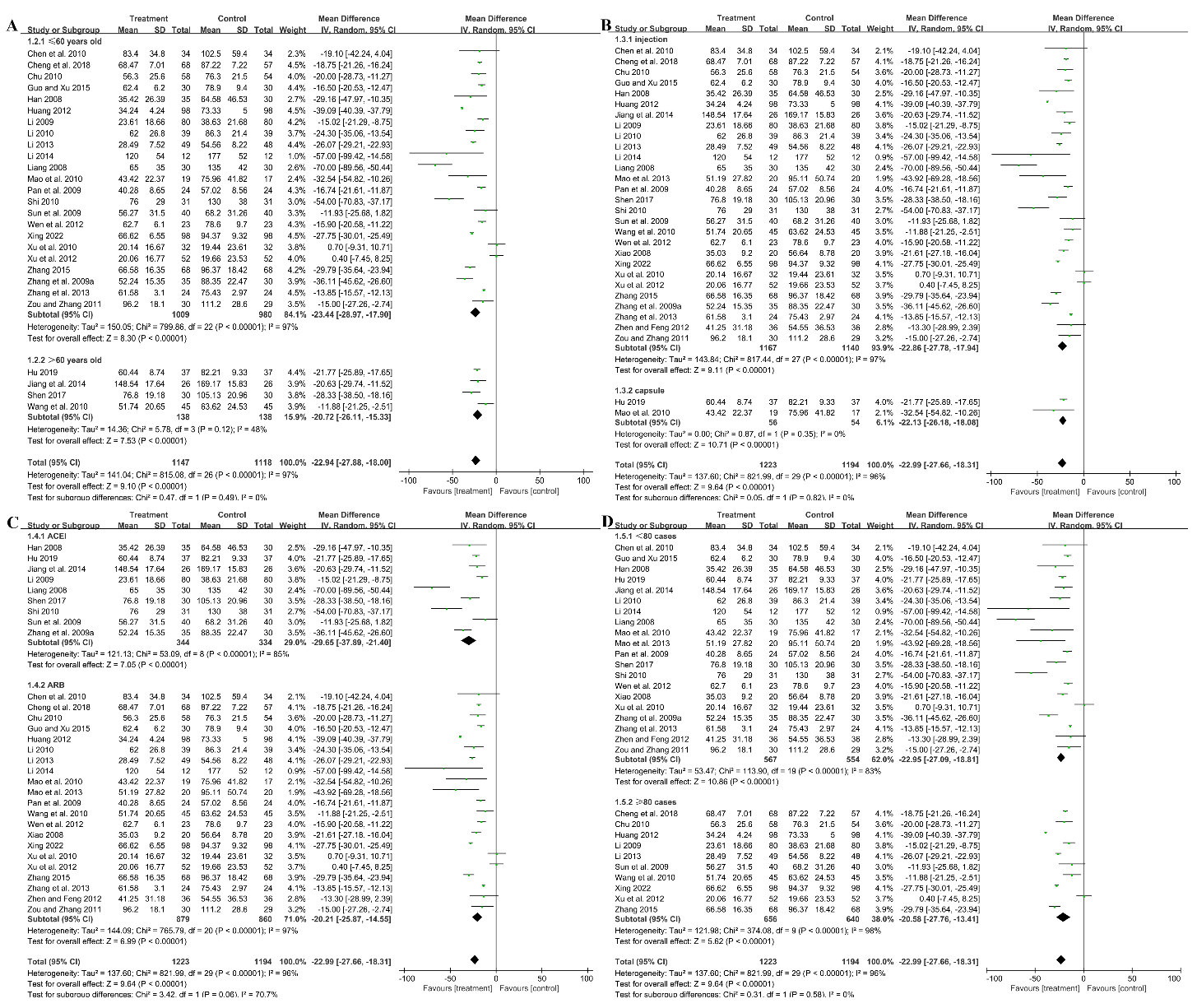


Subgroup analysis of UAER on (a) Average age, (b) GBE dosage form, (c) Control preparation and (d) Sample size.

5.3 Subgroup analysis of Scr.


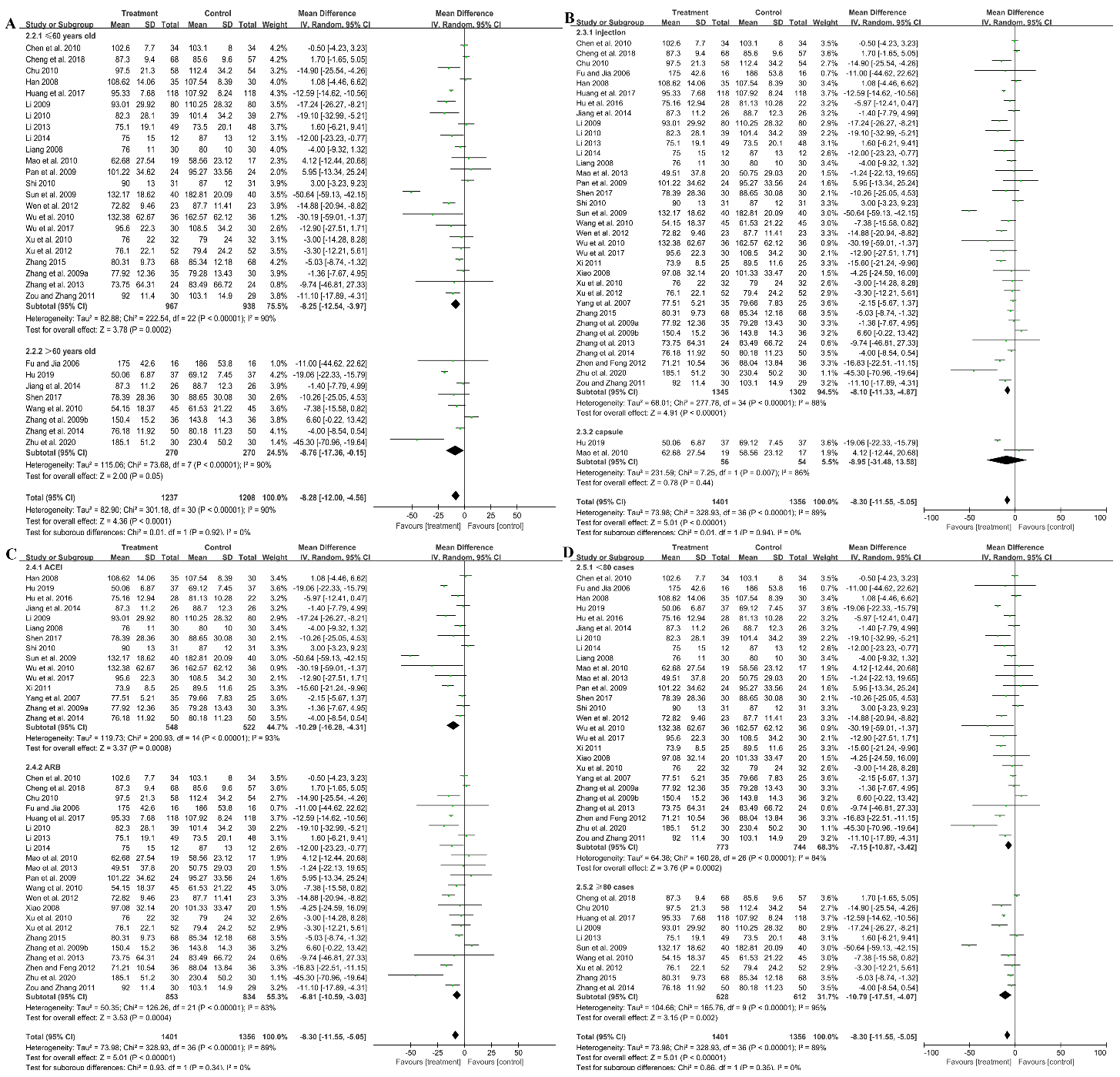


Subgroup analysis of Scr on (a) Average age, (b) GBE dosage form, (c) Control preparation and (d) Sample size.

5.4 Subgroup analysis of BUN.


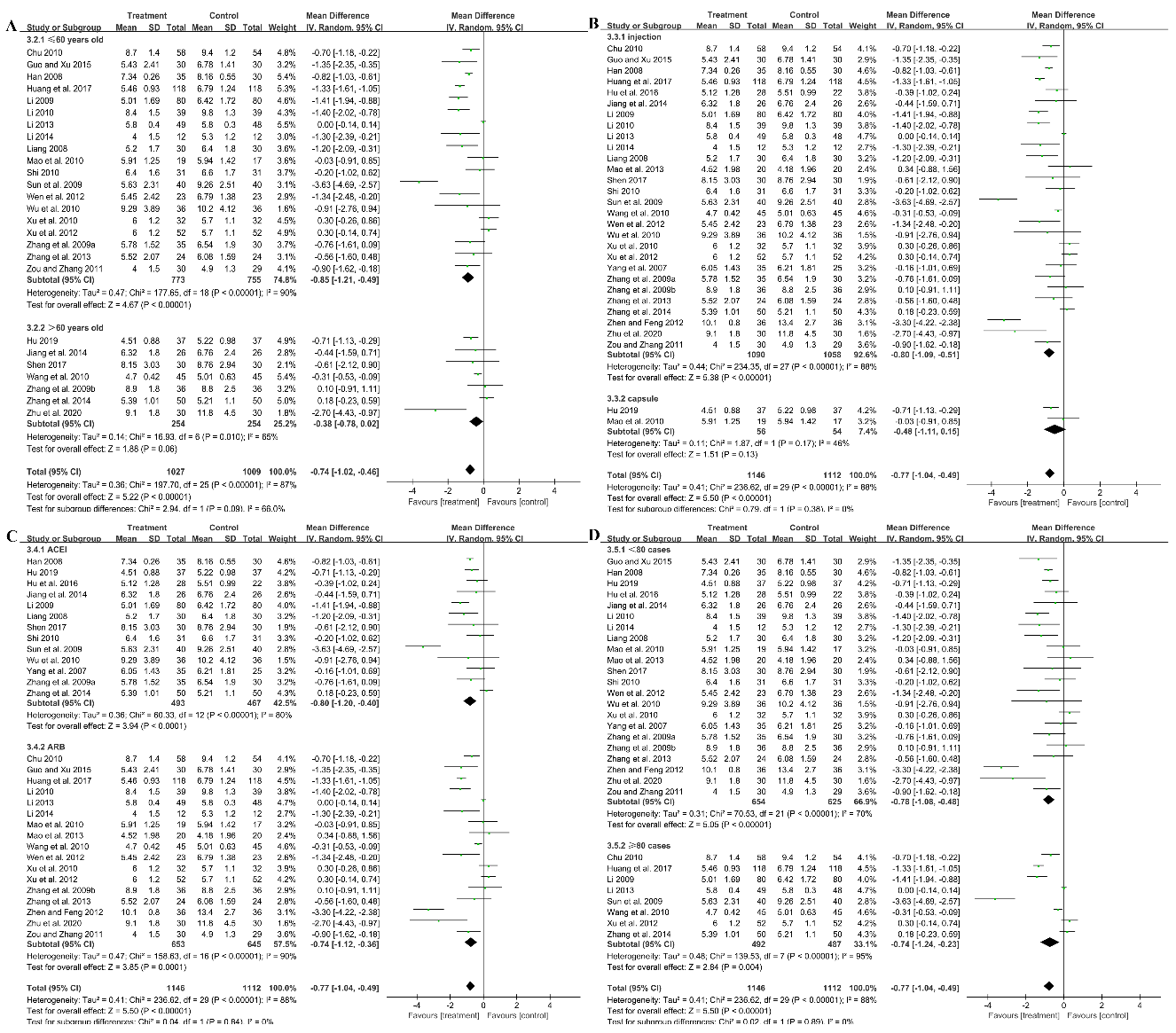


Subgroup analysis of BUN on (a) Average age, (b) GBE dosage form, (c) Control preparation and (d) Sample size.

5.5 Subgroup analysis of 24hUTP.


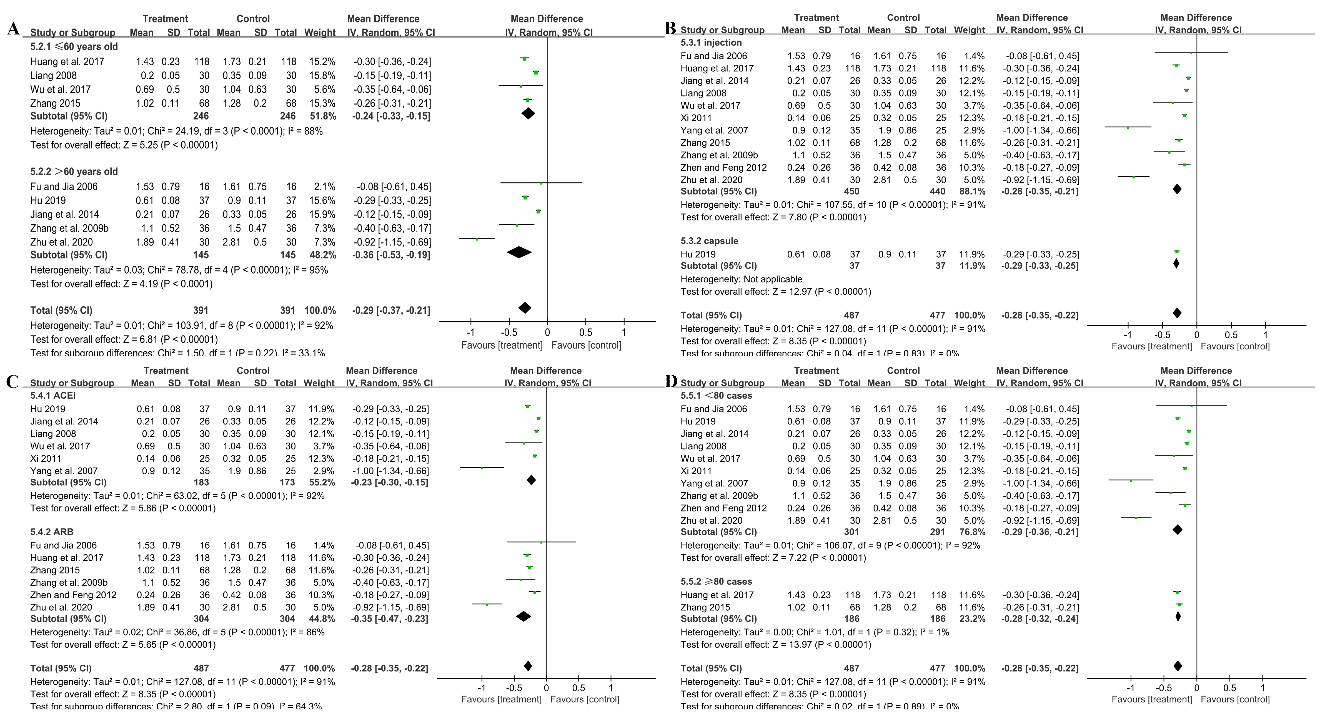


Subgroup analysis of 24hUTP on (a) Average age, (b) GBE dosage form, (c) Control preparation and (d) Sample size.

5.6 Subgroup analysis of FBG.


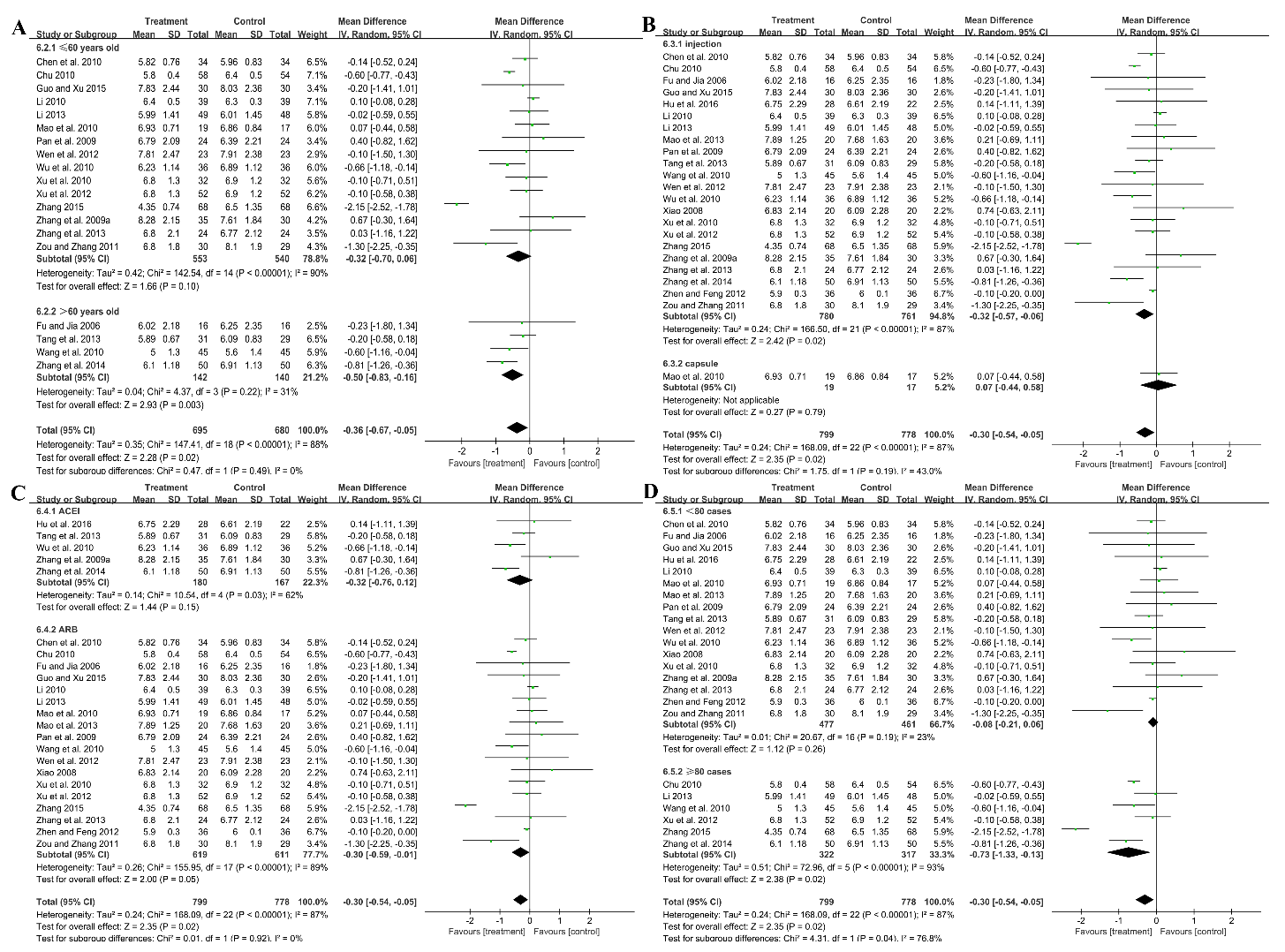


Subgroup analysis of FBG on (a) Average age, (b) GBE dosage form, (c) Control preparation and (d) Sample size.

5.7 Subgroup analysis of TC.


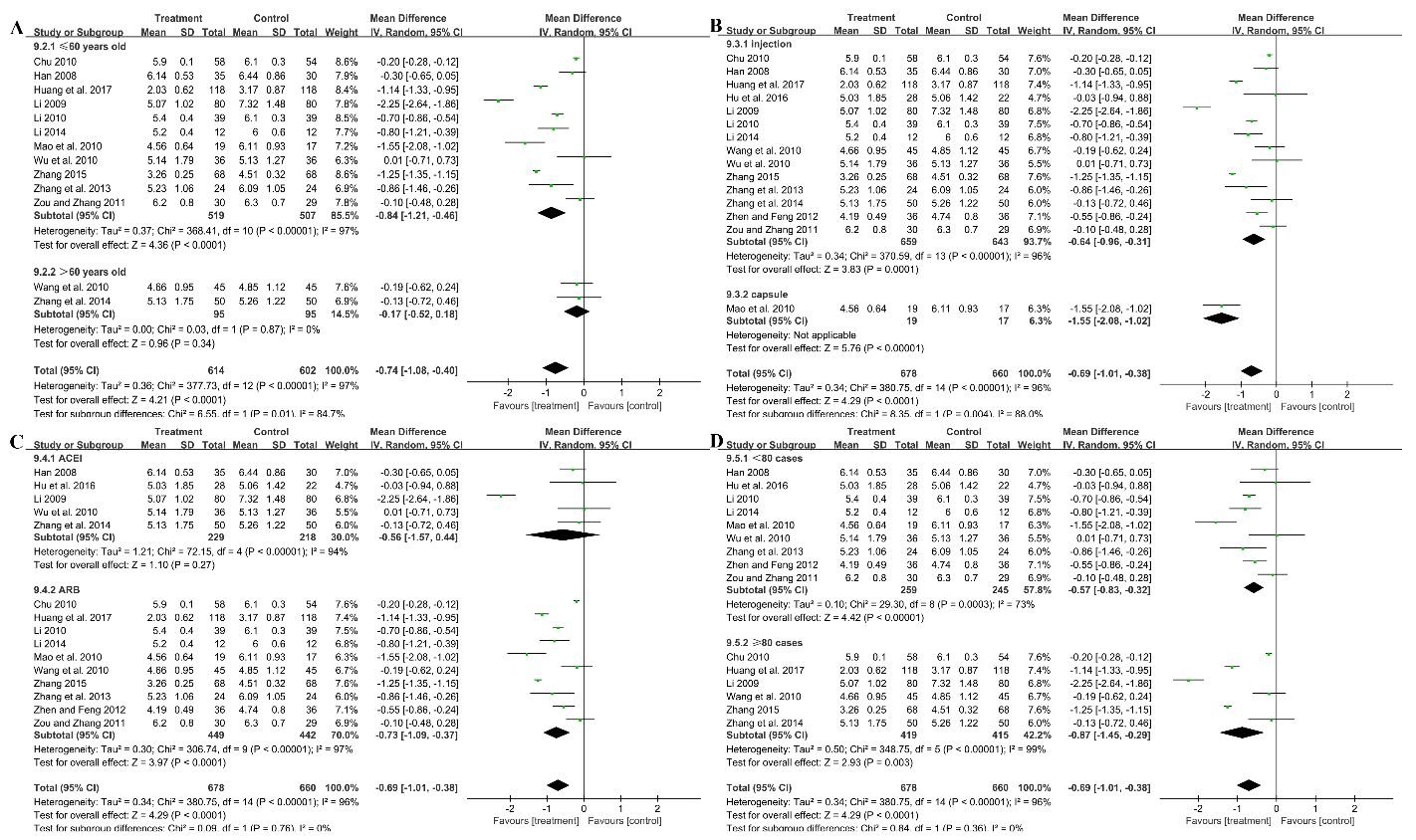


Subgroup analysis of TC on (a) Average age, (b) GBE dosage form, (c) Control preparation and (d) Sample size.

5.8 Subgroup analysis of TG.


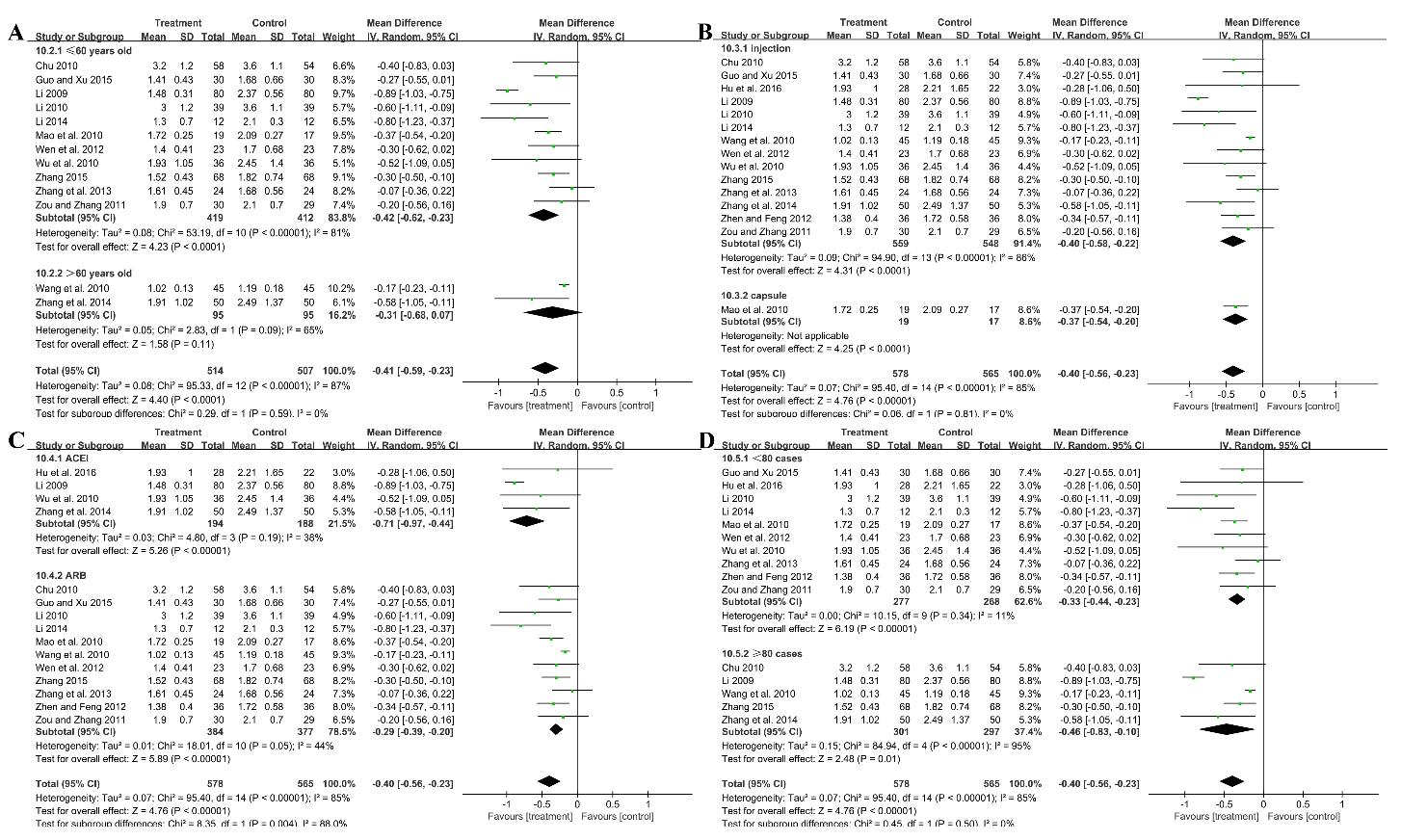


Subgroup analysis of TG on (a) Average age, (b) GBE dosage form, (c) Control preparation and (d) Sample size.

5.9 Subgroup analysis of SBP.


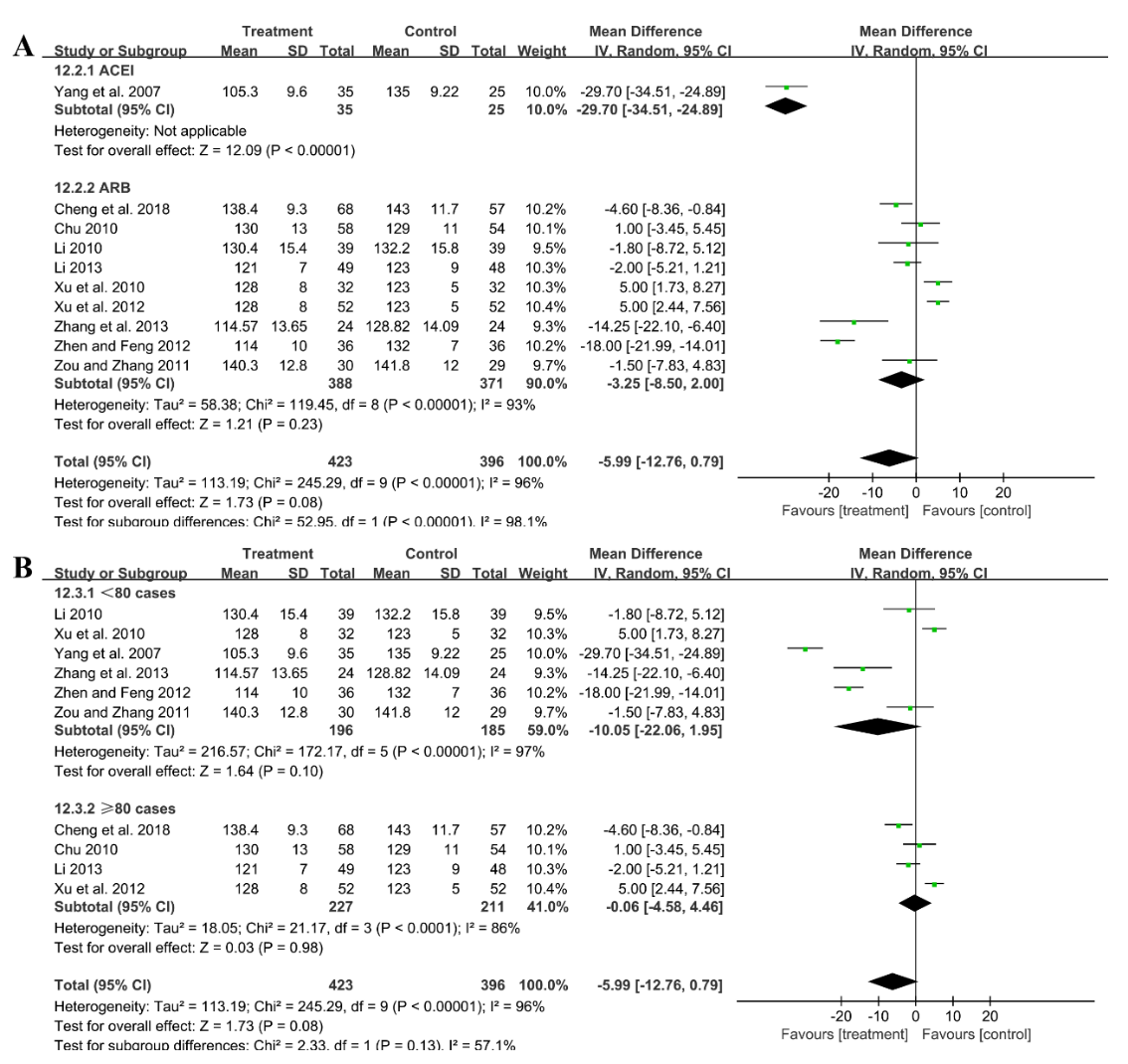


Subgroup analysis of SBP on (a) Control preparation and (b) Sample size.

5.10 Subgroup analysis of DBP.


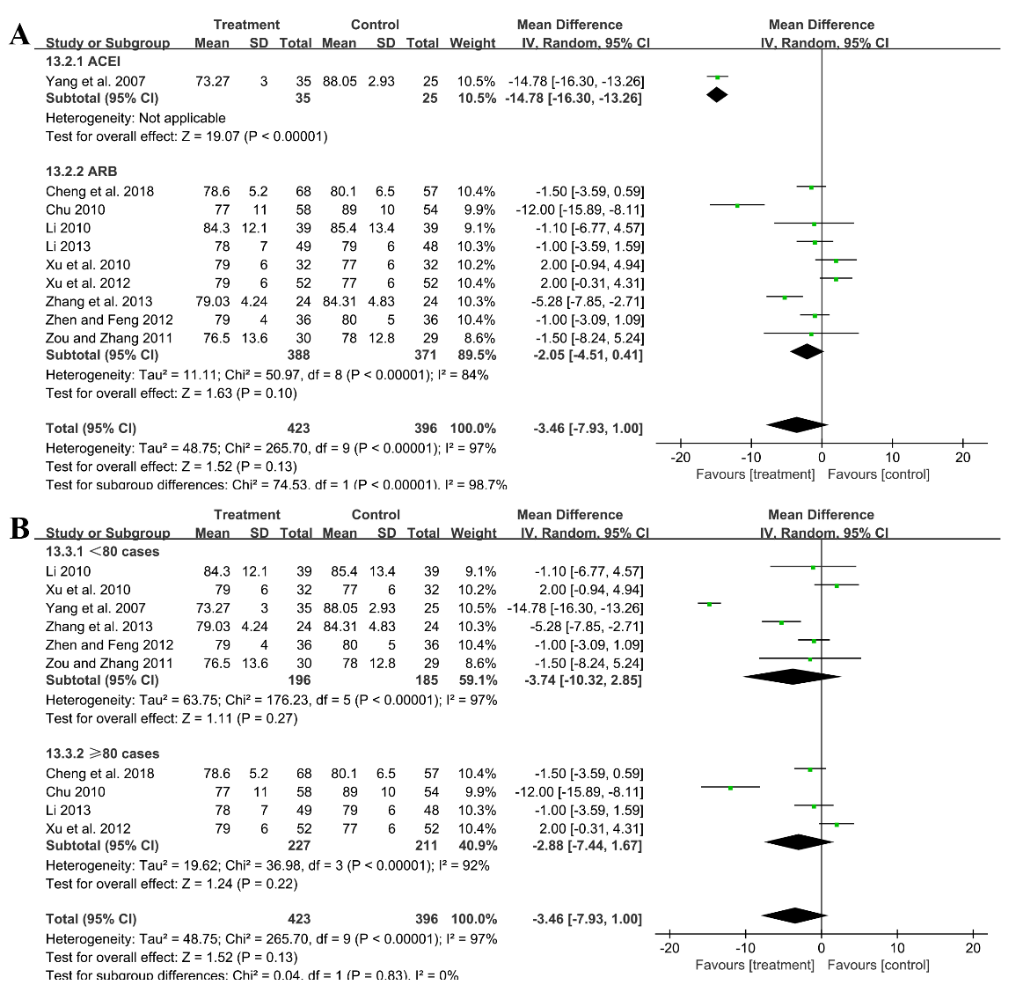


Subgroup analysis of DBP on (a) Control preparation and (b) Sample size

# Supplementary Material S7. Sensitivity analysis.

6.1 The results of sensitivity analysis of UAER.

6.2 The results of sensitivity analysis of Scr.

6.3 The results of sensitivity analysis of BUN.

6.4 The results of sensitivity analysis of 24hUTP.

6.5 The results of sensitivity analysis of FBG.

6.6 The results of sensitivity analysis of 2hPG.

6.7 The results of sensitivity analysis of HbA1c.

6.8 The results of sensitivity analysis of TC.

6.9 The results of sensitivity analysis of TG.

6.10 The results of sensitivity analysis of LDL-C.

6.11 The results of sensitivity analysis of SBP.

6.12 The results of sensitivity analysis of DBP.

6.13 The results of sensitivity analysis of SOD after changing the effect model.


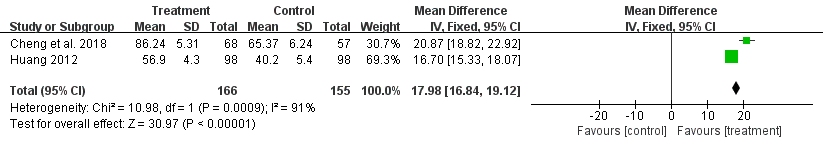


6.14 The results of sensitivity analysis of AOPP after changing the effect model.


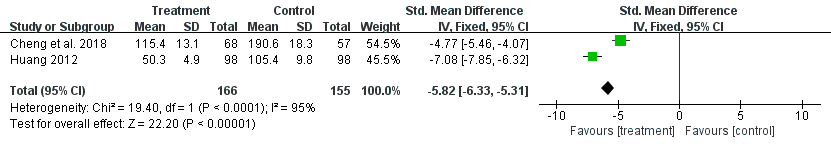


6.15 The results of sensitivity analysis of hs-CRP after changing the effect model.


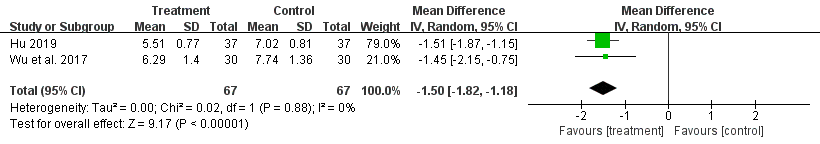


6.16 The results of sensitivity analysis of IL-6.

6.17 The results of sensitivity analysis of hematocrit.

6.18 The results of sensitivity analysis of fibrinogen.

6.19 The results of sensitivity analysis of fibrinogen after excluding Huang et al. 2017.


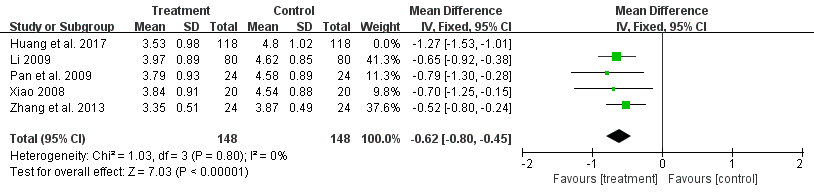


# Supplementary Material S8. Publication Bias

7.1 The results of publication bias of UAER.

7.2 The results of publication bias of Scr.

7.3 The results of publication bias of FBG.

7.4 The results of publication bias of TC.

7.5 The results of publication bias of TG.

7.6 The results of publication bias of SBP.

7.7 The results of publication bias of DBP.

7.8.1 The results of publication bias of BUN.

7.8.2The results of trim and fill analysis of BUN.

7.9.1 The results of publication bias of 24hUTP.

7.9.2The results of trim and fill analysis of 24hUTP.

# Supplementary Material S9. Assessment of evidence quality for each outcome.

| **Quality assessment** | | | | | | | **No of patients** | | **Effect** | | **Quality** | **Importance** |  |
| --- | --- | --- | --- | --- | --- | --- | --- | --- | --- | --- | --- | --- | --- |
|  |  |  |  |  |  |  |  |  |  |  |  |  |  |
| **No of studies** | **Design** | **Risk of bias** | **Inconsistency** | **Indirectness** | **Imprecision** | **Other considerations** | **Treatment** | **Control** | **Relative (95% CI)** | **Absolute** |  |  |  |
| **UAER (Better indicated by lower values)** | | | | | | | | | | | | |  |
| 30 | randomised trials | serious^1^ | serious^2^ | no serious indirectness | no serious imprecision | none | 1223 | 1194 | - | MD 22.99 lower (27.66 to 18.31 lower) | ⊕⊕OO LOW | CRITICAL |  |
| **SCr (Better indicated by lower values)** | | | | | | | | | | | | |  |
| 37 | randomised trials | serious^1^ | serious^2^ | no serious indirectness | no serious imprecision | none | 1401 | 1356 | - | MD 8.3 lower (11.55 to 5.05 lower) | ⊕⊕OO LOW | CRITICAL |  |
| **BUN (Better indicated by lower values)** | | | | | | | | | | | | |  |
| 30 | randomised trials | serious^1^ | serious^2^ | no serious indirectness | no serious imprecision | reporting bias^4^ | 1146 | 1112 | - | MD 0.77 lower (1.04 to 0.49 lower) | ⊕OOO VERY LOW | CRITICAL |  |
| **Cys-C (Better indicated by lower values)** | | | | | | | | | | | | |  |
| 1 | randomised trials | serious^1^ | no serious inconsistency | no serious indirectness | no serious imprecision | none | 30 | 30 | - | MD 0.3 lower (0.43 to 0.17 lower) | ⊕⊕⊕O MODERATE | CRITICAL |  |
| **24hUTP (Better indicated by lower values)** | | | | | | | | | | | | |  |
| 12 | randomised trials | serious^1^ | serious^2^ | no serious indirectness | no serious imprecision | reporting bias^4^ | 487 | 477 | - | MD 0.28 lower (0.35 to 0.22 lower) | ⊕OOO VERY LOW | CRITICAL |  |
| **FBG (Better indicated by lower values)** | | | | | | | | | | | | |  |
| 23 | randomised trials | serious^1^ | serious^2^ | no serious indirectness | no serious imprecision | none | 799 | 778 | - | MD 0.3 lower (0.54 to 0.05 lower) | ⊕⊕OO LOW | IMPORTANT |  |
| **2hPG (Better indicated by lower values)** | | | | | | | | | | | | |  |
| 4 | randomised trials | serious^1^ | serious^2^ | no serious indirectness | serious^3^ | none | 174 | 171 | - | MD 1.32 lower (3.43 lower to 0.8 higher) | ⊕OOO VERY LOW | IMPORTANT |  |
| **HbA1c (Better indicated by lower values)** | | | | | | | | | | | | |  |
| 5 | randomised trials | serious^1^ | serious^2^ | no serious indirectness | serious^3^ | none | 165 | 163 | - | MD 0.02 lower (1.07 lower to 1.03 higher) | ⊕OOO VERY LOW | IMPORTANT |  |
| **TC (Better indicated by lower values)** | | | | | | | | | | | | |  |
| 15 | randomised trials | serious^1^ | serious^2^ | no serious indirectness | no serious imprecision | none | 678 | 660 | - | MD 0.69 lower (1.01 to 0.38 lower) | ⊕⊕OO LOW | IMPORTANT |  |
| **TG (Better indicated by lower values)** | | | | | | | | | | | | |  |
| 15 | randomised trials | serious^1^ | serious^2^ | no serious indirectness | no serious imprecision | none | 578 | 565 | - | MD 0.4 lower (0.56 to 0.23 lower) | ⊕⊕OO LOW | IMPORTANT |  |
| **LDL-C (Better indicated by lower values)** | | | | | | | | | | | | |  |
| 3 | randomised trials | serious^1^ | serious^2^ | no serious indirectness | no serious imprecision | none | 126 | 120 | - | MD 0.97 lower (1.28 to 0.65 lower) | ⊕⊕OO LOW | IMPORTANT |  |
| **SBP (Better indicated by lower values)** | | | | | | | | | | | | |  |
| 10 | randomised trials | serious^1^ | serious^2^ | no serious indirectness | serious^3^ | none | 423 | 396 | - | MD 5.99 lower (12.76 lower to 0.79 higher) | ⊕OOO VERY LOW | IMPORTANT |  |
| **DBP (Better indicated by lower values)** | | | | | | | | | | | | |  |
| 10 | randomised trials | serious^1^ | serious^2^ | no serious indirectness | serious^3^ | none | 423 | 396 | - | MD 3.46 lower (7.93 lower to 1 higher) | ⊕OOO VERY LOW | IMPORTANT |  |
| **MDA (Better indicated by lower values)** | | | | | | | | | | | | |  |
| 2 | randomised trials | serious^1^ | serious^2^ | no serious indirectness | serious^3^ | none | 166 | 155 | - | SMD 6.94 lower (14.43 lower to 0.54 higher) | ⊕OOO VERY LOW | IMPORTANT |  |
| **SOD (Better indicated by higher values)** | | | | | | | | | | | | |  |
| 2 | randomised trials | serious^1^ | serious^2^ | no serious indirectness | serious^3^ | none | 166 | 155 | - | MD 18.71 higher (14.63 to 22.8 higher) | ⊕OOO VERY LOW | IMPORTANT |  |
| **AOPP (Better indicated by lower values)** | | | | | | | | | | | | |  |
| 2 | randomised trials | serious^1^ | serious^2^ | no serious indirectness | serious^3^ | none | 166 | 155 | - | SMD 5.92 lower (8.19 to 3.65 lower) | ⊕OOO VERY LOW | IMPORTANT |  |
| **hs-CRP (Better indicated by lower values)** | | | | | | | | | | | | |  |
| 2 | randomised trials | serious^1^ | no serious inconsistency | no serious indirectness | no serious imprecision | none | 67 | 67 | - | MD 1.5 lower (1.82 to 1.18 lower) | ⊕⊕⊕O MODERATE | IMPORTANT |  |
| **IL-6 (Better indicated by lower values)** | | | | | | | | | | | | |  |
| 3 | randomised trials | serious^1^ | serious^2^ | no serious indirectness | serious^3^ | none | 76 | 74 | - | MD 17.27 lower (33.26 to 1.28 lower) | ⊕OOO VERY LOW | IMPORTANT |  |
| **TNF-α (Better indicated by lower values)** | | | | | | | | | | | | |  |
| 1 | randomised trials | serious^1^ | no serious inconsistency | no serious indirectness | no serious imprecision | none | 19 | 17 | - | MD 25.95 lower (34.64 to 17.26 lower) | ⊕⊕⊕O MODERATE | IMPORTANT |  |
| **Hematocrit (Better indicated by lower values)** | | | | | | | | | | | | |  |
| 4 | randomised trials | serious^1^ | no serious inconsistency | no serious indirectness | no serious imprecision | none | 186 | 186 | - | MD 4.58 lower (5.25 to 3.9 lower) | ⊕⊕⊕O MODERATE | IMPORTANT |  |
| **Fibrinogen (Better indicated by lower values)** | | | | | | | | | | | | |  |
| 5 | randomised trials | serious^1^ | serious^2^ | no serious indirectness | no serious imprecision | none | 266 | 266 | - | MD 0.8 lower (1.12 to 0.47 lower) | ⊕⊕OO LOW | IMPORTANT |  |
| **Safety** | | | | | | | | | | | | |  |
| 9 | randomised trials | serious^1^ | no serious inconsistency | no serious indirectness | no serious imprecision | none | 18/482  (3.7%) | 22/478  (4.6%) | RR 0.82 (0.46 to 1.48) | 8 fewer per 1000 (from 25 fewer to 22 more) | ⊕⊕⊕O MODERATE | IMPORTANT |  |

^1^ Poor methodological quality, such as not using blinding or not reporting in detail the specific methods for generating random sequences and allocation concealment.
^2^ Large differences in the effect size of each study point or small overlap of confidence intervals, or large heterogeneity.

^3^ Small sample size or wide confidence interval.
^4^ The funnel plot showed asymmetric distribution and Egger's test indicated possible publication bias.
